# Chemical Profile, Bioactive Constituents and In Vitro Growth Stimulation Properties of Cold-Pressed Hemp Seed Oils from Romanian Varieties: In Vitro and In Silico Evaluation

**DOI:** 10.3390/plants14223465

**Published:** 2025-11-13

**Authors:** Doris Floares (Oarga), Diana Obistioiu, Anca Hulea, Mukhtar Adeiza Suleiman, Iuliana Popescu, Ciprian Buzna, Adina Berbecea, Ersilia Alexa, Cristina Dehelean, Isidora Radulov

**Affiliations:** 1 Faculty of Agriculture, University of Life Sciences “King Mihai I” from Timisoara, Calea Aradului 119, 300645 Timisoara, Romania; doris.oarga@usvt.ro (D.F.); anca.hulea@usvt.ro (A.H.); iuliana_popescu@usvt.ro (I.P.); adina_berbecea@usvt.ro (A.B.); isidora_radulov@usvt.ro (I.R.); 2 Faculty of Life Science, Department of Biochemistry, Ahmadu Bello University, Zaria 810107, Kaduna State, Nigeria; masuleiman@abu.edu.ng; 3 Agricultural Research and Development Station, Lovrin, Principala Street No. 200, 307260 Lovrin, Romania; ciprian.buzna@scdalovrin.com; 4 Faculty of Food Engineering, University of Life Sciences “King Mihail I” from Timisoara, Calea Aradului 119, 300645 Timisoara, Romania; ersiliaalexa@usvt.ro; 5 Faculty of Pharmacy, “Victor Babes” University of Medicine and Pharmacy, 2 Eftimie Murgu Square, 300041 Timisoara, Romania; cadehelean@umft.ro

**Keywords:** *Cannabis sativa*, hemp seed oil, fatty acids, antioxidant activity, in vitro probiotic growth, *Lactobacillus*, molecular docking

## Abstract

Industrial hemp (*Cannabis sativa* L.; *Cannabaceae*), traditionally cultivated for fiber, also represents a valuable source of nutrient-rich seed oil. In this study, cold-pressed hemp seed oils from three Romanian varieties (Teodora, Silvana, and Armanca) were evaluated for their fatty acid composition, minor bioactive constituents, antioxidant activity, growth-promoting property toward probiotic strains in vitro, and molecular docking interactions with probiotic targets. Gas chromatography revealed a fatty acid profile dominated by linoleic (49.4–51.9%), oleic (16.3–22.8%), and α-linolenic acids (9.8–14.4%), resulting in favorable PUFA/SFA ratios (5.17–6.39) and ω-6/ω-3 ratios (3.93–5.53). The oils also contained phenolics (118–160 mg GAE/kg), chlorophylls (6.18–8.31 mg/kg), and carotenoids (2.58–3.37 mg/kg), which contributed to their antioxidant activity (DPPH inhibition 35.92 µM TE/100 g–43.37 µM TE/100 g). Broth microdilution assays against *Lacticaseibacillus rhamnosus* GG, *L. paracasei* ATCC BAA-52, and *L. acidophilus* ATCC 4356 demonstrated strain- and dose-dependent potential to promote probiotic growth under in vitro conditions. While *L. rhamnosus* and *L. paracasei* were inhibited at low concentrations and only mildly stimulated at higher levels, *L. acidophilus* showed robust growth promotion, reaching +54.7% effect and CP = 1.55 with Teodora oil at 16 mg/mL. Molecular docking highlighted strong binding affinities of γ-linolenic and linoleic acids with key metabolic enzymes involved in probiotic metabolism (hydratase, enolase, glyceraldehyde-3-phosphate dehydrogenase, ribonucleoside hydrolase), forming stable hydrophilic and hydrophobic interactions which are explored in defining the stability of the ligand-protein complexes. These results indicate that both major fatty acids and minor bioactive constituents contribute to the nutritional and antioxidant value of Romanian hemp seed oils and reveal a potential to promote probiotic growth under in vitro conditions, as supported by complementary in silico evidence.

## 1. Introduction

Industrial hemp (*Cannabis sativa* L.; Cannabaceae), traditionally cultivated for its fibrous biomass, has emerged as a nutritionally valuable crop. Historically, this versatile plant has been exploited for a wide array of applications—including seeds, oils, textiles, pharmaceuticals, dietary supplements, cosmetics, narcotics, and, more recently, sustainable innovations such as insulation materials, biocomposites, and biodiesel—addressing the global imperative for renewable energy sources [1,2,3,4].

Agronomically, hemp is recognized for its high yield potential and minimal input requirements. Its inherent tolerance to drought and pests renders it a low-maintenance crop. Environmentally, hemp cultivation is advantageous due to its reduced water demand, lack of pesticide dependency, and natural weed suppression, which collectively diminish the need for chemical interventions [5]. Furthermore, industrial hemp contributes significantly to carbon sequestration, owing to its rapid biomass accumulation in agroecological systems [6].

Hemp seed oil is distinguished by its favorable lipid composition, notably its high content of polyunsaturated fatty acids (PUFAs), including linoleic acid (ω-6) and α-linolenic acid (ω-3), typically in a ratio ranging from 3:1 to 5:1. This ratio mirrors those found in traditional Japanese and Mediterranean diets, which are epidemiologically linked to reduced cardiovascular risk [7,8]. As essential fatty acids cannot be synthesized endogenously, dietary intake is crucial [9]. Hemp oil is a significant source of these fatty acids. Hemp oil serves as a rich source of these compounds. Oleic acid, a monounsaturated fatty acid present in hemp seed oil, exhibits considerable variability across cultivars and growing conditions—for example, USO31 grown in Italy contains up to 16.73% oleic acid [10], whereas Earlina8FC cultivated in Poland contains approximately 10.34% [11]. Oleic acid is associated with diverse biological activities, including antioxidant, anti-inflammatory [12], hypocholesterolemic [13], neuroprotective [14], and potential anticancer properties [15], and plays a pivotal role in modulating oxidative stress, membrane fluidity, and immune-neural homeostasis [16].

Beyond its lipid profile, hemp seed oil is enriched with bioactive phytochemicals such as polyphenols, carotenoids, and tocopherols, which contribute to its antioxidant potential and oxidative stability [17,18,19]. These substances contribute to antioxidant activity and may help protect edible oils from lipid oxidation. In the human body, they are associated with significant biological functions, including antioxidant and anti-inflammatory actions [20,21]. The oxidation process can be enhanced by a considerable concentration of chlorophyll, a natural pigment found in hemp seed oil, which is also obtained during cold pressing [22].

Recent investigations have highlighted the modulatory effects of hemp seeds and their extracts on gut microbiota composition [23,24,25]. The gut microbiome is a complex community of microorganisms associated with enhanced digestion and efficient nutrient utilization [26]. Dietary inclusion of hemp seeds has been shown to enhance populations of beneficial bacteria such as *Lactobacilli*, *Clostridiaceae*, *Rikenellaceae*, *Bifidobacterium bifidum*, and *Bacteroides fragilis* [23,24,25,27]. Conversely, there has been a noted decrease in coliform bacteria [25] and other opportunistic bacteria, including *Bilophila wadsworthia* and *Desulfovibrio* [23]. The fiber fraction of hemp seeds, particularly xylose-rich hemicellulose, appears to exert prebiotic effects [28,29]. Additionally, ω-3 and ω-6 fatty acids in hemp oil may influence microbial diversity and support metabolic health [24,30,31].

From an industrial perspective, various extraction techniques are employed to obtain hemp seed oil [32]. Cold pressing remains the preferred method due to its solvent-free operation and low-temperature processing, which preserves thermolabile compounds despite lower yields. Alternative methods include supercritical fluid extraction (SFE), ultrasound-assisted extraction (UAE), and microwave-assisted extraction [33,34,35]. Cold pressing is also economically and environmentally favorable [33,36].

The emergence and utilization of computational biology tools to assess the molecular interactions between identified natural compounds and putative microbial proteins has improved the mechanistic insight into the biological activities of natural products [37,38]. Virtual screening techniques, including molecular docking and binding energy calculations, offer mechanistic insights into the bioactivity of natural products and are increasingly integrated into drug discovery pipelines [39,40]. However, the current study sort to hypothesize the possibility of interactions between the identified fatty acids and putative microbial proteins defined with in silico characterization.

Although Romania has a longstanding tradition in hemp cultivation and varietal development, limited data exist on its native strains. This study focuses on three Romanian dioecious cultivars—Teodora (THSO), Silvana (SHSO), and Armanca (AHSO)—developed at the Lovrin Agricultural Research and Development Station. This study offers the first comparative analysis of cold-pressed hemp seed oils from three Romanian cultivars. It covers their chemical makeup, pigment and phenolic levels, antioxidant activity, and in vitro effects on probiotic growth, all supported by in silico molecular docking studies. This study is critical because it links the biochemical makeup of locally grown varieties to their potential nutritional and functional uses. This connection helps improve Romanian hemp germplasm and supports future advances in nutraceuticals.

## 2. Results

### 2.1. Quantification of Fatty Acids

The profile of fatty acids identified in the analyzed *Cannabis* seed oils is presented in Table 1, showing the presence of fifteen distinct fatty acids.

The predominant fatty acids identified across all samples, listed from highest to lowest concentration, were linoleic acid (C18:2ω-6), oleic acid (C18:1ω-9), α-linolenic acid (C18:3ω-3), palmitic acid (C16:0), stearic acid (C18:0), γ-Linolenic acid (C18:3ω-6) and vaccenic acid (C18:1, ω-7). Significant differences (*p* < 0.05) were noted among the varieties for most fatty acids analyzed, except palmitoleic acid, heptadecanoic acid, eicosenoic acid, behenic acid, and lignoceric acid, which did not exhibit statistically significant differences (*p* > 0.05).

**Figure 1:**
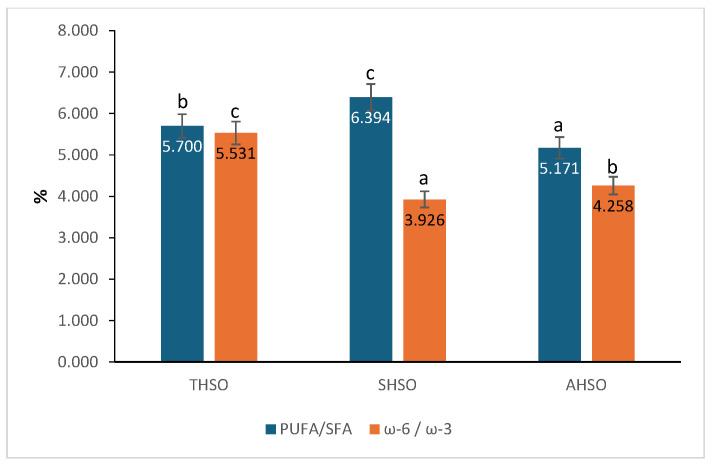
PUFA/SFA and ω-6/ω-3 ratios in hemp seed oil. Hemp seed oil: THSO–Teodora; SHSO–Silvana; AHSO–Armanca. Results are presented as the mean of three replicates ± standard deviation (SD) (*n* = 3). Different lowercase letters (a–c) indicate statistically significant differences (*p* < 0.05), as determined by ANOVA. PUFA/SFA: all pairs < 0.001; ω-6/ω-3: all pairs < 0.001.

The fatty acids were categorized into saturated fatty acids (SFA), monounsaturated fatty acids (MUFA), and polyunsaturated fatty acids (PUFA). SFA exhibited the lowest values, with palmitic acid (6.531–7.895%) and stearic acid (3.194–3.575%) being the predominant saturated fatty acids across all varieties. MUFA was dominated by oleic acid (16.343–22.780%), while PUFA constituted the most substantial portion of the oil, with linoleic acid (49.424–51.858%) and α-linolenic acid (9.852–14.365%) being the most prevalent. Significant differences (*p* < 0.05) were observed among the varieties in the SFA, MUFA, and PUFA fractions. The PUFA/SFA ratio in the oil extracted from variety SHSO (6.394) was the highest, followed by variety THSO (5.700) and variety AHSO (5.171). Statistically significant differences (*p* < 0.05) were observed among the three varieties (Figure 1).

The findings for ω-3 and ω-6 show that the extracted oils mainly consist of unsaturated fatty acids, particularly α-linolenic acid and linoleic acid, which are the primary fatty acids. Among the varieties, variety SHSO showed the highest ω-3 content at 14.365%, followed by variety AHSO at 12.600%, and variety THSO, which had the lowest value of 9.852%. Statistically significant differences (*p* < 0.05) were observed among the varieties. About ω-6 content, statistically significant differences (*p* < 0.05) were observed among the samples. The SHSO sample exhibited the highest ω-6 concentration (56.388%), followed by THSO with 54.486%. At the same time, AHSO recorded the lowest value, 53.656%. The ω-6/ω-3 ratio was significantly influenced by the variety and followed the order: THSO > AHSO > SHSO.

### 2.2. Total Phenolic Content and DPPH Scavenging Activity

**Figure 2:**
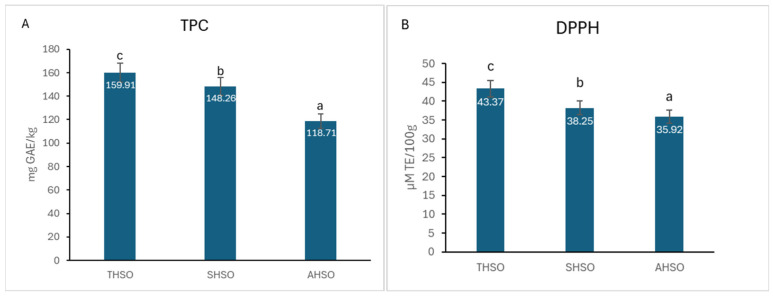
(**A**) Total phenolic content (TPC) and (**B**) DPPH scavenging activity of hemp seed oil. Hemp seed oil: THSO–Teodora; SHSO–Silvana; AHSO–Armanca. Results are presented as the mean of three replicates ± standard deviation (SD) (*n* = 3). Different lowercase letters (a–c) indicate statistically significant differences (*p* < 0.05), as determined by ANOVA. TPC: all pairs < 0.001; DPPH: all pairs < 0.001.

The levels of total phenolics and DPPH scavenging activity in the different hemp seed oil samples were quantified and are presented in Figure 2.

The total phenolic content in the various hemp seed oil samples ranged from 118.71 mg GAE/kg to 159.91 mg GAE/kg, exhibiting statistically significant differences (*p* < 0.05) among the samples. In terms of DPPH radical scavenging activity, the results ranged from 35.92 µM TE/100 g in sample AHSO to 38.25 µM TE/100 g in sample SHSO and 43.37 µM TE/100 g in sample THSO, with significant differences (*p* < 0.05) observed between them.

### 2.3. Total Chlorophylls and Carotenoids Content of Hemp Seed Oil

**Figure 3:**
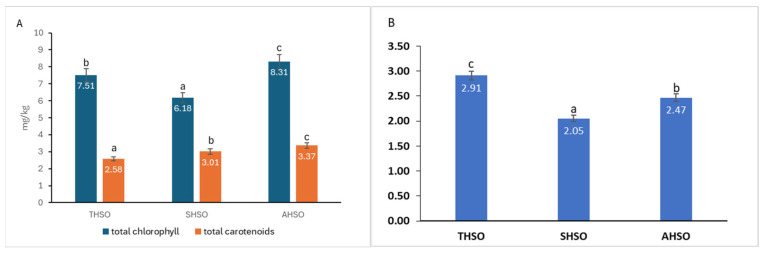
(**A**) Total chlorophylls and carotenoids content of hemp seed oil. (**B**) Ratio of total chlorophyll to total carotenoids. Hemp seed oil: THSO–Teodora; SHSO–Silvana; AHSO–Armanca. Results are presented as the mean of three replicates ± standard deviation (SD) (*n* = 3). Different lowercase letters (a–c) indicate statistically significant differences (*p* < 0.05), as determined by ANOVA. Total chlorophyll: all pairs < 0.001; total carotenoids: all pairs < 0.001; total chlorophyll/total carotenoids: all pairs < 0.001.

The levels of chlorophylls and carotenoids in the various hemp seed oil samples were measured and are shown in Figure 3.

The total chlorophyll content was determined to be between 6.18 mg/kg and 8.31 mg/kg of oil, also showing statistically significant variation (*p* < 0.05) among the samples. Likewise, the total carotenoid content varied from 2.58 mg/kg to 3.37 mg/kg of oil, with significant differences (*p* < 0.05) noted among the tested samples. The total chlorophyll/total carotenoids ratio decreases in order THSO > AHSO > SHSO.

Pearson’s correlation analysis revealed a statistically significant and robust positive relationship between TPC and DPPH values (r = 0.896, p = 0.01), with a 95% confidence interval ranging from 0.572 to 0.978. This finding underscores the pivotal role of phenolic compounds in modulating the antioxidant capacity of hemp seed oils. The high correlation coefficient suggests that phenolics are likely the primary contributors to the observed radical scavenging activity, complementing the antioxidant effects of unsaturated fatty acids and pigments such as carotenoids and chlorophylls. These results are consistent with previous studies on plant-derived oils, where phenolic compounds have been shown to enhance oxidative stability and confer health-promoting properties through their free radical neutralization mechanisms [17]. Taken together, the strong TPC–DPPH correlation reinforces the relevance of total phenolic content as a reliable indicator of antioxidant potential in hemp seed oils. This relationship not only validates the analytical approach employed but also provides a valuable criterion for selecting cultivars with enhanced nutraceutical value. Detailed correlation statistics, including confidence intervals, are provided in Supplementary Data S1.

### 2.4. Evaluation of the In Vitro Probiotic Growth-Promoting Effect

The OD values were used in formulas (5) and (6) and the results are presented in Table 2 as mean ± SD values of %Effect of hemp seed varieties on probiotic selected strains and Table 3 as Mean ± SD values of the Potentiation Coefficient (CP).

In case of *L. rhamnosus,* at lower concentrations (0.3–5 mg/mL), all oils exerted clear inhibitory effects, ranging from −43.81% (THSO) to −51.32% (SHSO). As the dose increased, the inhibitory response diminished, shifting towards neutrality or slight stimulation at 9–16 mg/mL. SHSO showed the most pronounced stimulation (+16.83% at 16 mg/mL), followed by THSO (+2.43%), while AHSO remained weakly inhibitory (−6.88%).

Similar to *L. rhamnosus, L. paracasei* was strongly inhibited at 0.3 mg/mL, with THSO showing the most pronounced suppression (−49.62%). However, inhibition decreased with increasing concentrations, and at 16 mg/mL, THSO significantly promoted growth (+20.92%). In contrast, SHSO and AHSO remained inhibitory at all concentrations (up to −8.32%).

Unlike the other strains, *L. acidophilus* responded positively to hemp oils starting at relatively low doses. THSO showed a dose-dependent stimulation, from 1.46% at 0.6 mg/mL to +54.74% at 16 mg/mL, highlighting a dramatic growth-promoting effect. SHSO and AHSO were less effective, producing modest stimulation (+10.95% and +2.92% at 16 mg/mL, respectively). These results position *L. acidophilus* as the most responsive probiotic to hemp oil supplementation, particularly when treated with THSO.

The CP values obtained for the three probiotic strains (*Lacticaseibacillus rhamnosus* GG, *Lactobacillus paracasei subsp. paracasei* ATCC BAA-52, and *Lactobacillus acidophilus* ATCC 4356) exposed to hemp seed oil fractions are presented in Table 3.

For *L. rhamnosus* GG, CP values generally remained below 1.0 at lower concentrations (0.3–2.5 mg/mL), indicating no potentiation or slight inhibition, except with AHSO at 0.3 mg/mL (0.74). A moderate increase was observed at 16 mg/mL with SHSO (1.17), suggesting strain- and fraction-specific stimulation.

For *L. paracasei* ATCC BAA-52, CP values were close to unity across most concentrations, reflecting neutral effects. The most notable stimulation was observed at 16 mg/mL with THSO (1.21), while SHSO and AHSO fractions showed negligible or inhibitory trends.

In contrast, *L. acidophilus* ATCC 4356 exhibited the highest responsiveness, with CP values consistently > 1.0 for THSO at ≥1.3 mg/mL, reaching 1.55 at 16 mg/mL. SHSO and AHSO fractions also supported growth (up to 1.11 and 1.03, respectively), although to a lesser extent.

Overall, hemp seed oil exerted a strain-dependent in vitro probiotic growth-promoting effect, most pronounced in *L. acidophilus* ATCC 4356, particularly with the total oil fraction (THSO), while *L. rhamnosus* GG and *L. paracasei* ATCC BAA-52 showed weaker or concentration-specific responses.

### 2.5. Molecular Docking Results

**Figure 4:**
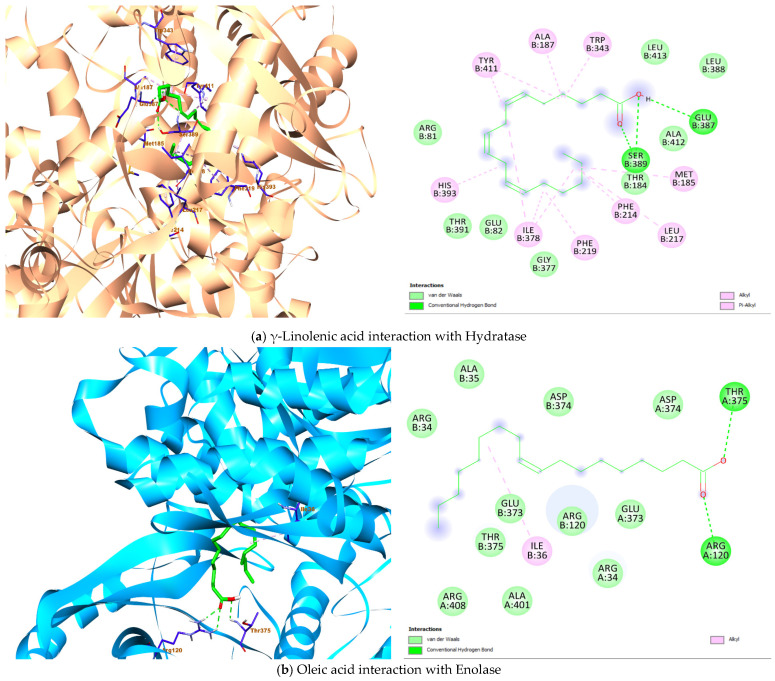

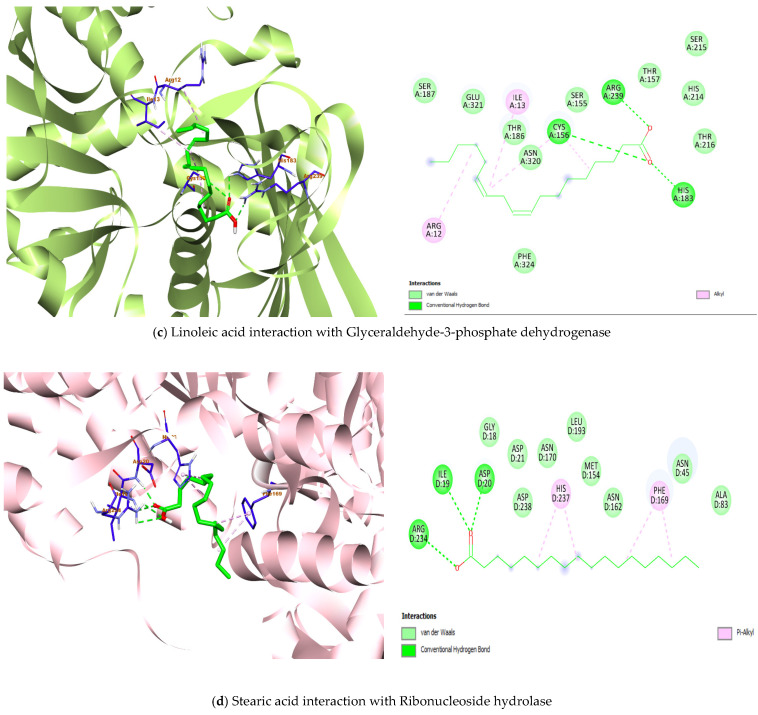
Architecture of the best binding interaction between the compounds and the four *Lactobacillus* protein targets.

The seven (7) fatty acids identified with high abundance in the seed oil of the current study were screened for their docking potential against four *Lactobacillus* strain protein targets to explore the putative inhibitors based on the binding affinity score and interaction properties. The recorded binding affinities between the ligands and receptors (Table 4) displayed interesting binding interactions supported by the docked complexes. γ-Linolenic acid has the binding energy of −7.4 kcal/mol with hydratase and establishes two hydrogen bonds (Glu B:387 and Ser B:389) in the presence of alkyl/pi-alkyl strong interactions (Figure 4a). Furthermore, linoleic acid, α-linoleic acid, oleic acid and stearic acid also showed interesting binding interaction with the hydratase protein target (Table 4).

The highest binding affinity (−5.5 kcal/mol) with 4MKS was observed in the docking result involving linoleic acid, α-linolenic acid and γ-Linolenic acid. Although the strong hydrogen bonds were seen in the molecular interaction of palmitic acid, γ-Linolenic acid, oleic acid and stearic acid, with one or more hydrogen bonds formed, particularly the protein amino acid residues Arg A:120, Asp A:374, Thr A:375, Ala A: 383, Arg B:408 while the interior part of the protein is involved in hydrophobic interaction (Figure 4b).

α-linolenic acid fared better in terms of the binding affinity to 5J9G (−5.6 kcal/mol). More importantly, all seven fatty acids interact strongly with the protein by having both hydrophilic and hydrophobic interactions (Figure 4c). At the ribonucleoside hydrolase protein, the γ-Linolenic acid ligand showed the highest binding affinity value with −5.4 kcal/mol. On the other hand, a more favourable interaction is observed with stearic acid, having three hydrogen bonds (Ile D:19, Asp D:20, Arg D:234) and two alkyl interactions (Phe D: 169, His D:237) (Figure 4d).

## 3. Discussion

The fatty acid composition of hemp seed oils obtained from Romanian cultivars in this study aligns broadly with previously reported profiles from other European varieties, while also revealing distinct quantitative differences. For instance, Mendoza-Perez et al. [41], observed only minor deviations in oils derived from Spanish-grown hemp, whereas Alonso-Esteban et al. [42] reported higher linoleic acid concentrations (54.99–57.36%) in unhulled seed oils, followed by α-linolenic acid (12.85–15.87%) and oleic acid (11.92–17.31%) as the predominant fatty acids. Similarly, Golimowski et al. [43] found comparable fatty acid patterns in Polish cultivars, though with generally lower proportions than those identified in our samples—except for oleic acid, which was notably higher. These variations may be attributed to cultivar-specific traits and the influence of geographic and environmental conditions on lipid biosynthesis.

The nutritional quality of oils is influenced not only by their fatty acid composition but also by the balance between these acids, particularly the ω-6/ω-3 ratio and the PUFA/SFA balance ratio [44]. Lower ω-6/ω-3 ratios (ranging from 2:1 to 5:1) have been shown to have beneficial effects in various conditions, including reduced mortality in heart disease, decreased inflammation in arthritis, and a lower risk of cancer. The optimal ratio may vary depending on the disease, but overall, a lower ω-6/ω-3 ratio is considered more favorable for health [31]. The PUFA/SFA ratio is a widely used indicator of dietary fat quality and its impact on cardiovascular health, with higher values generally associated with better cholesterol profiles and a reduced risk of cardiovascular disease [45].

Siano et al. [46] in their comprehensive analysis of the physicochemical, chemical, and biochemical characteristics of hemp seeds, oil, and flour derived from the monoecious genotype Fedora (*Cannabis sativa* L.), reported a PUFA/SFA ratio of 6.84 in hemp seed oil, closely matching the values obtained in our current study. Similarly, Rosso et al. [47] investigated three Italian hemp cultivars—Carmaenecta, Enectaliana, and Enectarol—grown in Central Italy, assessing their proximate composition, fatty acid profiles, antioxidant activity, total phenolic content, and N-trans-caffeoyltyramine levels. Their reported PUFA/SFA ratios ranged from 5.79 to 6.29, which are comparable to our findings. In contrast, Mendoza-Pérez et al. [41] recorded higher PUFA/SFA ratios, ranging from 7.18 to 7.43, in their characterization of three hemp varieties (Ferimon, Henola, and Uso-31), exceeding the values observed in our study. Regarding ω-6/ω-3 ratios, Islam et al. [48] evaluated the thermal and oxidative stability of cold-pressed oils from fresh and aged Henola seeds, reporting ratios between 1.90 and 3.09—figures that align well with our results. Golimowski et al. [43] compared the cultivars Earlina 8FC, S. Jubileu, and Finola, examining the influence of genotype and pressing conditions on oil yield and quality, and found ω-6/ω-3 ratios ranging from 3.23 to 3.67, consistent with our data. In a subsequent study, Golimowski et al. [11] assessed the impact of bleaching on oils from Finola, Earlina 8FC, and Secuieni Jubileu, reporting ω-6/ω-3 ratios between 3.18 and 3.68, further corroborating the values obtained in our investigation.

Phenolic compounds are valuable minor components present in many vegetable oils, impacting their antioxidant and antimicrobial properties [49]. Siger et al. conducted an analysis of methanolic extracts from various cold-pressed oils, including hemp seed oil, to assess their phenolic composition and antioxidant capacity, reporting a total phenolic content of 2.45 mg CAE/100 g and a DPPH radical scavenging activity of 76.2% for hemp oil [50]. In another study by Kalinowska, cold-pressed vegetable oils—specifically hemp and milk thistle seed oils—were assessed for their chemical composition and antioxidant properties. The total phenolic content in hemp seed oil varied depending on the solvent used for extraction, with values increasing in the following order: methanolic extract (SPE,0.004 mg GAE/g oil) < 80% ethanolic extract (0.011 mg GAE/g oil) < 80% methanolic extract (0.014 mg GAE/g oil) < ethanolic extract (0.047 mg GAE/g oil) < methanolic extract (0.154 mg GAE/g oil) [51]. In the study conducted by Muangrat et al., the authors assessed screw press, accelerated solvent extraction with hexane, and supercritical CO_2_ methods to determine the most effective technique for maximizing cannabidiol (CBD) yield from hemp seed oil. For the screw press method, they reported a total polyphenol content of 0.11 mg GAE/g of oil and a DPPH radical scavenging activity of 1.38 μmol TEAC/g of oil [52]. These values align with those reported in our study.

Lower values than those found in our experiment were reported by Siano et al., who observed a total polyphenol content of 21 mg GAE/kg in oil from the Fedora cultivar hemp and a DPPH inhibition of 8.2% in their study [46]. Similarly, Mansouri et al. reported comparably lower values as well, with a total polyphenol content of 64.42 mg GAE/kg and a DPPH inhibition capacity of 43.67% for raw hemp seed oil, while optimizing the roasting process of Moroccan hemp seeds [53].

Elevated levels of total polyphenols in hemp seed oil have been previously reported by Teh et al. [34]. who investigated the physicochemical and quality attributes of cold-pressed hemp, flax, and canola seed oils from New Zealand, noting a total phenolic content of 188.23 mg GAE/100 g in hemp oil. Similarly, Yu et al. [54] evaluated the antioxidant properties of cold-pressed oils from black caraway, carrot, cranberry, and hemp seeds, reporting a phenolic content of 0.44 mg GAE/g for hemp seed oil—values exceeding those observed in our study. Smeriglio et al. [17] further demonstrated the potent antioxidant activity of cold-pressed oil from the Finola cultivar of *Cannabis sativa* L., with a DPPH radical scavenging capacity of 146.76 mmol TE/100 g and a total phenolic content of 267.5 mg GAE/100 g. In a targeted analysis of minor bioactive compounds and cannabidiolic acid in commercial hemp seed oils, Izzo et al. [55] reported a wide range of polyphenol concentrations, from 22.1 to 160.8 mg GAE/g. Islam et al. [48], in their assessment of oil quality from fresh and stored Henola seeds using differential scanning calorimetry, recorded total phenolic contents between 69.11 and 81.43 mg/100 g and DPPH radical scavenging activities ranging from 6.96 to 9.11 µmol TE/g—values that are broadly consistent with our findings.

Hemp seed oil also contains minor compounds, specifically the pigments chlorophyll and carotenoids. Chlorophylls are lipid-soluble pigments sensitive to heat and light; under unfavorable conditions during oil extraction or storage, they can lead to the breakdown of fatty acids [48]. This results in increased rancidity, diminished oil quality, and a reduced shelf life. Conversely, carotenoids—another type of pigment that contributes to the oil’s yellow coloration—serve a beneficial antioxidant function [56], aiding in the reduction in rancidity and helping to maintain color stability during storage [18]. Studies have shown that a ratio below 2.5 is favorable, suggesting a better antioxidant profile and improved pigment balance, which are advantageous for oil stability and shelf life [57].

Our findings are consistent with those reported by Izzo et al. [55] who investigated thirteen commercial hemp seed oil samples from the Italian market to characterize their chemical composition and quality parameters, to evaluate biochemical variability and promote their dietary relevance. Their analysis revealed substantial variation in total chlorophyll content, ranging from 0.41 to 4.81 mg/kg, with an average of 1.46 mg/kg across samples. Carotenoid levels spanned from 0.18 to 1.73 mg/kg, yielding a mean concentration of 0.52 mg/kg, while chlorophyll-to-carotenoid ratios varied between 1.98 and 3.83. Muangrat et al. investigated various extraction methods—screw press, hexane-based accelerated solvent extraction, and supercritical CO_2_—to identify the most effective technique for maximizing CBD yield from Thailand hemp seed oil. Using the screw press method, they determined a chlorophyll a + b content of 3.70 µg/g oil and a carotene content of 11.01 µg/g oil [52].

Values exceeding those obtained in our study were reported by Tura et al. [22], who assessed the quality attributes and chemical composition of thirteen commercial hemp seed oils. Their analysis revealed a broad range of total chlorophyll content, from 0.78 to 75.7 mg/kg of oil, and total carotenoid levels ranging from 2.53 to 33.93 mg/kg. In a related study, Mansouri et al. [53] applied response surface methodology to optimize the roasting conditions of Moroccan hemp seeds and compared the physicochemical characteristics of raw versus roasted samples. Their findings indicated a modest decline in pigment concentrations following roasting, with chlorophyll decreasing from 39.10 to 36.54 mg/kg and carotenoids from 10.55 to 10.05 mg/kg of oil.

A significant positive relationship between total phenolic content and DPPH radical-scavenging activity in the hemp seed oils tested indicates that phenolic compounds significantly influence the antioxidant potential of these samples. The strong correlation aligns with earlier findings that polyphenols in plant-based oils help neutralize free radicals by donating hydrogen atoms or electrons to stabilize reactive species. Differences in antioxidant capacity across cultivars are mainly due to variations in phenolic content, rather than differences in unsaturated fatty acids or pigments.

The chemical composition of the hemp oil is correlated with potential health benefits. The positive impact of using hemp seeds in the diet was demonstrated. It seems that this contributes to the prevention of the development of numerous chronic diseases [8,58,59,60]. Recent scientific research demonstrated that many of these diseases are associated with imbalances or problems in the gut microbiota. Even though the gut microbiome is not the sole cause of these diseases, alterations in its composition can influence inflammation, immune response and metabolism [61,62,63]. Maintaining a healthy and balanced gut microbiome is crucial for overall well-being and disease prevention. The present study demonstrated different in vitro probiotic-stimulating activity depending on the type of hemp oil and the probiotic strain. While *L. rhamnosus* and *L. paracasei* were inhibited at lower concentrations and only slightly stimulated at higher doses, *L. acidophilus* growth was generally stimulated in the presence of hemp oils. The greater ability of *L. acidophilus* to assimilate fatty acids and adapt to redox conditions may be the cause of this strain-specific stimulation. Unsaturated fatty acids can be incorporated into the membranes of *Lactobacillus* species, improving the permeability and resistance to oxidative stress. The strong binding affinities of γ-linolenic and linoleic acids to key metabolic enzymes (hydratase, enolase, glyceraldehyde-3-phosphate dehydrogenase, and ribonucleoside hydrolase) suggest that these compounds may act not only as metabolic substrates but also as modulators of cellular redox balance. Therefore, enhanced energy metabolism and oxidative stress resilience mediated by PUFA uptake and enzyme interaction may be the cause of the observed increase in optical density and CP values for *L. acidophilus*. This process is consistent with research that shows fatty acid supplementation with ω-6 and ω-3 improves lactic acid bacteria’s membrane adaptation and redox homeostasis, the supplementation of culture media with PUFAs has been shown to alter membrane lipid composition in lactobacilli [64], while stress adaptation studies confirm that changes in the degree of fatty acid unsaturation regulate membrane fluidity and cell survival [65]. Oleic and linoleic acids are well-established as essential growth factors for specific *Lactobacillus* strains [63] and desiccation tolerance in *L. buchneri* has been associated with alterations in membrane fatty acid composition [66] These observations reinforce the hypothesis that the fatty acid profile of hemp seed oils plays a critical role in mediating strain-specific in vitro probiotic growth-promoting effects. Among the three oils evaluated, THSO demonstrated the most pronounced stimulation of *Lactobacillus acidophilus*, with a growth increase of up to 54.7% and a CP value of 1.55 at a concentration of 16 mg/mL. Notably, THSO also exhibited the highest levels of phenolic compounds and antioxidant activity, suggesting a potential synergistic interaction between its fatty acid constituents and antioxidant compounds in enhancing probiotic performance.

*Lactobacillus* hydratase (4IA6) is reported to be susceptible to the biohydrogenation of unsaturated fatty acids such as linoleic acid [67]. The present molecular docking study showed linoleic acid, oleic acid and the isomeric forms of linolenic acid (α, γ) having potential to exercise polar interactions with the amino acid residues Glu387 and Ser389, while also fairing in a number of nonpolar interactions with long hydrocarbon chain of the fatty acids involving Tyr411 (Table 4). The interaction with Tyr411 is critical as it provides stability to the ligand-protein complex [68]. The other two glycolytic enzymes, enolase (4MKS) and glyceraldehyde-3-phosphate dehydrogenase (5J9G) are linked to plasminogen/plasmin system which are exploited by several microorganism to increase their pathogenicity [69]. The hydrogen bonds (Thr375, Arg120) formed by oleic acid with enolase could be involved in strengthening the binding through polar interaction. In a similar trend, the observed hydrogen bonds (Cys156, His183, Arg239) by linoleic acid with glyceraldehyde-3-phosphate dehydrogenase are valuable depiction of the extend this complex could be stabilized. Consequently, the fatty acids of *C. sativa* seed oil could potentially be able to exhibit inhibitory property as explored by the good binding affinity and strong binding interaction (Figure 4, Table 4). Additionally, the inhibitory potential of the acids and ribonuleoside hydrolase (8QND) is favored with strong hydrophobic interaction. More so, stearic acid demonstrated three hydrogen bonds with the amino acid residues in the binding pocket of the protein (Asp D:20). The current molecular docking study revealed γ-Linolenic acid C18:3 possess the highest affinity (−7.4, −5.5, −5.0, −5.4 kcal/mol) and interacts fairly well with the four *Lactobacillus* protein targets, respectively. Thus, the fatty acids were strongly interacting with the amino acids in the flexible loop of the protein [70]. Overall, the understanding of the binding affinities and binding interactions of the ligand-protein complexes hypothesized in this study provides important plausible insights that could be explored for further utilization of the *C. sativa* seed oil inhibitory property.

## 4. Materials and Methods

### 4.1. Chemicals

The reagents, including ethanol (Cat. no. 34852-1L-M), Folin–Ciocalteu (Cat. no. 47641-500mL-F), gallic acid, standard (Cat. no. 398225-100G), 1,1-diphenyl-2-picrylhydrazyl (DPPH) (Cat. no D9132-1G), potassium hydroxide (Cat. no. 221473-500G), potassium hydrogen sulfate (Cat. no. 223476-500G), and hexane (Cat. no. 139386-2.5L), were obtained from Sigma–Aldrich Chemie GmbH in Munich, Germany. Sodium carbonate (Cat. no. 11646929), methanol (Cat. no. 12313607), and diethyl ether (Cat. no. 2362-1L) were sourced from Geyer GmbH in Renningen, Germany. Trolox (Cat. no 218940010) was obtained from Thermo Fisher Scientific, Janssen Pharmaceuticalaan 3a, Geel, 2440 Belgium. All reagents used for the chemical analysis were of analytical grade. All culture media used in microbiological analyses were purchased from Oxoid Limited, Thermo Fisher Scientific Inc. (Waltham, MA, USA).

### 4.2. Plant Material

The hemp seed varieties examined in this study, Teodora (THSO), Silvana (SHSO), and Armanca (AHSO), were cultivated at the Lovrin Agricultural Research and Development Station (coordinates: 45°57′03″ N, 20°46′32″ E). The oils were obtained through a cold-press technique performed at the station’s specialized Laboratory of Genetics and Breeding of dioecious hemp using a PU10 hydraulic oil press, with a temperature maintained below 50 °C. The oil was then left for 24 h of decantation.

### 4.3. Quantification of Fatty Acids

Before gas chromatography (GC) analysis, the fatty acids in the oils were converted into methyl esters (FAMEs) using a 2 M KOH solution in methanol, following the procedure described by Popescu et al. [71]. The oil was mixed with 4 mL of hexane, followed by the addition of 400 µL of KOH solution. The mixture was vortexed for 5 min. Next, 500 mg of KHSO_4_ was added. The organic layers were separated by centrifugation at 3000 rpm for 15 min and then transferred to vials for GC analysis. FAMEs were analyzed with a Shimadzu GC-MS QP 2010Plus instrument (Shimadzu Corporation, Tokyo, Japan), which featured a mass spectrometer (MS) detector and an AT-5MS capillary column (30 m × 0.32 mm × 0.25 µm). The injection volume was set to 1.0 µL, and the injection port temperature was maintained at 250 °C. The carrier gas, helium, was set to a flow rate of 1.8 mL/min with a split ratio of 1:100. The oven was initially set to 100 °C and held for 2 min, then ramped at 3 °C/min to 150 °C (held 2 min), followed by a ramp of 5 °C/min to 250 °C and a final ramp of 10 °C/min to 300 °C. The MS parameters included an ion source temperature of 210 °C and an interface temperature of 255 °C. Identification and quantification of FAMEs were conducted using the NIST05 library and the area normalization method. The results of the fatty acid (FA) analysis are reported as a percentage of the total FAMEs.

### 4.4. The Preparation of Oil Extracts

To prepare the extract, 2.5 g of oil was thoroughly mixed with 5 mL of hexane and 5 mL of an 80% methanol solution. The mixture was then centrifuged at 5000 rpm for 10 min, resulting in the separation of two distinct phases. The lower phase, containing the phenolic compounds, was collected and stored at 4 °C for further analysis [72].

### 4.5. Determination of Total Polyphenol Content

The total polyphenol content in three hemp seed oil extracts was evaluated using the Folin–Ciocalteu assay [73]. 0.5 mL from each extract was mixed with 1.25 mL of Folin–Ciocalteu reagent, diluted to 1:10 in water. This mixture was allowed to sit at room temperature for five minutes before the addition of 1 mL of a 60 mg/mL Na_2_CO_3_ solution. Subsequently, each sample was incubated for 30 min at 50 °C in a Memmert INB500 thermostat (Memmert GmbH & Co. KG—Schwabach, Germany). After incubation, absorbance was measured at 750 nm using a Specord 205 UV-VIS spectrophotometer (Analytik Jena AG, Jena, Germany). Each sample was analyzed in triplicate, with results expressed as mean ± standard deviation in mg GAE/kg. A calibration curve was constructed with gallic acid as a standard across a range of 0–200 µg/mL (R^2^ = 0.9986).

### 4.6. DPPH Radical Scavenging Activity Assay

A 0.3 mM solution of 1,1-diphenyl-2-picrylhydrazyl (DPPH) in ethanol was created to assess antioxidant activity [74]. To this, 1 mL of the oil extract (250 mg/mL) was added to 2.5 mL of the DPPH solution, and the blend was thoroughly shaken before being kept in the dark at room temperature for 30 min. After incubation, the absorbance was measured at 518 nm using a UV-VIS spectrophotometer (Specord 205; Analytik Jena AG, Jena, Germany) [73]. The control sample was made by substituting the oil extract with 70% ethanol. Each sample was analyzed in triplicate, and the average result was noted. The antioxidant capacity was quantified as the percentage of radical scavenging activity (RSA), calculated using the following formula:

(1) $$\mathrm{RSA}\;(\%)\;=\;(\frac{Ac-As}{Ac})\times 100\;$$

where: A_c_ = absorbance value of the control sample and A_s_ = absorbance value of the extract sample.

Antioxidant activity was reported as µM Trolox equivalents per 100 g by constructing a DPPH–Trolox calibration (1.0–25 µg mL^−1^), interpolating Trolox-equivalent (TE) for samples from the regression, and converting to µM using Trolox’s molar mass and the sample solution concentration [75].

### 4.7. Chlorophyll and Carotenoids Determination

The concentrations of chlorophyll and carotenoids in the hemp seed oil samples were measured using the procedure described by Izzo [55]. Each oil sample, weighing 0.100 g, was placed in a centrifuge tube, with the total volume adjusted to 3 mL using absolute diethyl ether. The mixture was thoroughly vortexed and then subjected to ultrasound treatment for one minute. Absorbance readings were taken at 470 nm, 640 nm, and 663 nm using a UV-VIS spectrophotometer (Specord 205; Analytik Jena AG, Jena, Germany), with the solvent serving as a blank reference. The pigment concentrations (µg/mL) in the oils were calculated using the appropriate equation.

(2) Chlorophyll a (Chl a) = 9.93 × A_663_ − 0.78 × A_640_

(3) Chlorophyll b (Chl b) = 17.60 × A _640_ − 2.81 × A_663_

(4) Chlorophyll a + b = 7.12 × A _663_ + 16.80 × A_640_

(5) $$\mathrm{Total}\;\mathrm{carotenoids}=\frac{100\times A470-0.52\times Chla-7.25\times Chlb}{226}$$

where: A_470_ = absorbance readings at 470 nm; A_640_ = absorbance readings at 640 nm; A_663_ = absorbance readings at 663 nm.

Pigment content in the oil was expressed as mg of pigment per kg of oil (mg/kg) using the equation.

(6) $$c=\frac{c1\times V\times R}{G}$$

where: c is the amount of pigment in oil (mg/kg), c_1_ is the pigment concentration (μg/mL), V is the initial volume (mL), R is the dilution factor (if applicable), G is the measured mass of oil (g).

### 4.8. Evaluation of the In Vitro Probiotic Growth-Promoting Effect

Measurement of O.D. [76] to determine cell density is advantageous over the plate count method used previously [77] because it is a more rapid and straightforward technique to determine probiotic-stimulating activity. Preliminary experiments revealed that the O.D. method yielded consistent results similar to those obtained by the plate count method.

The probiotic growth-promoting activity of the three hemp seed oils (THSO, SHSO, AHSO) was tested against *Lactobacillus rhamnosus* GG, *L. paracasei* ATCC BAA-52, and *L. acidophilus* ATCC 4356 at final concentrations ranging from 0.3 to 16 mg/mL. The results showed clear strain- and dose-dependent effects, with marked differences among the three oil varieties.

Broth microdilution assays were carried out in sterile 96-well flat-bottom plates (SPL Life Sciences, Korea). Hemp seed oil (0.3–16 mg/mL), emulsified with 0.1% Tween 80 (CAS 9005-65-6; Merck KGaA, Darmstadt, Germany) in MRS broth (Oxoid, CM0359B), was mixed with probiotic suspensions (*L. rhamnosus* GG, *L. paracasei* ATCC BAA-52, *L. acidophilus* ATCC 4356). After incubation at 37 °C for 24 h, bacterial growth was quantified by optical density measurements at 600 nm using a microplate reader (BIORAD PR 1100, Hercules, CA, USA). The control wells contained MRS broth supplemented with 0.1% Tween 80, the same concentration used for oil emulsification. For each oil concentration, sterile media blanks consisting of MRS broth supplemented with 0.1% (*v*/*v*) Tween 80 and the corresponding oil concentration (without bacterial inoculum) were prepared in parallel to account for background light scattering from the emulsified oils. The mean OD_600_ value of each blank was subtracted from the corresponding inoculated sample to obtain the corrected OD_600_. All triplicate OD_600_ values reported in Supplementary File S3 represent these blank-corrected readings, which were subsequently used to calculate %Effect and Potentiation Coefficient (CP). All statistical analyses were performed using the blank-corrected OD_600_ data.

Two indices were calculated to evaluate the in vitro probiotic growth-promoting effect: % Effect versus control and the Potentiation Coefficient (CP). The % Effect versus control was calculated using the OD measurements with the following Equation (7):

(7) $$\%Effect=\frac{ODsample-ODcontrol}{ODcontrol}\times 100$$

The Potentiation Coefficient (CP) was calculated for each concentration according to the formula:

(8) $$CP=\frac{ODsample}{ODcontrol}$$

### 4.9. Molecular Docking Protocol

The crystal structures of four proteins were retrieved from protein data bank, www.rcsb.org (accessed on 13 July 2025) (PDB ID: 4IA6, 4MKS, 5J9G, 8QND) [78]. The 15 fatty acids identified in the oil were downloaded from https://pubchem.ncbi.nlm.nih.gov/ (accessed on 14 July 2025) in SDF format [79].

Molecular docking was performed using PyRx–Python Prescription 0.8. The proteins were prepared as macromolecules and converted to .pdbqt, while the fatty acids were also prepared for docking and converted to ligands in .pdbqt format. The docking simulations were carried out on whole proteins. The grid box coordinates were set to cover the entire protein structures to allow possible binding pose of sites other than the proteins active site, 4AI6 (center X = −43.2116, Y = 21.1678, Z = −24.2391; size X = 108.2871, Y = 85.1366, Z = 75.6535), 4MKS (center X = −45.8371, Y = −41.6539, Z = 4.0849, size X = 75.5076, Y = 72.6848, Z = 60.3495), 5J9G (center X = 18.1954, Y = 39.5496, Z = 1.9677; size X = 76.7661, Y = 75.6436, Z = 82.8988) and 8QND (center X = 52.0286, Y = 2.5919, Z = 18.2901; size X = 92.9826, Y = 57.0042, Z = 99.1795). The 3D and 2D binding interaction between the protein and ligand was visualised in BIOVIA Discovery Studio Visualizer v21.1.0.20298 (BIOVIA, San Diego, CA, USA). The docking exhaustiveness was set at 8.

### 4.10. Statistical Analysis

Statistical analysis was performed using JASP software (version 0.19.3.0). For chemical composition, pigment, total phenolic, and antioxidant data, one-way ANOVA followed by Tukey’s post hoc test was applied to determine significant differences among cultivars, with the significance level set at *p* < 0.05. For microbiological growth assays, a two-way ANOVA was performed to evaluate the effects of oil type and con-centration, as well as their interaction (oil × concentration) on %Effect and Potentiation Coefficient (CP) values. When significant effects were observed, pairwise comparisons were conducted using Tukey’s post hoc test (*p* < 0.05). The assumptions of normality and homogeneity of variances were assessed prior to ANOVA using Shapiro–Wilk and Levene’s tests, respectively. When these assumptions were not met, data were appropriately transformed to improve compliance before analysis. All assays were conducted in triplicate, and results are expressed as mean ± standard deviation (SD). Statistically significant differences among groups are indicated by lowercase superscript letters in the tables and figures.

## 5. Conclusions

This study demonstrated that cold-pressed hemp seed oils from Romanian varieties are rich in polyunsaturated fatty acids, with favorable PUFA/SFA and ω-6/ω-3 ratios that support their role as health-promoting dietary fats. Alongside their fatty acid profile, the oils contain phenolic compounds, chlorophylls, and carotenoids, which contribute to antioxidant stability and may synergize with PUFAs to enhance biological effects. The probiotic growth-promoting assays revealed clear strain-dependent responses, with *L. acidophilus* showing robust stimulation in contrast to the weaker effects observed in *L. rhamnosus* and *L. paracasei*. These functional outcomes were consistent with molecular docking results, where γ-linolenic and linoleic acids displayed strong binding interactions with key metabolic enzymes of *Lactobacillus*, involved in probiotic metabolism, suggesting a biochemical basis for strain-specific benefits. Taken together, the integrated chemical, microbiological, and computational analyses provide strong evidence that hemp seed oils may act as multifunctional nutraceuticals, combining antioxidant potential with proven in vitro probiotic growth-promoting effects. While the integration of chemical, microbiological, and computational approaches offers valuable preliminary insights, the study is limited by its in vitro design and optical-density-based readouts. Future investigations incorporating metabolite profiling, fermentation end-product analysis, and in vivo validation will be essential to confirm the functional significance of the observed growth-stimulation effects.

## Figures and Tables

**Table 1:** Fatty-acid composition of Romanian hemp seed oils expressed as percentage of total identified FAMEs (%). Compounds identified by GC-MS based on NIST 2017 mass spectral matches and verified using calculated retention indices (RI) relative to the Supelco 37-component FAME Mix (RIref).

Fatty Acid as Methyl Ester Percentage of Total Compounds (%)	RI c	RIref	THSO	SHSO	AHSO
Palmitoleic acid (C16:1, ω-7)	1870	1869	0.063 ± 0.008 ^a^	0.073 ± 0.015 ^a^	0.089 ± 0.010 ^a^
Palmitic acid (C16:0)	1920	1921	7.051 ± 0.109 ^b^	6.531 ± 0.078 ^a^	7.895 ± 0.163 ^c^
Heptadecanoic acid (C17:0)	2024	2026	0.024 ± 0.006 ^a^	0.031 ± 0.017 ^a^	0.026 ± 0.010 ^a^
Linoleic acid (C18:2, ω-6)	2093	2090	51.512 ± 0.252 ^b^	51.858 ± 0.106 ^b^	49.424 ± 0.112 ^a^
α-Linolenic acid (C18:3, ω-3)	2094	2091	9.852 ± 0.093 ^a^	14.365 ± 0.125 ^c^	12.600 ± 0.082 ^b^
γ-Linolenic acid (C18:3, ω-6)	2105	2110	2.463 ± 0.087 ^a^	3.591 ± 0.105 ^c^	3.150 ± 0.180 ^b^
Oleic acid (C18:1, ω-9)	2117	2118	22.780 ± 0.203 ^c^	16.343 ± 0.116 ^a^	18.697 ± 0.126 ^b^
Vaccenic acid (C18:1, ω-7)	2120	2119	1.075 ± 0.028 ^a^	1.014 ± 0.043 ^a^	1.326 ± 0.094 ^b^
Stearic acid (C18:0)	2132	2130	3.194 ± 0.087 ^a^	3.468 ± 0.074 ^b^	3.575 ± 0.087 ^b^
Arachidonic acid (C20:4, ω-6)	2270	2274	0.511 ± 0.017 ^a^	0.938 ± 0.029 ^b^	1.082 ± 0.044 ^c^
11-Eicosenoic acid (C20:1, ω-9)	2354	2356	0.292 ± 0.047 ^a^	0.321 ± 0.029 ^a^	0.409 ± 0.078 ^a^
Arachidic acid (C20:0)	2429	2433	0.785 ± 0.031 ^a^	0.844 ± 0.033 ^a^	1.024 ± 0.012 ^b^
Behenic acid (C22:0)	2529	2531	0.206 ± 0.015 ^a^	0.205 ± 0.103 ^a^	0.283 ± 0.016 ^a^
Lignoceric acid (C24:0)	2761	2760	0.050 ± 0.015 ^a^	0.046 ± 0.030 ^a^	0.079 ± 0.030 ^a^
Unassigned C20 PUFA *	-	-	0.142 ± 0.015 ^a^	0.372 ± 0.019 ^b^	0.341 ± 0.018 ^b^
SFA			11.310 ± 0.231 ^a^	11.125 ± 0.128 ^a^	12.880 ± 0.090 ^b^
MUFA			24.210 ± 0.226 ^c^	17.752 ± 0.148 ^a^	20.520 ± 0.299 ^b^
PUFA			64.478 ± 0.303 ^a^	71.123 ± 0.160 ^c^	66.598 ± 0.106 ^b^
ω-3			9.852 ± 0.093 ^a^	14.365 ± 0.125 ^c^	12.600 ± 0.094 ^b^
ω-6			54.486 ± 0.263 ^b^	56.388 ± 0.061 ^c^	53.656 ± 0.037 ^a^

Hemp seed oil: THSO–Teodora; SHSO–Silvana; AHSO–Armanca; SFA–saturated fatty acids, MUFA–monounsaturated fatty acids; PUFA–polyunsaturated fatty acids. * This compound was excluded from ω-3 total and ratio calculations. Results are presented as the mean of three replicates ± standard deviation (SD) (*n* = 3). Different lowercase letters (a–c) indicate statistically significant differences (*p* < 0.05) among samples within the same row, as determined by ANOVA. Means that sharing a letter is not significantly different. C16:1: AHSO vs. SHSO: *p* = 0.291; AHSO vs. THSO: *p* = 0.067; SHSO vs. THSO: *p* = 0.516; C16:0: AHSO vs. SHSO: *p* < 0.001; AHSO vs. THSO: *p* < 0.001; SHSO vs. THSO: *p* = 0.005; C17:0 AHSO vs. SHSO: *p* = 0.863; AHSO vs. THSO: *p* = 0.994; SHSO vs. THSO: *p* = 0.810; C20:4 AHSO vs. SHSO: *p* = 0.004; AHSO vs. THSO: *p* < 0.001; SHSO vs. THSO: *p* < 0.001; Unassigned C20 PUFA AHSO vs. SHSO: *p* = 0.156; AHSO vs. THSO: *p* < 0.001; SHSO vs. THSO: *p* < 0.001; C18:2 AHSO vs. SHSO: *p* < 0.001;AHSO vs. THSO: *p* < 0.001; SHSO vs. THSO: *p* = 0.103; C18:3 ω-3 all pairs < 0.001; C18:3, ω-6 AHSO vs. SHSO: *p* = 0.014;AHSO vs. THSO: *p* = 0.002; SHSO vs. THSO: *p* < 0.001; C18:1, ω-9: all pairs < 0.001; C18:1, ω-7: AHSO vs. SHSO: *p* = 0.002; AHSO vs. THSO: *p* = 0.006; SHSO vs. THSO: *p* = 0.497; C18:0 AHSO vs. SHSO: *p* = 0.325; AHSO vs. THSO: *p* = 0.003; SHSO vs. THSO: *p* = 0.015; C20:1 AHSO vs. SHSO: *p* = 0.202; AHSO vs. THSO: *p* = 0.090; SHSO vs. THSO: *p* = 0.805; C20:0 AHSO vs. SHSO: *p* < 0.001;AHSO vs. THSO: *p* < 0.001; SHSO vs. THSO: *p* = 0.083; C22:0 AHSO vs. SHSO: *p* = 0.327; AHSO vs. THSO: *p* = 0.338; SHSO vs. THSO: *p* = 1.000; C24:0 AHSO vs. SHSO: *p* = 0.328; AHSO vs. THSO: *p* = 0.395; SHSO vs. THSO: *p* = 0.986; SFA:AHSO vs. SHSO: *p* < 0.001;AHSO vs. THSO: *p <* 0.001; SHSO vs. THSO: *p* = 0.395; MUFA: all pairs < 0.001; PUFA: all pairs < 0.001; ω-3: all pairs < 0.001; ω-6: AHSO vs. SHSO: *p* < 0.001; AHSO vs. THSO: *p* = 0.002; SHSO vs. THSO: *p* < 0.001.

**Table 2:** Mean ± SD values of %Effect for each probiotic strain (*L. rhamnosus*, *L. paracasei*, and *L. acidophilus*) exposed to the three hemp seed oils (THSO, SHSO, AHSO).

	*Lacticaseibacillus rhamnosus* (GG; HN001)			*Lactobacillus paracasei subsp. paracasei* ATCC BAA-52			*Lactobacillus acidophilus* ATCC 4356		
mg/mL	THSO	SHSO	AHSO	THSO	SHSO	AHSO	THSO	SHSO	AHSO
	% probiotic growth-promoting effect								
0.3	−43.81 ±0.017 ^b^	−51.32 ±0.026 ^a^	−26.24 ±0.012 ^c^	−49.62 ±0.017 ^a^	−23.66 ±0.012 ^c^	−30.89 ±0.022 ^b^	−4.38 ±0.017 ^e^	−11.68 ±0.027 ^a^	−10.95 ±0.024 ^b^
0.6	−13.97 ±0.023 ^f^	−17.35 ±0.019 ^d^	−15.03 ±0.028 ^e^	−14.46 ±0.028 ^d^	−12.05 ±0.015 ^e^	−10.62 ±0.023 ^g^	1.46 ±0.021 ^i^	−0.73 ±0.011 ^g^	−5.84 ±0.025 ^c^
1.3	−12.70 ±0.014 ^j^	−13.65 ±0.021 ^h^	−13.86 ±0.015 ^g^	−10.62 ±0.011 ^g^	−10.84 ±0.019 ^f^	−9.64 ±0.025 ^i^	6.57 ±0.018 ^l^	2.19 ±0.012 ^j^	−5.11 ±0.013 ^d^
2.5	−10.90 ±0.029 ^m^	−12.91 ±0.011 ^i^	−12.49 ±0.024 ^k^	−9.86 ±0.013 ^h^	−9.42 ±0.027 ^j^	−9.31 ±0.021 ^k^	8.03 ±0.016 ^m^	2.19 ±0.014 ^j^	−3.65 ±0.015 ^f^
5	−7.94 ±0.018 ^n^	−6.67 ±0.027 ^p^	−12.06 ±0.013 ^l^	−3.40 ±0.014 ^r^	−8.98 ±0.029 ^n^	−9.20 ±0.018 ^l^	13.87 ±0.013 ^p^	2.92 ±0.029 ^k^	0.73 ±0.014 ^h^
9	0.42 ±0.022 ^r^	−4.76 ±0.016 ^q^	−10.90 ±0.025 ^m^	−1.42 ±0.024 ^s^	−7.78 ±0.016 ^p^	−9.09 ±0.026 ^m^	13.87 ±0.022 ^p^	9.49 ±0.023 ^n^	1.46 ±0.015 ^i^
16	2.43 ±0.020 ^s^	16.83 ±0.030 ^t^	−6.88 ±0.023 ^o^	20.92 ±0.020 ^t^	−3.61 ±0.015 ^q^	−8.32 ±0.023 ^o^	54.74 ±0.012 ^q^	10.95 ±0.020 ^o^	2.92 ±0.019 ^k^

Values represent mean ± standard deviation (SD) from three replicates (*n* = 3). Different lowercase letters (a–t) within the same strain indicate statistically significant differences among concentrations according to two-way ANOVA followed by Tukey’s post hoc test (*p* < 0.05). Abbreviations: THSO–Teodora hemp seed oil; SHSO–Silvana hemp seed oil; AHSO–Armanca hemp seed oil.

**Table 3:** Mean ± SD values of the Potentiation Coefficient (CP).

mg/mL	*Lacticaseibacillus rhamnosus* (GG; HN001)			*Lactobacillus paracasei subsp.paracasei* ATCC BAA-52			*Lactobacillus acidophilus* ATCC 4356		
	THSO	SHSO	AHSO	THSO	SHSO	AHSO	THSO	SHSO	AHSO
0.3	0.56 ±0.022 ^b^	0.49 ±0.015 ^a^	0.74 ±0.028 ^c^	0.50 ±0.016 ^a^	0.76 ±0.023 ^c^	0.69 ±0.012 ^b^	0.96 ±0.019 ^cde^	0.88 ±0.013 ^a^	0.89 ±0.027 ^ab^
0.6	0.86 ±0.018 ^de^	0.83 ±0.013 ^d^	0.85 ±0.027 ^d^	0.86 ±0.028 ^d^	0.88 ±0.019 ^d^	0.89 ±0.014 ^d^	1.01 ±0.027 ^defg^	0.99 ±0.012 ^cdef^	0.94 ±0.015 ^abc^
1.3	0.87 ±0.019 ^def^	0.86 ±0.024 ^de^	0.86 ±0.012 ^de^	0.89 ±0.025 ^d^	0.89 ±0.021 ^d^	0.90 ±0.017 ^de^	1.07 ±0.024 ^ghij^	1.02 ±0.018 ^efgh^	0.95 ±0.023 ^bcd^
2.5	0.89 ±0.029 ^defg^	0.87 ±0.014 ^def^	0.88 ±0.017 ^def^	0.90 ±0.027 ^de^	0.91 ±0.013 ^def^	0.91 ±0.024 ^def^	1.08 ±0.014 ^hijk^	1.02 ±0.029 ^efgh^	0.96 ±0.016 ^dce^
5	0.92 ±0.021 ^efg^	0.93 ±0.016 ^fg^	0.88 ±0.025 ^def^	0.97 ±0.018 ^fg^	0.91 ±0.022 ^def^	0.91 ±0.015 ^def^	1.14 ±0.022 ^k^	1.03 ±0.017 ^fghi^	1.01 ±0.028 ^defg^
9	1.00 ±0.011 ^hi^	0.95 ±0.023 ^gh^	0.89 ±0.026 ^defg^	0.99 ±0.026 ^g^	0.92 ±0.020 ^def^	0.91 ±0.029 ^def^	1.14 ±0.011 ^k^	1.09 ±0.025 ^ijk^	1.01 ±0.026 ^defg^
16	1.02 ±0.020 ^i^	1.17 ±0.022 ^j^	0.93 ±0.015 ^fg^	1.21 ±0.011 ^h^	0.96 ±0.018 ^efg^	0.92 ±0.024 ^def^	1.55 ±0.020 ^l^	1.11 ±0.027 ^jk^	1.03 ±0.015 ^fgji^

Values represent mean ± standard deviation (SD) from three replicates (*n* = 3). Different lowercase letters (a–l) within the same strain indicate statistically significant differences among concentrations according to two-way ANOVA followed by Tukey’s post hoc test (*p* < 0.05). Abbreviations: THSO–Teodora hemp seed oil; SHSO–Silvana hemp seed oil; AHSO–Armanca hemp seed oil; CP–Potentiation Coefficient.

**Table 4:** Docking Results of Hydratase (4IA6), Enolase (4MKS), Glyceraldehyde-3-phosphate dehydrogenase (5J9G), Ribonucleoside hydrolase (8QND) with seven (7) fatty acid ligands of high abundance.

S/No	Ligands (Fatty Acids)	Binding Energy (kcal/mol)				Amino Acid Residues Involved in Binding Interaction			
		4IA6	4MKS	5J9G	8QND	4IA6	4MKS	5J9G	8QND
1	Palmitic acid C16:0	−5.1	−4.5	−4.3	−4.4	VAL B:77, ARG B:78, PHE B:398, VAL B:407, VAL B:461, VAL B:463	ARG B:34, ILE B:36, ARG B:408	ARG A:12, ILE A:13, SER A:125, PRO A:127, CYS A:156, PHE A:324	ASN D:162, PHE D:169
2	Linoleic acid C18:2	−7.8	−5.5	−4.8	−5.1	MET A:185, ALA A:187, PHE A:214, LEU A:217, PHE A:219, TRP A:343, ILE A:378, GLU A:387, TYR A:411	ARG B:88, PHE B:350, ILE B:353, LYS B:357	ARG A:12, ILE A:13, CYS A:156, HIS A:183, ARG A:239	ILE C:19, PHE C:169
3	α-Linolenic acid C18:3	−7.4	−5.5	−5.6	−5.3	MET B:185, ALA B:187, PHE B:214, LEU B:217, PHE B:219, TRP B:343, ILE B:378, GLU B:387, HIS B:393, TYR B:411, LEU B:413	ARG B:88, TYR B: 133, LEU B:134, PHE B:350	ASP A:38, LEU A:39, THR A:40, PRO A:82, PHE A:104	ILE D:19, PHE D:169, TYR D:226
4	γ-Linolenic acid C18:3	-7.4	-5.5	-5.0	-5.4	MET B:185, ALA B:187, PHE B:214, LEU B:217, PHE B:219, TRP B:343, ILE B:378, GLU B:387, SER B:389, HIS B:393, TYR B:411	TYR A:133, LEU A:134, PHE A:350, ILE A:353, ALA A:383	ASN A:37, ASN A:8, LEU A:39, GLU A:81, PRO A:82, PHE A:104	ILE C:19, ASN C:45, HIS D:237
5	Oleic acid C18:1	−5.2	−4.8	−4.1	−5.1	VAL B:77, ARG B:78, THR B:350 , PHE B:398, GLN B:405 , VAL B:461, VAL B:463,	ILE B:36, ARG A:120, THR A:375	ARG B:12, ILE B:13, SER B:125, CYS B:156	ASP D:20, PHE D:169, HIS D:237
6	Vaccenic acid C18:1	−5.3	−5.2	−5.1	−5.1	TYR A:3, VAL A:533	ARG A:88, TYR A:133, PHE A:350	GLY A:11, ASP B:38, LEU B:39, THR B:40, PHE B:104, TYR B:105	ILE A:19, GLU A:168, PHE A:169, ASN A:170, HIS A:237
7	Stearic acid C18:0	−6.2	−4.9	−4.9	−4.7	ARG B:81, ALA B:187, ILE B:292, TRP B:343 , PHE B:507	ARG B:34, ILE B:36, ASP A:374	ASN A:37, ASP A:38 , LEU A:39, PRO A:82, PHE A:104	ILE D:19, ASP D:20, ARG D:234, PHE D:169, HIS D:237

Pink color: Alkyl/p-Alkyl bonds, green color: Hydrogen bonds, faint-green: carbon-hydrogen bonds, red color: unfavorable bonds, purple color: sigma/pi-sigma bonds.

## Data Availability

The analytical datasets supporting this study are archived within the Interdisciplinary Research Platform of the University of Life Sciences “King Michael I” of Timișoara (USVT), which issues official analytical bulletins for authorized determinations. To ensure transparency, summarized datasets and statistical outputs are provided as Supplementary File S1–S3. Additional raw data can be obtained from the corresponding author upon reasonable request.

## Supplementary Materials

The following supporting information can be downloaded at: https://www.mdpi.com/article/10.3390/plants14223465/s1. Supplementary File S1: Chemical composition, pigment content, total phenolics, antioxidant activity, and statistical analysis. Supplementary File S2: In-vitro probiotic data and statistical analysis. Supplementary File S3: FAME area tables, TPC DPPH replicate values, and raw per-well OD.
