# Supplementary material for: Chemical Profile, Bioactive Constituents and In Vitro Growth Stimulation Properties of Cold-Pressed Hemp Seed Oils from Romanian Varieties: In Vitro and In Silico Evaluation
Source: Plants (Basel). 2025 Nov 13;14(22):3465. doi: 10.3390/plants14223465 (PMC12655998; doi:10.3390/plants14223465)
Supplement: Supplementary file 1 [file plants-14-03465-s001.zip › Supplementary file S1_Chemical composition, pigment content, total phenolics, antioxidant activity, and statistical analysis.pdf]

Supplementary file S1

Statistical correlation between total phenolics and antioxidant activity

Pearson's Correlations

Variable		TPC	DPPH
1. TPC	Pearson's r	—	
	p-value	—	
	Lower 95% CI	—	
	Upper 95% CI	—	
2. DPPH	Pearson's r	0.896**	—
	p-value	0.001	—
	Lower 95% CI	0.572	—
	Upper 95% CI	0.978	—

* p < .05, ** p < .01, *** p < .001

ANOVA

ANOVA - C16:1

Homogeneity Correction	Cases	Sum of Squares	df	Mean Square	F	p
Welch	Sample	0.001	2.000	5.124×10^{-4}	5.876	0.069
	Residuals	7.600×10^{-4}	3.766	2.018×10^{-4}		

Note. Type III Sum of Squares

Descriptives

Descriptives - C16:1

Sample	N	Mean	SD	SE	Coefficient of variation
AHSO	3	0.089	0.010	0.005	0.107
SHSO	3	0.073	0.015	0.009	0.208
THSO	3	0.063	0.008	0.004	0.120

Post Hoc Tests

Standard (HSD)

Post Hoc Comparisons - Sample

		Mean Difference	95% CI for Mean Difference Lower	Upper	SE	df	t	p_{tukey}
AHSO	SHSO	0.015	-0.013	0.044	0.009	6	1.669	0.291
	THSO	0.026	-0.002	0.054	0.009	6	2.829	0.067
SHSO	THSO	0.011	-0.018	0.039	0.009	6	1.161	0.516

Note. P-value and confidence intervals adjusted for comparing a family of 3 estimates (confidence intervals corrected using the tukey method).

Letter-Based Grouping - Sample

Sample	Letter
AHSO	a
SHSO	a
THSO	a

Note. If two or more means share the same grouping symbol, then we cannot show them to be different, but we also did not show them to be the same.

ANOVA

ANOVA - C16:0

Homogeneity Correction	Cases	Sum of Squares	df	Mean Square	F	p
Welch	Sample	2.841	2.000	1.420	77.835	< .001
	Residuals	0.089	3.725	0.024		

Note. Type III Sum of Squares

Descriptives

Descriptives - C16:0

Sample	N	Mean	SD	SE	Coefficient of variation
AHSO	3	7.895	0.163	0.094	0.021
SHSO	3	6.531	0.078	0.045	0.012
THSO	3	7.051	0.109	0.063	0.015

Post Hoc Tests

Standard (HSD)

Post Hoc Comparisons - Sample

		Mean Difference	95% CI for Mean Difference Lower	95% CI for Mean Difference Upper	SE	df	t	p_{tukey}
AHSO	SHSO	1.363	1.058	1.668	0.099	6	13.717	< .001***
	THSO	0.844	0.539	1.149	0.099	6	8.489	< .001***
SHSO	THSO	-0.520	-0.825	-0.215	0.099	6	-5.229	0.005**

** p < .01, *** p < .001

Note. P-value and confidence intervals adjusted for comparing a family of 3 estimates (confidence intervals corrected using the tukey method).

Letter-Based Grouping - Sample

Sample	Letter
AHSO	c
SHSO	a
THSO	b

Note. If two or more means share the same grouping symbol, then we cannot show them to be different, but we also did not show them to be the same.

ANOVA

ANOVA - C17:0

Homogeneity Correction	Cases	Sum of Squares	df	Mean Square	F	p
Welch	Sample	6.200×10^{-5}	2.000	3.100×10^{-5}	0.148	0.868
	Residuals	8.180×10^{-4}	3.520	2.324×10^{-4}		

Note. Type III Sum of Squares

Descriptives

Descriptives - C17:0

Sample	N	Mean	SD	SE	Coefficient of variation
AHSO	3	0.025	0.010	0.006	0.400
SHSO	3	0.030	0.017	0.010	0.551
THSO	3	0.024	0.006	0.003	0.250

Post Hoc Tests

Standard (HSD)

Post Hoc Comparisons - Sample

		Mean Difference	95% CI for Mean Difference Lower	Upper	SE	df	t	p_{tukey}
AHSO	SHSO	-0.005	-0.034	0.024	0.010	6	-0.524	0.863
	THSO	0.001	-0.028	0.030	0.010	6	0.105	0.994
SHSO	THSO	0.006	-0.023	0.035	0.010	6	0.629	0.810

Note. P-value and confidence intervals adjusted for comparing a family of 3 estimates (confidence intervals corrected using the tukey method).

Letter-Based Grouping - Sample

Sample	Letter
AHSO	a
SHSO	a
THSO	a

Note. If two or more means share the same grouping symbol, then we cannot show them to be different, but we also did not show them to be the same.

ANOVA

ANOVA - unassigned C20 PUFA

Homogeneity Correction	Cases	Sum of Squares	df	Mean Square	F	p
Welch	Sample	0.095	2.000	0.047	156.593	< .001
	Residuals	0.002	3.937	4.575×10^{-4}		

Note. Type III Sum of Squares

Descriptives

Descriptives - unassigned C20 PUFA

Sample	N	Mean	SD	SE	Coefficient of variation
AHSO	3	0.341	0.018	0.010	0.052
SHSO	3	0.372	0.019	0.011	0.052
THSO	3	0.140	0.015	0.008	0.103

Post Hoc Tests

Standard (HSD)

Post Hoc Comparisons - Sample

		Mean Difference	95% CI for Mean Difference		SE	df	t	p_{tukey}
			Lower	Upper				
AHSO	SHSO	-0.031	-0.074	0.013	0.014	6	-2.168	0.156
	THSO	0.201	0.157	0.244	0.014	6	14.184	< .001***
SHSO	THSO	0.231	0.188	0.275	0.014	6	16.352	< .001***

*** p < .001

Note. P-value and confidence intervals adjusted for comparing a family of 3 estimates (confidence intervals corrected using the tukey method).

Letter-Based Grouping - Sample

Sample	Letter
AHSO	b
SHSO	b
THSO	a

Note. If two or more means share the same grouping symbol, then we cannot show them to be different, but we also did not show them to be the same.

ANOVA

ANOVA - C20:4

Homogeneity Correction	Cases	Sum of Squares	df	Mean Square	F	p
Welch	Sample	0.530	2.000	0.265	330.062	< .001
	Residuals	0.006	3.511	0.002		

Note. Type III Sum of Squares

Descriptives

Descriptives - C20:4

Sample	N	Mean	SD	SE	Coefficient of variation
AHSO	3	1.082	0.044	0.025	0.041
SHSO	3	0.938	0.029	0.017	0.031
THSO	3	0.511	0.017	0.010	0.033

Post Hoc Tests

Standard (HSD)

Post Hoc Comparisons - Sample

		Mean Difference	95% CI for Mean Difference		SE	df	t	p_{tukey}
			Lower	Upper				
AHSO	SHSO	0.144	0.064	0.224	0.026	6	5.533	0.004**
	THSO	0.571	0.491	0.651	0.026	6	21.904	< .001***
SHSO	THSO	0.427	0.347	0.507	0.026	6	16.370	< .001***

** p < .01, *** p < .001

Note. P-value and confidence intervals adjusted for comparing a family of 3 estimates (confidence intervals corrected using the tukey method).

Letter-Based Grouping - Sample

Sample	Letter
AHSO	c
SHSO	b
THSO	a

Note. If two or more means share the same grouping symbol, then we cannot show them to be different, but we also did not show them to be the same.

ANOVA

ANOVA - C18:2

Homogeneity Correction	Cases	Sum of Squares	df	Mean Square	F	p
Welch	Sample	10.401	2.000	5.201	327.574	< .001
	Residuals	0.175	3.738	0.047		

Note. Type III Sum of Squares

Descriptives

Descriptives - C18:2

Sample	N	Mean	SD	SE	Coefficient of variation
AHSO	3	49.424	0.112	0.065	0.002
SHSO	3	51.858	0.106	0.061	0.002
THSO	3	51.512	0.252	0.145	0.005

Post Hoc Tests

Standard (HSD)

Post Hoc Comparisons - Sample

		Mean Difference	95% CI for Mean Difference		SE	df	t	p_{tukey}
			Lower	Upper				
AHSO	SHSO	-2.434	-2.861	-2.007	0.139	6	-17.477	< .001***
	THSO	-2.087	-2.515	-1.660	0.139	6	-14.988	< .001***
SHSO	THSO	0.347	-0.081	0.774	0.139	6	2.489	0.103

*** p < .001

Note. P-value and confidence intervals adjusted for comparing a family of 3 estimates (confidence intervals corrected using the tukey method).

Letter-Based Grouping - Sample

Sample	Letter
AHSO	a
SHSO	b
THSO	b

Note. If two or more means share the same grouping symbol, then we cannot show them to be different, but we also did not show them to be the same.

ANOVA

ANOVA - C18:3 alpha

Homogeneity Correction	Cases	Sum of Squares	df	Mean Square	F	p
Welch	Sample	31.035	2.000	15.518	1218.275	< .001
	Residuals	0.062	3.899	0.016		

Note. Type III Sum of Squares

Descriptives

Descriptives - C18:3 alpha

Sample	N	Mean	SD	SE	Coefficient of variation
AHSO	3	12.600	0.082	0.047	0.006
SHSO	3	14.365	0.125	0.072	0.009
THSO	3	9.852	0.093	0.053	0.009

Post Hoc Tests

Standard (HSD)

Post Hoc Comparisons - Sample

		Mean Difference	95% CI for Mean Difference Lower	95% CI for Mean Difference Upper	SE	df	t	p_{tukey}
AHSO	SHSO	-1.764	-2.018	-1.510	0.083	6	-21.315	< .001***
	THSO	2.749	2.495	3.003	0.083	6	33.207	< .001***
SHSO	THSO	4.513	4.259	4.767	0.083	6	54.522	< .001***

*** p < .001

Note. P-value and confidence intervals adjusted for comparing a family of 3 estimates (confidence intervals corrected using the tukey method).

Letter-Based Grouping - Sample

Sample	Letter
AHSO	b
SHSO	c
THSO	a

Note. If two or more means share the same grouping symbol, then we cannot show them to be different, but we also did not show them to be the same.

ANOVA

ANOVA - C18:3 gamma

Homogeneity Correction	Cases	Sum of Squares	df	Mean Square	F	p
Welch	Sample	1.937	2.000	0.968	89.826	< .001
	Residuals	0.102	3.770	0.027		

Note. Type III Sum of Squares

Descriptives

Descriptives - C18:3 gamma

Sample	N	Mean	SD	SE	Coefficient of variation
AHSO	3	3.150	0.180	0.104	0.057
SHSO	3	3.591	0.105	0.060	0.029
THSO	3	2.463	0.087	0.050	0.035

Post Hoc Tests

Standard (HSD)

Post Hoc Comparisons - Sample

		Mean Difference	95% CI for Mean Difference Lower	95% CI for Mean Difference Upper	SE	df	t	p_{tukey}
AHSO	SHSO	-0.441	-0.767	-0.115	0.106	6	-4.147	0.014*
	THSO	0.687	0.361	1.013	0.106	6	6.463	0.002**
SHSO	THSO	1.127	0.801	1.453	0.106	6	10.610	< .001***

* p < .05, ** p < .01, *** p < .001

Note. P-value and confidence intervals adjusted for comparing a family of 3 estimates (confidence intervals corrected using the tukey method).

Letter-Based Grouping - Sample

Sample	Letter
AHSO	b
SHSO	c
THSO	a

Note. If two or more means share the same grouping symbol, then we cannot show them to be different, but we also did not show them to be the same.

Results

ANOVA

ANOVA - C18:1omega9

Homogeneity Correction	Cases	Sum of Squares	df	Mean Square	F	p
Welch	Sample	63.641	2.000	31.821	998.859	< .001
	Residuals	0.141	3.847	0.037		

Note. Type III Sum of Squares

Descriptives

Descriptives - C18:1omega9

Sample	N	Mean	SD	SE	Coefficient of variation
AHSO	3	18.697	0.126	0.073	0.007
SHSO	3	16.343	0.116	0.067	0.007
THSO	3	22.780	0.203	0.117	0.009

Post Hoc Tests

Standard (HSD)

Post Hoc Comparisons - Sample

		Mean Difference	95% CI for Mean Difference Lower	95% CI for Mean Difference Upper	SE	df	t	p_{tukey}
AHSO	SHSO	2.354	1.970	2.737	0.125	6	18.817	< .001***
	THSO	-4.083	-4.467	-3.699	0.125	6	-32.643	< .001***
SHSO	THSO	-6.437	-6.820	-6.053	0.125	6	-51.460	< .001***

*** p < .001

Note. P-value and confidence intervals adjusted for comparing a family of 3 estimates (confidence intervals corrected using the tukey method).

Letter-Based Grouping - Sample

Sample	Letter
AHSO	b
SHSO	a
THSO	c

Note. If two or more means share the same grouping symbol, then we cannot show them to be different, but we also did not show them to be the same.

ANOVA

ANOVA - C18:1omega7

Homogeneity Correction	Cases	Sum of Squares	df	Mean Square	F	p
Welch	Sample	0.164	2.000	0.082	11.471	0.029
	Residuals	0.023	3.506	0.007		

Note. Type III Sum of Squares

Descriptives

Descriptives - C18:1omega7

Sample	N	Mean	SD	SE	Coefficient of variation
AHSO	3	1.326	0.094	0.054	0.071
SHSO	3	1.014	0.043	0.025	0.042
THSO	3	1.075	0.028	0.016	0.026

Post Hoc Tests

Standard (HSD)

Post Hoc Comparisons - Sample

		Mean Difference	95% CI for Mean Difference Lower	95% CI for Mean Difference Upper	SE	df	t	p_{tukey}
AHSO	SHSO	0.311	0.157	0.466	0.050	6	6.174	0.002**
	THSO	0.251	0.096	0.406	0.050	6	4.977	0.006**
SHSO	THSO	-0.060	-0.215	0.094	0.050	6	-1.196	0.497

** p < .01

Note. P-value and confidence intervals adjusted for comparing a family of 3 estimates (confidence intervals corrected using the tukey method).

Letter-Based Grouping - Sample

Sample	Letter
AHSO	b
SHSO	a
THSO	a

Note. If two or more means share the same grouping symbol, then we cannot show them to be different, but we also did not show them to be the same.

Results

ANOVA

ANOVA - C18:0

Homogeneity Correction	Cases	Sum of Squares	df	Mean Square	F	p
Welch	Sample	0.232	2.000	0.116	13.443	0.017
	Residuals	0.041	3.977	0.010		

Note. Type III Sum of Squares

Descriptives

Descriptives - C18:0

Sample	N	Mean	SD	SE	Coefficient of variation
AHSO	3	3.575	0.087	0.050	0.024
SHSO	3	3.468	0.074	0.043	0.021
THSO	3	3.194	0.087	0.050	0.027

Post Hoc Tests

Standard (HSD)

Post Hoc Comparisons - Sample

		Mean Difference	95% CI for Mean Difference		SE	df	t	p_{tukey}
			Lower	Upper				
AHSO	SHSO	0.106	-0.101	0.313	0.067	6	1.576	0.325
	THSO	0.381	0.174	0.588	0.067	6	5.648	0.003**
SHSO	THSO	0.275	0.068	0.482	0.067	6	4.072	0.015*

* p < .05, ** p < .01

Note. P-value and confidence intervals adjusted for comparing a family of 3 estimates (confidence intervals corrected using the tukey method).

Letter-Based Grouping - Sample

Sample	Letter
AHSO	b
SHSO	b
THSO	a

Note. If two or more means share the same grouping symbol, then we cannot show them to be different, but we also did not show them to be the same.

ANOVA

ANOVA - C 20:1

Homogeneity Correction	Cases	Sum of Squares	df	Mean Square	F	p
Welch	Sample	0.022	2.000	0.011	2.096	0.251
	Residuals	0.018	3.548	0.005		

Note. Type III Sum of Squares

Descriptives

Descriptives - C 20:1

Sample	N	Mean	SD	SE	Coefficient of variation
AHSO	3	0.409	0.078	0.045	0.190
SHSO	3	0.321	0.029	0.017	0.090
THSO	3	0.292	0.047	0.027	0.160

Post Hoc Tests

Standard (HSD)

Post Hoc Comparisons - Sample

		Mean Difference	95% CI for Mean Difference Lower	95% CI for Mean Difference Upper	SE	df	t	p_{tukey}
AHSO	SHSO	0.088	-0.050	0.226	0.045	6	1.962	0.202
	THSO	0.117	-0.021	0.254	0.045	6	2.601	0.090
SHSO	THSO	0.029	-0.109	0.166	0.045	6	0.639	0.805

Note. P-value and confidence intervals adjusted for comparing a family of 3 estimates (confidence intervals corrected using the tukey method).

Letter-Based Grouping - Sample

Sample	Letter
AHSO	a
SHSO	a
THSO	a

Note. If two or more means share the same grouping symbol, then we cannot show them to be different, but we also did not show them to be the same.

ANOVA

ANOVA - C20:0

Homogeneity Correction	Cases	Sum of Squares	df	Mean Square	F	p
Welch	Sample	0.093	2.000	0.046	87.782	0.002
	Residuals	0.004	3.227	0.001		

Note. Type III Sum of Squares

Descriptives

Descriptives - C20:0

Sample	N	Mean	SD	SE	Coefficient of variation
AHSO	3	1.024	0.012	0.007	0.011
SHSO	3	0.844	0.033	0.019	0.039
THSO	3	0.785	0.031	0.018	0.040

Post Hoc Tests

Standard (HSD)

Post Hoc Comparisons - Sample

		Mean Difference	95% CI for Mean Difference Lower	95% CI for Mean Difference Upper	SE	df	t	p_{tukey}
AHSO	SHSO	0.180	0.112	0.248	0.022	6	8.164	< .001***
	THSO	0.239	0.171	0.306	0.022	6	10.824	< .001***
SHSO	THSO	0.059	-0.009	0.126	0.022	6	2.661	0.083

*** p < .001

Note. P-value and confidence intervals adjusted for comparing a family of 3 estimates (confidence intervals corrected using the tukey method).

Letter-Based Grouping - Sample

Sample	Letter
AHSO	b
SHSO	a
THSO	a

Note. If two or more means share the same grouping symbol, then we cannot show them to be different, but we also did not show them to be the same.

ANOVA

ANOVA - C22:0

Homogeneity Correction	Cases	Sum of Squares	df	Mean Square	F	p
Welch	Sample	0.012	2.000	0.006	15.799	0.017
	Residuals	0.022	3.581	0.006		

Note. Type III Sum of Squares

Descriptives

Descriptives - C22:0

Sample	N	Mean	SD	SE	Coefficient of variation
AHSO	3	0.283	0.016	0.009	0.055
SHSO	3	0.205	0.103	0.059	0.500
THSO	3	0.206	0.015	0.009	0.074

Post Hoc Tests

Standard (HSD)

Post Hoc Comparisons - Sample

		Mean Difference	95% CI for Mean Difference		SE	df	t	p_{tukey}
			Lower	Upper				
AHSO	SHSO	0.078	-0.074	0.229	0.049	6	1.571	0.327
	THSO	0.076	-0.075	0.228	0.049	6	1.544	0.338
SHSO	THSO	-0.001	-0.153	0.150	0.049	6	-0.027	1.000

Note. P-value and confidence intervals adjusted for comparing a family of 3 estimates (confidence intervals corrected using the tukey method).

Letter-Based Grouping - Sample

Sample	Letter
AHSO	a
SHSO	a
THSO	a

Note. If two or more means share the same grouping symbol, then we cannot show them to be different, but we also did not show them to be the same.

Results

ANOVA

ANOVA - C24:0

Homogeneity Correction	Cases	Sum of Squares	df	Mean Square	F	p
Welch	Sample	0.002	2.000	9.693×10^{-4}	1.101	0.425
	Residuals	0.004	3.537	0.001		

Note. Type III Sum of Squares

Descriptives

Descriptives - C24:0

Sample	N	Mean	SD	SE	Coefficient of variation
AHSO	3	0.079	0.030	0.017	0.374
SHSO	3	0.046	0.030	0.017	0.637
THSO	3	0.050	0.015	0.008	0.292

Post Hoc Tests

Standard (HSD)

Post Hoc Comparisons - Sample

		Mean Difference	95% CI for Mean Difference Lower	95% CI for Mean Difference Upper	SE	df	t	p_{tukey}
AHSO	SHSO	0.033	-0.031	0.097	0.021	6	1.568	0.328
	THSO	0.029	-0.035	0.093	0.021	6	1.408	0.395
SHSO	THSO	-0.003	-0.067	0.061	0.021	6	-0.160	0.986

Note. P-value and confidence intervals adjusted for comparing a family of 3 estimates (confidence intervals corrected using the tukey method).

Letter-Based Grouping - Sample

Sample	Letter
AHSO	a
SHSO	a
THSO	a

Note. If two or more means share the same grouping symbol, then we cannot show them to be different, but we also did not show them to be the same.

ANOVA

ANOVA - SFA

Homogeneity Correction	Cases	Sum of Squares	df	Mean Square	F	p
Welch	Sample	5.579	2.000	2.790	180.324	< .001
	Residuals	0.155	3.640	0.043		

Note. Type III Sum of Squares

Descriptives

Descriptives - SFA

Sample	N	Mean	SD	SE	Coefficient of variation
AHSO	3	12.880	0.090	0.052	0.007
SHSO	3	11.125	0.128	0.074	0.011
THSO	3	11.310	0.231	0.133	0.020

Post Hoc Tests

Standard (HSD)

Post Hoc Comparisons - Sample

		Mean Difference	95% CI for Mean Difference		SE	df	t	p_{tukey}
			Lower	Upper				
AHSO	SHSO	1.755	1.352	2.158	0.131	6	13.359	< .001***
	THSO	1.570	1.167	1.973	0.131	6	11.951	< .001***
SHSO	THSO	-0.185	-0.588	0.218	0.131	6	-1.408	0.395

*** p < .001

Note. P-value and confidence intervals adjusted for comparing a family of 3 estimates (confidence intervals corrected using the tukey method).

Letter-Based Grouping - Sample

Sample	Letter
AHSO	b
SHSO	a
THSO	a

Note. If two or more means share the same grouping symbol, then we cannot show them to be different, but we also did not show them to be the same.

ANOVA

ANOVA - MUFA

Homogeneity Correction	Cases	Sum of Squares	df	Mean Square	F	p
Welch	Sample	62.976	2.000	31.488	735.296	< .001
	Residuals	0.324	3.695	0.088		

Note. Type III Sum of Squares

Descriptives

Descriptives - MUFA

Sample	N	Mean	SD	SE	Coefficient of variation
AHSO	3	20.520	0.299	0.172	0.015
SHSO	3	17.752	0.148	0.085	0.008
THSO	3	24.210	0.226	0.130	0.009

Post Hoc Tests

Standard (HSD)

Post Hoc Comparisons - Sample

		Mean Difference	95% CI for Mean Difference Lower	95% CI for Mean Difference Upper	SE	df	t	p_{tukey}
AHSO	SHSO	2.768	2.186	3.351	0.190	6	14.588	< .001***
	THSO	-3.689	-4.272	-3.107	0.190	6	-19.441	< .001***
SHSO	THSO	-6.458	-7.040	-5.875	0.190	6	-34.028	< .001***

*** p < .001

Note. P-value and confidence intervals adjusted for comparing a family of 3 estimates (confidence intervals corrected using the tukey method).

Letter-Based Grouping - Sample

Sample	Letter
AHSO	b
SHSO	a
THSO	c

Note. If two or more means share the same grouping symbol, then we cannot show them to be different, but we also did not show them to be the same.

ANOVA

ANOVA - PUFA

Homogeneity Correction	Cases	Sum of Squares	df	Mean Square	F	p
Welch	Sample	69.133	2.000	34.567	857.649	< .001
	Residuals	0.257	3.571	0.072		

Note. Type III Sum of Squares

Descriptives

Descriptives - PUFA

Sample	N	Mean	SD	SE	Coefficient of variation
AHSO	3	66.598	0.106	0.061	0.002
SHSO	3	71.125	0.160	0.092	0.002
THSO	3	64.478	0.303	0.175	0.005

Post Hoc Tests

Standard (HSD)

Post Hoc Comparisons - Sample

		Mean Difference	95% CI for Mean Difference		SE	df	t	p_{tukey}
			Lower	Upper				
AHSO	SHSO	-4.525	-5.044	-4.007	0.169	6	-26.773	< .001***
	THSO	2.120	1.601	2.639	0.169	6	12.542	< .001***
SHSO	THSO	6.645	6.127	7.164	0.169	6	39.316	< .001***

*** p < .001

Note. P-value and confidence intervals adjusted for comparing a family of 3 estimates (confidence intervals corrected using the tukey method).

Letter-Based Grouping - Sample

Sample	Letter
AHSO	b
SHSO	c
THSO	a

Note. If two or more means share the same grouping symbol, then we cannot show them to be different, but we also did not show them to be the same.

ANOVA

ANOVA – omega 3

Homogeneity Correction	Cases	Sum of Squares	df	Mean Square	F	p
Welch	Sample	31.035	2.000	15.518	1199.187	< .001
	Residuals	0.066	3.940	0.017		

Note. Type III Sum of Squares

Descriptives

Descriptives - ώ3

Sample	N	Mean	SD	SE	Coefficient of variation
AHSO	3	12.600	0.094	0.054	0.007
SHSO	3	14.365	0.125	0.072	0.009
THSO	3	9.852	0.093	0.053	0.009

Post Hoc Tests

Standard (HSD)

Post Hoc Comparisons - Sample

		Mean Difference	95% CI for Mean Difference		SE	df	t	p_{tukey}
			Lower	Upper				
AHSO	SHSO	-1.764	-2.027	-1.502	0.086	6	-20.610	< .001***
	THSO	2.749	2.486	3.011	0.086	6	32.109	< .001***
SHSO	THSO	4.513	4.250	4.776	0.086	6	52.720	< .001***

*** p < .001

Note. P-value and confidence intervals adjusted for comparing a family of 3 estimates (confidence intervals corrected using the tukey method).

Letter-Based Grouping - Sample

Sample	Letter
AHSO	b
SHSO	c
THSO	a

Note. If two or more means share the same grouping symbol, then we cannot show them to be different, but we also did not show them to be the same.

ANOVA

ANOVA - omega 6

Homogeneity Correction	Cases	Sum of Squares	df	Mean Square	F	p
Welch	Sample	11.756	2.000	5.878	1834.839	< .001
	Residuals	0.149	3.360	0.044		

Note. Type III Sum of Squares

Descriptives

Descriptives - ώ6

Sample	N	Mean	SD	SE	Coefficient of variation
AHSO	3	53.656	0.037	0.021	6.927×10^{-4}
SHSO	3	56.387	0.061	0.035	0.001
THSO	3	54.486	0.263	0.152	0.005

Post Hoc Tests

Standard (HSD)

Post Hoc Comparisons - Sample

		Mean Difference	95% CI for Mean Difference		SE	df	t	p_{tukey}
			Lower	Upper				
AHSO	SHSO	-2.730	-3.125	-2.336	0.129	6	-21.231	< .001***
	THSO	-0.829	-1.224	-0.435	0.129	6	-6.449	0.002**
SHSO	THSO	1.901	1.506	2.296	0.129	6	14.782	< .001***

** p < .01, *** p < .001

Note. P-value and confidence intervals adjusted for comparing a family of 3 estimates (confidence intervals corrected using the tukey method).

Letter-Based Grouping - Sample

Sample	Letter
AHSO	a
SHSO	c
THSO	b

Note. If two or more means share the same grouping symbol, then we cannot show them to be different, but we also did not show them to be the same.

ANOVA

ANOVA - PUFA/SFA

Homogeneity Correction	Cases	Sum of Squares	df	Mean Square	F	p
Welch	Sample	2.254	2.000	1.127	252.581	< .001
	Residuals	0.051	3.483	0.015		

Note. Type III Sum of Squares

Descriptives

Descriptives - PUFA/SFA

Sample	N	Mean	SD	SE	Coefficient of variation
AHSO	3	5.171	0.044	0.026	0.009
SHSO	3	6.394	0.075	0.043	0.012
THSO	3	5.703	0.134	0.078	0.024

Post Hoc Tests

Standard (HSD)

Post Hoc Comparisons - Sample

		Mean Difference	95% CI for Mean Difference Lower	95% CI for Mean Difference Upper	SE	df	t	p_{tukey}
AHSO	SHSO	-1.222	-1.454	-0.991	0.075	6	-16.203	< .001***
	THSO	-0.531	-0.763	-0.300	0.075	6	-7.043	< .001***
SHSO	THSO	0.691	0.460	0.922	0.075	6	9.160	< .001***

*** p < .001

Note. P-value and confidence intervals adjusted for comparing a family of 3 estimates (confidence intervals corrected using the tukey method).

Letter-Based Grouping - Sample

Sample	Letter
AHSO	a
SHSO	c
THSO	b

Note. If two or more means share the same grouping symbol, then we cannot show them to be different, but we also did not show them to be the same.

ANOVA

ANOVA - ω-6 / ω-3

Homogeneity Correction	Cases	Sum of Squares	df	Mean Square	F	p
Welch	Sample	4.307	2.000	2.154	1020.722	< .001
	Residuals	0.008	3.880	0.002		

Note. Type III Sum of Squares

Descriptives

Descriptives - ω-6 / ω-3

Sample	N	Mean	SD	SE	Coefficient of variation
AHSO	3	4.258	0.030	0.017	0.007
SHSO	3	3.926	0.032	0.019	0.008
THSO	3	5.531	0.048	0.028	0.009

Post Hoc Tests

Standard (HSD)

Post Hoc Comparisons - Sample

		Mean Difference	95% CI for Mean Difference Lower	95% CI for Mean Difference Upper	SE	df	t	p_{tukey}
AHSO	SHSO	0.333	0.239	0.426	0.030	6	10.916	< .001***
	THSO	-1.273	-1.366	-1.179	0.030	6	-41.759	< .001***
SHSO	THSO	-1.605	-1.699	-1.512	0.030	6	-52.675	< .001***

*** p < .001
Note. P-value and confidence intervals adjusted for comparing a family of 3 estimates (confidence intervals corrected using the tukey method).

Letter-Based Grouping - Sample

Sample	Letter
AHSO	b
SHSO	a
THSO	c

Note. If two or more means share the same grouping symbol, then we cannot show them to be different, but we also did not show them to be the same.

ANOVA

ANOVA - TPC

Homogeneity Correction	Cases	Sum of Squares	df	Mean Square	F	p
Welch	Sample	2706.717	2.000	1353.359	6090.986	< .001
	Residuals	1.088	3.039	0.358		

Note. Type III Sum of Squares

Descriptives

Descriptives - TPC

Sample	N	Mean	SD	SE	Coefficient of variation
AHSO	3	118.710	0.606	0.350	0.005
SHSO	3	148.260	0.398	0.230	0.003
THSO	3	159.913	0.133	0.077	8.304×10^{-4}

Post Hoc Tests

Standard (HSD)

Post Hoc Comparisons - Sample

		Mean Difference	95% CI for Mean Difference		SE	df	t	p_{tukey}
			Lower	Upper				
AHSO	SHSO	-29.550	-30.617	-28.483	0.348	6	-85.002	< .001***
	THSO	-41.203	-42.270	-40.137	0.348	6	-118.524	< .001***
SHSO	THSO	-11.653	-12.720	-10.587	0.348	6	-33.522	< .001***

*** p < .001
Note. P-value and confidence intervals adjusted for comparing a family of 3 estimates (confidence intervals corrected using the tukey method).

Letter-Based Grouping - Sample

Sample	Letter
AHSO	a
SHSO	b
THSO	c

Note. If two or more means share the same grouping symbol, then we cannot show them to be different, but we also did not show them to be the same.

ANOVA

ANOVA - DPPH

Homogeneity Correction	Cases	Sum of Squares	df	Mean Square	F	p
Welch	Sample	87.146	2.000	43.573	4549.495	< .001
	Residuals	0.066	3.584	0.019		

Note. Type III Sum of Squares

Descriptives

Descriptives - DPPH

Sample	N	Mean	SD	SE	Coefficient of variation
AHSO	3	35.920	0.140	0.081	0.004
SHSO	3	38.250	0.100	0.058	0.003
THSO	3	43.370	0.060	0.035	0.001

Post Hoc Tests

Standard (HSD)

Post Hoc Comparisons - Sample

		Mean Difference	95% CI for Mean Difference Lower	95% CI for Mean Difference Upper	SE	df	t	p_{tukey}
AHSO	SHSO	-2.330	-2.594	-2.066	0.086	6	-27.126	< .001***
	THSO	-7.450	-7.714	-7.186	0.086	6	-86.735	< .001***
SHSO	THSO	-5.120	-5.384	-4.856	0.086	6	-59.608	< .001***

*** p < .001

Note. P-value and confidence intervals adjusted for comparing a family of 3 estimates (confidence intervals corrected using the tukey method).

Letter-Based Grouping - Sample

Sample	Letter
AHSO	a
SHSO	b
THSO	c

Note. If two or more means share the same grouping symbol, then we cannot show them to be different, but we also did not show them to be the same.

ANOVA

ANOVA - chlorophyll a+b

Homogeneity Correction	Cases	Sum of Squares	df	Mean Square	F	p
Welch	Sample	6.988	2.000	3.494	928.861	< .001
	Residuals	0.029	3.707	0.008		

Note. Type III Sum of Squares

Descriptives

Descriptives - chlorophyll a+b

Sample	N	Mean	SD	SE	Coefficient of variation
AHSO	3	8.313	0.105	0.061	0.013
SHSO	3	6.177	0.040	0.023	0.007
THSO	3	7.510	0.040	0.023	0.005

Post Hoc Tests

Standard (HSD)

Post Hoc Comparisons - Sample

		Mean Difference	95% CI for Mean Difference		SE	df	t	p_{tukey}
			Lower	Upper				
AHSO	SHSO	2.137	1.964	2.309	0.056	6	37.947	< .001***
	THSO	0.803	0.631	0.976	0.056	6	14.267	< .001***
SHSO	THSO	-1.333	-1.506	-1.161	0.056	6	-23.680	< .001***

*** p < .001

Note. P-value and confidence intervals adjusted for comparing a family of 3 estimates (confidence intervals corrected using the tukey method).

Letter-Based Grouping - Sample

Sample	Letter
AHSO	c
SHSO	a
THSO	b

Note. If two or more means share the same grouping symbol, then we cannot show them to be different, but we also did not show them to be the same.

ANOVA

ANOVA - carotenoids

Homogeneity Correction	Cases	Sum of Squares	df	Mean Square	F	p
Welch	Sample	0.939	2.000	0.470	2054.238	< .001
	Residuals	0.002	3.349	6.171×10^{-4}		

Note. Type III Sum of Squares

Descriptives

Descriptives - carotenoids

sample	N	Mean	SD	SE	Coefficient of variation
AHSO	3	3.370	0.030	0.017	0.009
SHSO	3	3.013	0.006	0.003	0.002
THSO	3	2.580	0.010	0.006	0.004

Post Hoc Tests

Standard (HSD)

Post Hoc Comparisons - sample

		Mean Difference	95% CI for Mean Difference		SE	df	t	p_{tukey}
			Lower	Upper				
AHSO	SHSO	0.357	0.310	0.403	0.015	6	23.537	< .001***
	THSO	0.790	0.744	0.836	0.015	6	52.133	< .001***
SHSO	THSO	0.433	0.387	0.480	0.015	6	28.596	< .001***

*** p < .001
Note. P-value and confidence intervals adjusted for comparing a family of 3 estimates (confidence intervals corrected using the tukey method).

Letter-Based Grouping - sample

sample	Letter
AHSO	c
SHSO	b
THSO	a

Note. If two or more means share the same grouping symbol, then we cannot show them to be different, but we also did not show them to be the same.

ANOVA

ANOVA - chlorophyll/carotenoids

Cases	Sum of Squares	df	Mean Square	F	p
sample	1.103	2	0.551	3117.982	< .001
Residuals	0.001	6	1.769×10^{-4}		

Note. Type III Sum of Squares

Descriptives

Descriptives - chlorophyll/carotenoids

sample	N	Mean	SD	SE	Coefficient of variation
AHSO	3	2.467	0.012	0.007	0.005
SHSO	3	2.908	0.012	0.007	0.004
THSO	3	2.050	0.016	0.009	0.008

Post Hoc Tests

Standard (HSD)

Post Hoc Comparisons - sample

			95% CI for Mean Difference					
		Mean Difference	Lower	Upper	SE	df	t	p_{tukey}
AHSO	SHSO	-0.441	-0.474	-0.408	0.011	6	-40.596	< .001***
	THSO	0.417	0.383	0.450	0.011	6	38.361	< .001***
SHSO	THSO	0.857	0.824	0.891	0.011	6	78.958	< .001***

*** p < .001

Note. P-value and confidence intervals adjusted for comparing a family of 3 estimates (confidence intervals corrected using the tukey method).

Letter-Based Grouping - sample

sample	Letter
AHSO	b
SHSO	c
THSO	a

Note. If two or more means share the same grouping symbol, then we cannot show them to be different, but we also did not show them to be the same.
