# Supplementary material for: Chemical Profile, Bioactive Constituents and In Vitro Growth Stimulation Properties of Cold-Pressed Hemp Seed Oils from Romanian Varieties: In Vitro and In Silico Evaluation
Source: Plants (Basel). 2025 Nov 13;14(22):3465. doi: 10.3390/plants14223465 (PMC12655998; doi:10.3390/plants14223465)
Supplement: Supplementary file 1 [file plants-14-03465-s001.zip › Supplementary file S2_In-vitro probiotic data and statistical analysis.pdf]

Supplementary file S2

ANOVA

ANOVA - Lactocaseibacillus rhamnosus (GG; HN001)

Cases	Sum of Squares	df	Mean Square	F	p
sample	27.184	2	13.592	29735.379	< .001
concentration	10203.982	6	1700.664	$3.721 \times 10^{+6}$	< .001
sample ✻ concentration	2090.409	12	174.201	381103.802	< .001
Residuals	0.019	42	4.571×10^{-4}		

Note. Type III Sum of Squares

Descriptives

Descriptives - Lactocaseibacillus rhamnosus (GG; HN001)

sample	concentration	N	Mean	SD	SE	Coefficient of variation
AHSO	0.3	3	-26.240	0.012	0.007	-4.573×10^{-4}
	0.6	3	-15.030	0.028	0.016	-0.002
	1.3	3	-13.860	0.015	0.009	-0.001
	2.5	3	-12.490	0.024	0.014	-0.002
	5	3	-12.060	0.013	0.008	-0.001
	9	3	-10.900	0.025	0.014	-0.002
	16	3	-6.880	0.023	0.013	-0.003
SHSO	0.3	3	-51.320	0.026	0.015	-5.066×10^{-4}
	0.6	3	-17.350	0.019	0.011	-0.001
	1.3	3	-13.650	0.021	0.012	-0.002
	2.5	3	-12.910	0.011	0.006	-8.521×10^{-4}
	5	3	-6.670	0.027	0.016	-0.004
	9	3	-4.760	0.016	0.009	-0.003
	16	3	16.830	0.030	0.017	0.002
THSO	0.3	3	-43.810	0.017	0.010	-3.880×10^{-4}
	0.6	3	-13.970	0.023	0.013	-0.002
	1.3	3	-12.700	0.014	0.008	-0.001
	2.5	3	-10.900	0.029	0.017	-0.003
	5	3	-7.940	0.018	0.010	-0.002
	9	3	0.420	0.022	0.013	0.052
	16	3	2.430	0.020	0.012	0.008

Post Hoc Tests

Standard (HSD)

Post Hoc Comparisons - sample ✻ concentration

		Mean Difference	95% CI for Mean Difference Lower	95% CI for Mean Difference Upper	SE	df	t	p_{tukey}
AHSO 0.3	SHSO 0.3	25.080	25.014	25.146	0.017	42	1436.712	< .001***
	THSO 0.3	17.570	17.504	17.636	0.017	42	1006.501	< .001***
	AHSO 0.6	-11.210	-11.276	-11.144	0.017	42	-642.167	< .001***
	SHSO 0.6	-8.890	-8.956	-8.824	0.017	42	-509.265	< .001***
	THSO 0.6	-12.270	-12.336	-12.204	0.017	42	-702.889	< .001***
	AHSO 1.3	-12.380	-12.446	-12.314	0.017	42	-709.191	< .001***
	SHSO 1.3	-12.590	-12.656	-12.524	0.017	42	-721.220	< .001***
	THSO 1.3	-13.540	-13.606	-13.474	0.017	42	-775.641	< .001***
	AHSO 2.5	-13.750	-13.816	-13.684	0.017	42	-787.671	< .001***
	SHSO 2.5	-13.330	-13.396	-13.264	0.017	42	-763.611	< .001***
	THSO 2.5	-15.340	-15.406	-15.274	0.017	42	-878.755	< .001***
	AHSO 5	-14.180	-14.246	-14.114	0.017	42	-812.304	< .001***
	SHSO 5	-19.570	-19.636	-19.504	0.017	42	-1121.071	< .001***
	THSO 5	-18.300	-18.366	-18.234	0.017	42	-1048.319	< .001***
	AHSO 9	-15.340	-15.406	-15.274	0.017	42	-878.755	< .001***
	SHSO 9	-21.480	-21.546	-21.414	0.017	42	-1230.486	< .001***
	THSO 9	-26.660	-26.726	-26.594	0.017	42	-1527.223	< .001***
	AHSO 16	-19.360	-19.426	-19.294	0.017	42	-1109.041	< .001***
	SHSO 16	-43.070	-43.136	-43.004	0.017	42	-2467.273	< .001***
	THSO 16	-28.670	-28.736	-28.604	0.017	42	-1642.366	< .001***
SHSO 0.3	THSO 0.3	-7.510	-7.576	-7.444	0.017	42	-430.212	< .001***
	AHSO 0.6	-36.290	-36.356	-36.224	0.017	42	-2078.879	< .001***
	SHSO 0.6	-33.970	-34.036	-33.904	0.017	42	-1945.978	< .001***
	THSO 0.6	-37.350	-37.416	-37.284	0.017	42	-2139.601	< .001***

Post Hoc Comparisons - sample ✻ concentration

		Mean Difference	95% CI for Mean Difference Lower	95% CI for Mean Difference Upper	SE	df	t	p_{tukey}
	AHSO 1.3	-37.460	-37.526	-37.394	0.017	42	-2145.903	< .001***
	SHSO 1.3	-37.670	-37.736	-37.604	0.017	42	-2157.933	< .001***
	THSO 1.3	-38.620	-38.686	-38.554	0.017	42	-2212.354	< .001***
	AHSO 2.5	-38.830	-38.896	-38.764	0.017	42	-2224.384	< .001***
	SHSO 2.5	-38.410	-38.476	-38.344	0.017	42	-2200.324	< .001***
	THSO 2.5	-40.420	-40.486	-40.354	0.017	42	-2315.467	< .001***
	AHSO 5	-39.260	-39.326	-39.194	0.017	42	-2249.016	< .001***
	SHSO 5	-44.650	-44.716	-44.584	0.017	42	-2557.783	< .001***
	THSO 5	-43.380	-43.446	-43.314	0.017	42	-2485.031	< .001***
	AHSO 9	-40.420	-40.486	-40.354	0.017	42	-2315.467	< .001***
	SHSO 9	-46.560	-46.626	-46.494	0.017	42	-2667.198	< .001***
	THSO 9	-51.740	-51.806	-51.674	0.017	42	-2963.935	< .001***
	AHSO 16	-44.440	-44.506	-44.374	0.017	42	-2545.753	< .001***
	SHSO 16	-68.150	-68.216	-68.084	0.017	42	-3903.985	< .001***
	THSO 16	-53.750	-53.816	-53.684	0.017	42	-3079.078	< .001***
THSO 0.3	AHSO 0.6	-28.780	-28.846	-28.714	0.017	42	-1648.667	< .001***
	SHSO 0.6	-26.460	-26.526	-26.394	0.017	42	-1515.766	< .001***
	THSO 0.6	-29.840	-29.906	-29.774	0.017	42	-1709.390	< .001***
	AHSO 1.3	-29.950	-30.016	-29.884	0.017	42	-1715.691	< .001***
	SHSO 1.3	-30.160	-30.226	-30.094	0.017	42	-1727.721	< .001***
	THSO 1.3	-31.110	-31.176	-31.044	0.017	42	-1782.142	< .001***
	AHSO 2.5	-31.320	-31.386	-31.254	0.017	42	-1794.172	< .001***
	SHSO 2.5	-30.900	-30.966	-30.834	0.017	42	-1770.112	< .001***
	THSO 2.5	-32.910	-32.976	-32.844	0.017	42	-1885.255	< .001***
	AHSO 5	-31.750	-31.816	-31.684	0.017	42	-1818.804	< .001***

Post Hoc Comparisons - sample ✻ concentration

		Mean Difference	95% CI for Mean Difference		SE	df	t	p_{tukey}
			Lower	Upper				
	SHSO 5	-37.140	-37.206	-37.074	0.017	42	-2127.572	< .001***
	THSO 5	-35.870	-35.936	-35.804	0.017	42	-2054.819	< .001***
	AHSO 9	-32.910	-32.976	-32.844	0.017	42	-1885.255	< .001***
	SHSO 9	-39.050	-39.116	-38.984	0.017	42	-2236.986	< .001***
	THSO 9	-44.230	-44.296	-44.164	0.017	42	-2533.724	< .001***
	AHSO 16	-36.930	-36.996	-36.864	0.017	42	-2115.542	< .001***
	SHSO 16	-60.640	-60.706	-60.574	0.017	42	-3473.773	< .001***
	THSO 16	-46.240	-46.306	-46.174	0.017	42	-2648.867	< .001***
AHSO 0.6	SHSO 0.6	2.320	2.254	2.386	0.017	42	132.902	< .001***
	THSO 0.6	-1.060	-1.126	-0.994	0.017	42	-60.722	< .001***
	AHSO 1.3	-1.170	-1.236	-1.104	0.017	42	-67.024	< .001***
	SHSO 1.3	-1.380	-1.446	-1.314	0.017	42	-79.054	< .001***
	THSO 1.3	-2.330	-2.396	-2.264	0.017	42	-133.474	< .001***
	AHSO 2.5	-2.540	-2.606	-2.474	0.017	42	-145.504	< .001***
	SHSO 2.5	-2.120	-2.186	-2.054	0.017	42	-121.445	< .001***
	THSO 2.5	-4.130	-4.196	-4.064	0.017	42	-236.588	< .001***
	AHSO 5	-2.970	-3.036	-2.904	0.017	42	-170.137	< .001***
	SHSO 5	-8.360	-8.426	-8.294	0.017	42	-478.904	< .001***
	THSO 5	-7.090	-7.156	-7.024	0.017	42	-406.152	< .001***
	AHSO 9	-4.130	-4.196	-4.064	0.017	42	-236.588	< .001***
	SHSO 9	-10.270	-10.336	-10.204	0.017	42	-588.319	< .001***
	THSO 9	-15.450	-15.516	-15.384	0.017	42	-885.056	< .001***
	AHSO 16	-8.150	-8.216	-8.084	0.017	42	-466.874	< .001***
	SHSO 16	-31.860	-31.926	-31.794	0.017	42	-1825.106	< .001***
	THSO 16	-17.460	-17.526	-17.394	0.017	42	-1000.199	< .001***
SHSO 0.6	THSO 0.6	-3.380	-3.446	-3.314	0.017	42	-193.624	< .001***
	AHSO 1.3	-3.490	-3.556	-3.424	0.017	42	-199.925	< .001***

Post Hoc Comparisons - sample ✻ concentration

			95% CI for Mean Difference					
		Mean Difference	Lower	Upper	SE	df	t	p_{tukey}
	SHSO 1.3	-3.700	-3.766	-3.634	0.017	42	-211.955	< .001***
	THSO 1.3	-4.650	-4.716	-4.584	0.017	42	-266.376	< .001***
	AHSO 2.5	-4.860	-4.926	-4.794	0.017	42	-278.406	< .001***
	SHSO 2.5	-4.440	-4.506	-4.374	0.017	42	-254.346	< .001***
	THSO 2.5	-6.450	-6.516	-6.384	0.017	42	-369.489	< .001***
	AHSO 5	-5.290	-5.356	-5.224	0.017	42	-303.039	< .001***
	SHSO 5	-10.680	-10.746	-10.614	0.017	42	-611.806	< .001***
	THSO 5	-9.410	-9.476	-9.344	0.017	42	-539.054	< .001***
	AHSO 9	-6.450	-6.516	-6.384	0.017	42	-369.489	< .001***
	SHSO 9	-12.590	-12.656	-12.524	0.017	42	-721.220	< .001***
	THSO 9	-17.770	-17.836	-17.704	0.017	42	-1017.958	< .001***
	AHSO 16	-10.470	-10.536	-10.404	0.017	42	-599.776	< .001***
	SHSO 16	-34.180	-34.246	-34.114	0.017	42	-1958.007	< .001***
	THSO 16	-19.780	-19.846	-19.714	0.017	42	-1133.101	< .001***
THSO 0.6	AHSO 1.3	-0.110	-0.176	-0.044	0.017	42	-6.301	< .001***
	SHSO 1.3	-0.320	-0.386	-0.254	0.017	42	-18.331	< .001***
	THSO 1.3	-1.270	-1.336	-1.204	0.017	42	-72.752	< .001***
	AHSO 2.5	-1.480	-1.546	-1.414	0.017	42	-84.782	< .001***
	SHSO 2.5	-1.060	-1.126	-0.994	0.017	42	-60.722	< .001***
	THSO 2.5	-3.070	-3.136	-3.004	0.017	42	-175.866	< .001***
	AHSO 5	-1.910	-1.976	-1.844	0.017	42	-109.415	< .001***
	SHSO 5	-7.300	-7.366	-7.234	0.017	42	-418.182	< .001***
	THSO 5	-6.030	-6.096	-5.964	0.017	42	-345.430	< .001***
	AHSO 9	-3.070	-3.136	-3.004	0.017	42	-175.866	< .001***
	SHSO 9	-9.210	-9.276	-9.144	0.017	42	-527.597	< .001***
	THSO 9	-14.390	-14.456	-14.324	0.017	42	-824.334	< .001***
	AHSO 16	-7.090	-7.156	-7.024	0.017	42	-406.152	< .001***

Post Hoc Comparisons - sample ✲ concentration

		Mean Difference	95% CI for Mean Difference		SE	df	t	p_{tukey}
			Lower	Upper				
	SHSO 16	-30.800	-30.866	-30.734	0.017	42	-1764.384	< .001***
	THSO 16	-16.400	-16.466	-16.334	0.017	42	-939.477	< .001***
AHSO 1.3	SHSO 1.3	-0.210	-0.276	-0.144	0.017	42	-12.030	< .001***
	THSO 1.3	-1.160	-1.226	-1.094	0.017	42	-66.451	< .001***
	AHSO 2.5	-1.370	-1.436	-1.304	0.017	42	-78.481	< .001***
	SHSO 2.5	-0.950	-1.016	-0.884	0.017	42	-54.421	< .001***
	THSO 2.5	-2.960	-3.026	-2.894	0.017	42	-169.564	< .001***
	AHSO 5	-1.800	-1.866	-1.734	0.017	42	-103.113	< .001***
	SHSO 5	-7.190	-7.256	-7.124	0.017	42	-411.880	< .001***
	THSO 5	-5.920	-5.986	-5.854	0.017	42	-339.128	< .001***
	AHSO 9	-2.960	-3.026	-2.894	0.017	42	-169.564	< .001***
	SHSO 9	-9.100	-9.166	-9.034	0.017	42	-521.295	< .001***
	THSO 9	-14.280	-14.346	-14.214	0.017	42	-818.032	< .001***
	AHSO 16	-6.980	-7.046	-6.914	0.017	42	-399.851	< .001***
	SHSO 16	-30.690	-30.756	-30.624	0.017	42	-1758.082	< .001***
	THSO 16	-16.290	-16.356	-16.224	0.017	42	-933.176	< .001***
SHSO 1.3	THSO 1.3	-0.950	-1.016	-0.884	0.017	42	-54.421	< .001***
	AHSO 2.5	-1.160	-1.226	-1.094	0.017	42	-66.451	< .001***
	SHSO 2.5	-0.740	-0.806	-0.674	0.017	42	-42.391	< .001***
	THSO 2.5	-2.750	-2.816	-2.684	0.017	42	-157.534	< .001***
	AHSO 5	-1.590	-1.656	-1.524	0.017	42	-91.083	< .001***
	SHSO 5	-6.980	-7.046	-6.914	0.017	42	-399.851	< .001***
	THSO 5	-5.710	-5.776	-5.644	0.017	42	-327.098	< .001***
	AHSO 9	-2.750	-2.816	-2.684	0.017	42	-157.534	< .001***
	SHSO 9	-8.890	-8.956	-8.824	0.017	42	-509.265	< .001***
	THSO 9	-14.070	-14.136	-14.004	0.017	42	-806.002	< .001***
	AHSO 16	-6.770	-6.836	-6.704	0.017	42	-387.821	< .001***

Post Hoc Comparisons - sample ✻ concentration

		Mean Difference	95% CI for Mean Difference		SE	df	t	p_{tukey}
			Lower	Upper				
	SHSO 16	-30.480	-30.546	-30.414	0.017	42	-1746.052	< .001***
	THSO 16	-16.080	-16.146	-16.014	0.017	42	-921.146	< .001***
THSO 1.3	AHSO 2.5	-0.210	-0.276	-0.144	0.017	42	-12.030	< .001***
	SHSO 2.5	0.210	0.144	0.276	0.017	42	12.030	< .001***
	THSO 2.5	-1.800	-1.866	-1.734	0.017	42	-103.113	< .001***
	AHSO 5	-0.640	-0.706	-0.574	0.017	42	-36.663	< .001***
	SHSO 5	-6.030	-6.096	-5.964	0.017	42	-345.430	< .001***
	THSO 5	-4.760	-4.826	-4.694	0.017	42	-272.677	< .001***
	AHSO 9	-1.800	-1.866	-1.734	0.017	42	-103.113	< .001***
	SHSO 9	-7.940	-8.006	-7.874	0.017	42	-454.844	< .001***
	THSO 9	-13.120	-13.186	-13.054	0.017	42	-751.582	< .001***
	AHSO 16	-5.820	-5.886	-5.754	0.017	42	-333.400	< .001***
	SHSO 16	-29.530	-29.596	-29.464	0.017	42	-1691.631	< .001***
	THSO 16	-15.130	-15.196	-15.064	0.017	42	-866.725	< .001***
AHSO 2.5	SHSO 2.5	0.420	0.354	0.486	0.017	42	24.060	< .001***
	THSO 2.5	-1.590	-1.656	-1.524	0.017	42	-91.083	< .001***
	AHSO 5	-0.430	-0.496	-0.364	0.017	42	-24.633	< .001***
	SHSO 5	-5.820	-5.886	-5.754	0.017	42	-333.400	< .001***
	THSO 5	-4.550	-4.616	-4.484	0.017	42	-260.648	< .001***
	AHSO 9	-1.590	-1.656	-1.524	0.017	42	-91.083	< .001***
	SHSO 9	-7.730	-7.796	-7.664	0.017	42	-442.814	< .001***
	THSO 9	-12.910	-12.976	-12.844	0.017	42	-739.552	< .001***
	AHSO 16	-5.610	-5.676	-5.544	0.017	42	-321.370	< .001***
	SHSO 16	-29.320	-29.386	-29.254	0.017	42	-1679.601	< .001***
	THSO 16	-14.920	-14.986	-14.854	0.017	42	-854.695	< .001***
SHSO 2.5	THSO 2.5	-2.010	-2.076	-1.944	0.017	42	-115.143	< .001***
	AHSO 5	-0.850	-0.916	-0.784	0.017	42	-48.692	< .001***
	SHSO 5	-6.240	-6.306	-6.174	0.017	42	-357.460	< .001***
	THSO 5	-4.970	-5.036	-4.904	0.017	42	-284.707	< .001***

Post Hoc Comparisons - sample ✻ concentration

		Mean Difference	95% CI for Mean Difference		SE	df	t	p_{tukey}
			Lower	Upper				
	AHSO 9	-2.010	-2.076	-1.944	0.017	42	-115.143	< .001***
	SHSO 9	-8.150	-8.216	-8.084	0.017	42	-466.874	< .001***
	THSO 9	-13.330	-13.396	-13.264	0.017	42	-763.611	< .001***
	AHSO 16	-6.030	-6.096	-5.964	0.017	42	-345.430	< .001***
	SHSO 16	-29.740	-29.806	-29.674	0.017	42	-1703.661	< .001***
	THSO 16	-15.340	-15.406	-15.274	0.017	42	-878.755	< .001***
THSO 2.5	AHSO 5	1.160	1.094	1.226	0.017	42	66.451	< .001***
	SHSO 5	-4.230	-4.296	-4.164	0.017	42	-242.316	< .001***
	THSO 5	-2.960	-3.026	-2.894	0.017	42	-169.564	< .001***
	AHSO 9	6.661×10^{-16}	-0.066	0.066	0.017	42	3.816×10^{-14}	1.000
	SHSO 9	-6.140	-6.206	-6.074	0.017	42	-351.731	< .001***
	THSO 9	-11.320	-11.386	-11.254	0.017	42	-648.468	< .001***
	AHSO 16	-4.020	-4.086	-3.954	0.017	42	-230.286	< .001***
	SHSO 16	-27.730	-27.796	-27.664	0.017	42	-1588.518	< .001***
	THSO 16	-13.330	-13.396	-13.264	0.017	42	-763.611	< .001***
AHSO 5	SHSO 5	-5.390	-5.456	-5.324	0.017	42	-308.767	< .001***
	THSO 5	-4.120	-4.186	-4.054	0.017	42	-236.015	< .001***
	AHSO 9	-1.160	-1.226	-1.094	0.017	42	-66.451	< .001***
	SHSO 9	-7.300	-7.366	-7.234	0.017	42	-418.182	< .001***
	THSO 9	-12.480	-12.546	-12.414	0.017	42	-714.919	< .001***
	AHSO 16	-5.180	-5.246	-5.114	0.017	42	-296.737	< .001***
	SHSO 16	-28.890	-28.956	-28.824	0.017	42	-1654.969	< .001***
	THSO 16	-14.490	-14.556	-14.424	0.017	42	-830.062	< .001***
SHSO 5	THSO 5	1.270	1.204	1.336	0.017	42	72.752	< .001***
	AHSO 9	4.230	4.164	4.296	0.017	42	242.316	< .001***
	SHSO 9	-1.910	-1.976	-1.844	0.017	42	-109.415	< .001***
	THSO 9	-7.090	-7.156	-7.024	0.017	42	-406.152	< .001***
	AHSO 16	0.210	0.144	0.276	0.017	42	12.030	< .001***
	SHSO 16	-23.500	-23.566	-23.434	0.017	42	-1346.202	< .001***

Post Hoc Comparisons - sample ✻ concentration

		Mean Difference	95% CI for Mean Difference		SE	df	t	p_{tukey}
			Lower	Upper				
	THSO 16	-9.100	-9.166	-9.034	0.017	42	-521.295	< .001***
THSO 5	AHSO 9	2.960	2.894	3.026	0.017	42	169.564	< .001***
	SHSO 9	-3.180	-3.246	-3.114	0.017	42	-182.167	< .001***
	THSO 9	-8.360	-8.426	-8.294	0.017	42	-478.904	< .001***
	AHSO 16	-1.060	-1.126	-0.994	0.017	42	-60.722	< .001***
	SHSO 16	-24.770	-24.836	-24.704	0.017	42	-1418.954	< .001***
	THSO 16	-10.370	-10.436	-10.304	0.017	42	-594.047	< .001***
AHSO 9	SHSO 9	-6.140	-6.206	-6.074	0.017	42	-351.731	< .001***
	THSO 9	-11.320	-11.386	-11.254	0.017	42	-648.468	< .001***
	AHSO 16	-4.020	-4.086	-3.954	0.017	42	-230.286	< .001***
	SHSO 16	-27.730	-27.796	-27.664	0.017	42	-1588.518	< .001***
	THSO 16	-13.330	-13.396	-13.264	0.017	42	-763.611	< .001***
SHSO 9	THSO 9	-5.180	-5.246	-5.114	0.017	42	-296.737	< .001***
	AHSO 16	2.120	2.054	2.186	0.017	42	121.445	< .001***
	SHSO 16	-21.590	-21.656	-21.524	0.017	42	-1236.787	< .001***
	THSO 16	-7.190	-7.256	-7.124	0.017	42	-411.880	< .001***
THSO 9	AHSO 16	7.300	7.234	7.366	0.017	42	418.182	< .001***
	SHSO 16	-16.410	-16.476	-16.344	0.017	42	-940.050	< .001***
	THSO 16	-2.010	-2.076	-1.944	0.017	42	-115.143	< .001***
AHSO 16	SHSO 16	-23.710	-23.776	-23.644	0.017	42	-1358.232	< .001***
	THSO 16	-9.310	-9.376	-9.244	0.017	42	-533.325	< .001***
SHSO 16	THSO 16	14.400	14.334	14.466	0.017	42	824.907	< .001***

*** $p < .001$

Note. P-value and confidence intervals adjusted for comparing a family of 21 estimates (confidence intervals corrected using the tukey method).

Letter-Based Grouping - sample ✻ concentration

sample	concentration	Letter
AHSO	0.3	c
SHSO		a
THSO		b
AHSO	0.6	e
SHSO		d
THSO		f
AHSO	1.3	g
SHSO		h
THSO		j
AHSO	2.5	k
SHSO		i
THSO		m
AHSO	5	l
SHSO		p
THSO		n
AHSO	9	m
SHSO		q
THSO		r
AHSO	16	o
SHSO		t
THSO		s

Note. If two or more means share the same grouping symbol, then we cannot show them to be different, but we also did not show them to be the same.

ANOVA

ANOVA - Lactobacillus paracasei subsp.paracasei ATCC BAA-52

Cases	Sum of Squares	df	Mean Square	F	p
sample	74.794	2	37.397	88003.037	< .001
concentration	7215.136	6	1202.523	$2.830 \times 10^{+6}$	< .001
sample ✻ concentration	2672.471	12	222.706	524072.602	< .001
Residuals	0.018	42	4.250×10^{-4}		

Note. Type III Sum of Squares

Descriptives

Descriptives - Lactobacillus paracasei subsp.paracasei ATCC BAA-52

sample	concentration	N	Mean	SD	SE	Coefficient of variation
AHSO	0.3	3	-30.890	0.022	0.013	-7.122×10^{-4}

Descriptives - Lactobacillus paracasei subsp.paracasei ATCC BAA-52

sample	concentration	N	Mean	SD	SE	Coefficient of variation
	0.6	3	-10.620	0.023	0.013	-0.002
	1.3	3	-9.640	0.025	0.014	-0.003
	2.5	3	-9.310	0.021	0.012	-0.002
	5	3	-9.200	0.018	0.010	-0.002
	9	3	-9.090	0.026	0.015	-0.003
	16	3	-8.320	0.023	0.013	-0.003
SHSO	0.3	3	-23.660	0.012	0.007	-5.072×10^{-4}
	0.6	3	-12.050	0.015	0.009	-0.001
	1.3	3	-10.840	0.019	0.011	-0.002
	2.5	3	-9.420	0.027	0.016	-0.003
	5	3	-8.980	0.029	0.017	-0.003
	9	3	-7.780	0.016	0.009	-0.002
	16	3	-3.610	0.015	0.009	-0.004
THSO	0.3	3	-49.620	0.017	0.010	-3.426×10^{-4}
	0.6	3	-14.460	0.028	0.016	-0.002
	1.3	3	-10.620	0.011	0.006	-0.001
	2.5	3	-9.860	0.013	0.008	-0.001
	5	3	-3.400	0.014	0.008	-0.004
	9	3	-1.420	0.024	0.014	-0.017
	16	3	20.920	0.020	0.012	9.560×10^{-4}

Post Hoc Tests

Standard (HSD)

Post Hoc Comparisons - sample ✻ concentration

			95% CI for Mean Difference					
		Mean Difference	Lower	Upper	SE	df	t	p_{tukey}
AHSO 0.3	SHSO 0.3	-7.230	-7.294	-7.166	0.017	42	-429.550	< .001***
	THSO 0.3	18.730	18.666	18.794	0.017	42	1112.790	< .001***
	AHSO 0.6	-20.270	-20.334	-20.206	0.017	42	-1204.285	< .001***
	SHSO 0.6	-18.840	-18.904	-18.776	0.017	42	-1119.325	< .001***
	THSO 0.6	-16.430	-16.494	-16.366	0.017	42	-976.142	< .001***
	AHSO 1.3	-21.250	-21.314	-21.186	0.017	42	-1262.509	< .001***
	SHSO 1.3	-20.050	-20.114	-19.986	0.017	42	-1191.214	< .001***

Post Hoc Comparisons - sample ✲ concentration

		Mean Difference	95% CI for Mean Difference		SE	df	t	p_{tukey}
			Lower	Upper				
	THSO 1.3	-20.270	-20.334	-20.206	0.017	42	-1204.285	< .001***
	AHSO 2.5	-21.580	-21.644	-21.516	0.017	42	-1282.115	< .001***
	SHSO 2.5	-21.470	-21.534	-21.406	0.017	42	-1275.580	< .001***
	THSO 2.5	-21.030	-21.094	-20.966	0.017	42	-1249.438	< .001***
	AHSO 5	-21.690	-21.754	-21.626	0.017	42	-1288.650	< .001***
	SHSO 5	-21.910	-21.974	-21.846	0.017	42	-1301.721	< .001***
	THSO 5	-27.490	-27.554	-27.426	0.017	42	-1633.241	< .001***
	AHSO 9	-21.800	-21.864	-21.736	0.017	42	-1295.186	< .001***
	SHSO 9	-23.110	-23.174	-23.046	0.017	42	-1373.016	< .001***
	THSO 9	-29.470	-29.534	-29.406	0.017	42	-1750.877	< .001***
	AHSO 16	-22.570	-22.634	-22.506	0.017	42	-1340.933	< .001***
	SHSO 16	-27.280	-27.344	-27.216	0.017	42	-1620.764	< .001***
	THSO 16	-51.810	-51.874	-51.746	0.017	42	-3078.145	< .001***
SHSO 0.3	THSO 0.3	25.960	25.896	26.024	0.017	42	1542.340	< .001***
	AHSO 0.6	-13.040	-13.104	-12.976	0.017	42	-774.735	< .001***
	SHSO 0.6	-11.610	-11.674	-11.546	0.017	42	-689.775	< .001***
	THSO 0.6	-9.200	-9.264	-9.136	0.017	42	-546.592	< .001***
	AHSO 1.3	-14.020	-14.084	-13.956	0.017	42	-832.959	< .001***
	SHSO 1.3	-12.820	-12.884	-12.756	0.017	42	-761.664	< .001***
	THSO 1.3	-13.040	-13.104	-12.976	0.017	42	-774.735	< .001***
	AHSO 2.5	-14.350	-14.414	-14.286	0.017	42	-852.565	< .001***
	SHSO 2.5	-14.240	-14.304	-14.176	0.017	42	-846.029	< .001***
	THSO 2.5	-13.800	-13.864	-13.736	0.017	42	-819.888	< .001***
	AHSO 5	-14.460	-14.524	-14.396	0.017	42	-859.100	< .001***
	SHSO 5	-14.680	-14.744	-14.616	0.017	42	-872.171	< .001***
	THSO 5	-20.260	-20.324	-20.196	0.017	42	-1203.691	< .001***

Post Hoc Comparisons - sample ✻ concentration

		Mean Difference	95% CI for Mean Difference		SE	df	t	p_{tukey}
			Lower	Upper				
	AHSO 9	-14.570	-14.634	-14.506	0.017	42	-865.635	< .001***
	SHSO 9	-15.880	-15.944	-15.816	0.017	42	-943.465	< .001***
	THSO 9	-22.240	-22.304	-22.176	0.017	42	-1321.327	< .001***
	AHSO 16	-15.340	-15.404	-15.276	0.017	42	-911.383	< .001***
	SHSO 16	-20.050	-20.114	-19.986	0.017	42	-1191.214	< .001***
	THSO 16	-44.580	-44.644	-44.516	0.017	42	-2648.595	< .001***
THSO 0.3	AHSO 0.6	-39.000	-39.064	-38.936	0.017	42	-2317.075	< .001***
	SHSO 0.6	-37.570	-37.634	-37.506	0.017	42	-2232.116	< .001***
	THSO 0.6	-35.160	-35.224	-35.096	0.017	42	-2088.932	< .001***
	AHSO 1.3	-39.980	-40.044	-39.916	0.017	42	-2375.299	< .001***
	SHSO 1.3	-38.780	-38.844	-38.716	0.017	42	-2304.004	< .001***
	THSO 1.3	-39.000	-39.064	-38.936	0.017	42	-2317.075	< .001***
	AHSO 2.5	-40.310	-40.374	-40.246	0.017	42	-2394.905	< .001***
	SHSO 2.5	-40.200	-40.264	-40.136	0.017	42	-2388.370	< .001***
	THSO 2.5	-39.760	-39.824	-39.696	0.017	42	-2362.228	< .001***
	AHSO 5	-40.420	-40.484	-40.356	0.017	42	-2401.440	< .001***
	SHSO 5	-40.640	-40.704	-40.576	0.017	42	-2414.511	< .001***
	THSO 5	-46.220	-46.284	-46.156	0.017	42	-2746.031	< .001***
	AHSO 9	-40.530	-40.594	-40.466	0.017	42	-2407.976	< .001***
	SHSO 9	-41.840	-41.904	-41.776	0.017	42	-2485.806	< .001***
	THSO 9	-48.200	-48.264	-48.136	0.017	42	-2863.667	< .001***
	AHSO 16	-41.300	-41.364	-41.236	0.017	42	-2453.723	< .001***
	SHSO 16	-46.010	-46.074	-45.946	0.017	42	-2733.554	< .001***
	THSO 16	-70.540	-70.604	-70.476	0.017	42	-4190.935	< .001***
AHSO 0.6	SHSO 0.6	1.430	1.366	1.494	0.017	42	84.959	< .001***
	THSO 0.6	3.840	3.776	3.904	0.017	42	228.143	< .001***

Post Hoc Comparisons - sample ✻ concentration

		Mean Difference	95% CI for Mean Difference		SE	df	t	p_{tukey}
			Lower	Upper				
	AHSO 1.3	-0.980	-1.044	-0.916	0.017	42	-58.224	< .001***
	SHSO 1.3	0.220	0.156	0.284	0.017	42	13.071	< .001***
	THSO 1.3	-5.718×10^{-15}	-0.064	0.064	0.017	42	-3.397×10^{-13}	1.000
	AHSO 2.5	-1.310	-1.374	-1.246	0.017	42	-77.830	< .001***
	SHSO 2.5	-1.200	-1.264	-1.136	0.017	42	-71.295	< .001***
	THSO 2.5	-0.760	-0.824	-0.696	0.017	42	-45.153	< .001***
	AHSO 5	-1.420	-1.484	-1.356	0.017	42	-84.365	< .001***
	SHSO 5	-1.640	-1.704	-1.576	0.017	42	-97.436	< .001***
	THSO 5	-7.220	-7.284	-7.156	0.017	42	-428.956	< .001***
	AHSO 9	-1.530	-1.594	-1.466	0.017	42	-90.901	< .001***
	SHSO 9	-2.840	-2.904	-2.776	0.017	42	-168.731	< .001***
	THSO 9	-9.200	-9.264	-9.136	0.017	42	-546.592	< .001***
	AHSO 16	-2.300	-2.364	-2.236	0.017	42	-136.648	< .001***
	SHSO 16	-7.010	-7.074	-6.946	0.017	42	-416.479	< .001***
	THSO 16	-31.540	-31.604	-31.476	0.017	42	-1873.860	< .001***
SHSO 0.6	THSO 0.6	2.410	2.346	2.474	0.017	42	143.183	< .001***
	AHSO 1.3	-2.410	-2.474	-2.346	0.017	42	-143.183	< .001***
	SHSO 1.3	-1.210	-1.274	-1.146	0.017	42	-71.889	< .001***
	THSO 1.3	-1.430	-1.494	-1.366	0.017	42	-84.959	< .001***
	AHSO 2.5	-2.740	-2.804	-2.676	0.017	42	-162.789	< .001***
	SHSO 2.5	-2.630	-2.694	-2.566	0.017	42	-156.254	< .001***
	THSO 2.5	-2.190	-2.254	-2.126	0.017	42	-130.113	< .001***
	AHSO 5	-2.850	-2.914	-2.786	0.017	42	-169.325	< .001***
	SHSO 5	-3.070	-3.134	-3.006	0.017	42	-182.395	< .001***
	THSO 5	-8.650	-8.714	-8.586	0.017	42	-513.915	< .001***
	AHSO 9	-2.960	-3.024	-2.896	0.017	42	-175.860	< .001***
	SHSO 9	-4.270	-4.334	-4.206	0.017	42	-253.690	< .001***

Post Hoc Comparisons - sample ✻ concentration

		Mean Difference	95% CI for Mean Difference		SE	df	t	p_{tukey}
			Lower	Upper				
	THSO 9	-10.630	-10.694	-10.566	0.017	42	-631.551	< .001***
	AHSO 16	-3.730	-3.794	-3.666	0.017	42	-221.607	< .001***
	SHSO 16	-8.440	-8.504	-8.376	0.017	42	-501.439	< .001***
	THSO 16	-32.970	-33.034	-32.906	0.017	42	-1958.820	< .001***
THSO 0.6	AHSO 1.3	-4.820	-4.884	-4.756	0.017	42	-286.367	< .001***
	SHSO 1.3	-3.620	-3.684	-3.556	0.017	42	-215.072	< .001***
	THSO 1.3	-3.840	-3.904	-3.776	0.017	42	-228.143	< .001***
	AHSO 2.5	-5.150	-5.214	-5.086	0.017	42	-305.973	< .001***
	SHSO 2.5	-5.040	-5.104	-4.976	0.017	42	-299.437	< .001***
	THSO 2.5	-4.600	-4.664	-4.536	0.017	42	-273.296	< .001***
	AHSO 5	-5.260	-5.324	-5.196	0.017	42	-312.508	< .001***
	SHSO 5	-5.480	-5.544	-5.416	0.017	42	-325.579	< .001***
	THSO 5	-11.060	-11.124	-10.996	0.017	42	-657.099	< .001***
	AHSO 9	-5.370	-5.434	-5.306	0.017	42	-319.043	< .001***
	SHSO 9	-6.680	-6.744	-6.616	0.017	42	-396.873	< .001***
	THSO 9	-13.040	-13.104	-12.976	0.017	42	-774.735	< .001***
	AHSO 16	-6.140	-6.204	-6.076	0.017	42	-364.791	< .001***
	SHSO 16	-10.850	-10.914	-10.786	0.017	42	-644.622	< .001***
	THSO 16	-35.380	-35.444	-35.316	0.017	42	-2102.003	< .001***
AHSO 1.3	SHSO 1.3	1.200	1.136	1.264	0.017	42	71.295	< .001***
	THSO 1.3	0.980	0.916	1.044	0.017	42	58.224	< .001***
	AHSO 2.5	-0.330	-0.394	-0.266	0.017	42	-19.606	< .001***
	SHSO 2.5	-0.220	-0.284	-0.156	0.017	42	-13.071	< .001***
	THSO 2.5	0.220	0.156	0.284	0.017	42	13.071	< .001***
	AHSO 5	-0.440	-0.504	-0.376	0.017	42	-26.141	< .001***
	SHSO 5	-0.660	-0.724	-0.596	0.017	42	-39.212	< .001***

Post Hoc Comparisons - sample ✻ concentration

		Mean Difference	95% CI for Mean Difference		SE	df	t	p_{tukey}
			Lower	Upper				
	THSO 5	-6.240	-6.304	-6.176	0.017	42	-370.732	< .001***
	AHSO 9	-0.550	-0.614	-0.486	0.017	42	-32.677	< .001***
	SHSO 9	-1.860	-1.924	-1.796	0.017	42	-110.507	< .001***
	THSO 9	-8.220	-8.284	-8.156	0.017	42	-488.368	< .001***
	AHSO 16	-1.320	-1.384	-1.256	0.017	42	-78.424	< .001***
	SHSO 16	-6.030	-6.094	-5.966	0.017	42	-358.255	< .001***
	THSO 16	-30.560	-30.624	-30.496	0.017	42	-1815.636	< .001***
SHSO 1.3	THSO 1.3	-0.220	-0.284	-0.156	0.017	42	-13.071	< .001***
	AHSO 2.5	-1.530	-1.594	-1.466	0.017	42	-90.901	< .001***
	SHSO 2.5	-1.420	-1.484	-1.356	0.017	42	-84.365	< .001***
	THSO 2.5	-0.980	-1.044	-0.916	0.017	42	-58.224	< .001***
	AHSO 5	-1.640	-1.704	-1.576	0.017	42	-97.436	< .001***
	SHSO 5	-1.860	-1.924	-1.796	0.017	42	-110.507	< .001***
	THSO 5	-7.440	-7.504	-7.376	0.017	42	-442.027	< .001***
	AHSO 9	-1.750	-1.814	-1.686	0.017	42	-103.971	< .001***
	SHSO 9	-3.060	-3.124	-2.996	0.017	42	-181.801	< .001***
	THSO 9	-9.420	-9.484	-9.356	0.017	42	-559.663	< .001***
	AHSO 16	-2.520	-2.584	-2.456	0.017	42	-149.719	< .001***
	SHSO 16	-7.230	-7.294	-7.166	0.017	42	-429.550	< .001***
	THSO 16	-31.760	-31.824	-31.696	0.017	42	-1886.931	< .001***
THSO 1.3	AHSO 2.5	-1.310	-1.374	-1.246	0.017	42	-77.830	< .001***
	SHSO 2.5	-1.200	-1.264	-1.136	0.017	42	-71.295	< .001***
	THSO 2.5	-0.760	-0.824	-0.696	0.017	42	-45.153	< .001***
	AHSO 5	-1.420	-1.484	-1.356	0.017	42	-84.365	< .001***
	SHSO 5	-1.640	-1.704	-1.576	0.017	42	-97.436	< .001***
	THSO 5	-7.220	-7.284	-7.156	0.017	42	-428.956	< .001***
	AHSO 9	-1.530	-1.594	-1.466	0.017	42	-90.901	< .001***
	SHSO 9	-2.840	-2.904	-2.776	0.017	42	-168.731	< .001***
	THSO 9	-9.200	-9.264	-9.136	0.017	42	-546.592	< .001***

Post Hoc Comparisons - sample ✻ concentration

		Mean Difference	95% CI for Mean Difference Lower	Upper	SE	df	t	p_{tukey}
	AHSO 16	-2.300	-2.364	-2.236	0.017	42	-136.648	< .001***
	SHSO 16	-7.010	-7.074	-6.946	0.017	42	-416.479	< .001***
	THSO 16	-31.540	-31.604	-31.476	0.017	42	-1873.860	< .001***
AHSO 2.5	SHSO 2.5	0.110	0.046	0.174	0.017	42	6.535	< .001***
	THSO 2.5	0.550	0.486	0.614	0.017	42	32.677	< .001***
	AHSO 5	-0.110	-0.174	-0.046	0.017	42	-6.535	< .001***
	SHSO 5	-0.330	-0.394	-0.266	0.017	42	-19.606	< .001***
	THSO 5	-5.910	-5.974	-5.846	0.017	42	-351.126	< .001***
	AHSO 9	-0.220	-0.284	-0.156	0.017	42	-13.071	< .001***
	SHSO 9	-1.530	-1.594	-1.466	0.017	42	-90.901	< .001***
	THSO 9	-7.890	-7.954	-7.826	0.017	42	-468.762	< .001***
	AHSO 16	-0.990	-1.054	-0.926	0.017	42	-58.818	< .001***
	SHSO 16	-5.700	-5.764	-5.636	0.017	42	-338.649	< .001***
	THSO 16	-30.230	-30.294	-30.166	0.017	42	-1796.030	< .001***
SHSO 2.5	THSO 2.5	0.440	0.376	0.504	0.017	42	26.141	< .001***
	AHSO 5	-0.220	-0.284	-0.156	0.017	42	-13.071	< .001***
	SHSO 5	-0.440	-0.504	-0.376	0.017	42	-26.141	< .001***
	THSO 5	-6.020	-6.084	-5.956	0.017	42	-357.661	< .001***
	AHSO 9	-0.330	-0.394	-0.266	0.017	42	-19.606	< .001***
	SHSO 9	-1.640	-1.704	-1.576	0.017	42	-97.436	< .001***
	THSO 9	-8.000	-8.064	-7.936	0.017	42	-475.297	< .001***
	AHSO 16	-1.100	-1.164	-1.036	0.017	42	-65.353	< .001***
	SHSO 16	-5.810	-5.874	-5.746	0.017	42	-345.185	< .001***
	THSO 16	-30.340	-30.404	-30.276	0.017	42	-1802.566	< .001***
THSO 2.5	AHSO 5	-0.660	-0.724	-0.596	0.017	42	-39.212	< .001***
	SHSO 5	-0.880	-0.944	-0.816	0.017	42	-52.283	< .001***
	THSO 5	-6.460	-6.524	-6.396	0.017	42	-383.803	< .001***
	AHSO 9	-0.770	-0.834	-0.706	0.017	42	-45.747	< .001***
	SHSO 9	-2.080	-2.144	-2.016	0.017	42	-123.577	< .001***
	THSO 9	-8.440	-8.504	-8.376	0.017	42	-501.439	< .001***

Post Hoc Comparisons - sample ✻ concentration

		Mean Difference	95% CI for Mean Difference		SE	df	t	p_{tukey}
			Lower	Upper				
	AHSO 16	-1.540	-1.604	-1.476	0.017	42	-91.495	< .001***
	SHSO 16	-6.250	-6.314	-6.186	0.017	42	-371.326	< .001***
	THSO 16	-30.780	-30.844	-30.716	0.017	42	-1828.707	< .001***
AHSO 5	SHSO 5	-0.220	-0.284	-0.156	0.017	42	-13.071	< .001***
	THSO 5	-5.800	-5.864	-5.736	0.017	42	-344.591	< .001***
	AHSO 9	-0.110	-0.174	-0.046	0.017	42	-6.535	< .001***
	SHSO 9	-1.420	-1.484	-1.356	0.017	42	-84.365	< .001***
	THSO 9	-7.780	-7.844	-7.716	0.017	42	-462.227	< .001***
	AHSO 16	-0.880	-0.944	-0.816	0.017	42	-52.283	< .001***
	SHSO 16	-5.590	-5.654	-5.526	0.017	42	-332.114	< .001***
	THSO 16	-30.120	-30.184	-30.056	0.017	42	-1789.495	< .001***
SHSO 5	THSO 5	-5.580	-5.644	-5.516	0.017	42	-331.520	< .001***
	AHSO 9	0.110	0.046	0.174	0.017	42	6.535	< .001***
	SHSO 9	-1.200	-1.264	-1.136	0.017	42	-71.295	< .001***
	THSO 9	-7.560	-7.624	-7.496	0.017	42	-449.156	< .001***
	AHSO 16	-0.660	-0.724	-0.596	0.017	42	-39.212	< .001***
	SHSO 16	-5.370	-5.434	-5.306	0.017	42	-319.043	< .001***
	THSO 16	-29.900	-29.964	-29.836	0.017	42	-1776.424	< .001***
THSO 5	AHSO 9	5.690	5.626	5.754	0.017	42	338.055	< .001***
	SHSO 9	4.380	4.316	4.444	0.017	42	260.225	< .001***
	THSO 9	-1.980	-2.044	-1.916	0.017	42	-117.636	< .001***
	AHSO 16	4.920	4.856	4.984	0.017	42	292.308	< .001***
	SHSO 16	0.210	0.146	0.274	0.017	42	12.477	< .001***
	THSO 16	-24.320	-24.384	-24.256	0.017	42	-1444.904	< .001***
AHSO 9	SHSO 9	-1.310	-1.374	-1.246	0.017	42	-77.830	< .001***
	THSO 9	-7.670	-7.734	-7.606	0.017	42	-455.691	< .001***
	AHSO 16	-0.770	-0.834	-0.706	0.017	42	-45.747	< .001***
	SHSO 16	-5.480	-5.544	-5.416	0.017	42	-325.579	< .001***

Post Hoc Comparisons - sample ✻ concentration

		Mean Difference	95% CI for Mean Difference		SE	df	t	p_{tukey}
			Lower	Upper				
	THSO 16	-30.010	-30.074	-29.946	0.017	42	-1782.960	< .001***
SHSO 9	THSO 9	-6.360	-6.424	-6.296	0.017	42	-377.861	< .001***
	AHSO 16	0.540	0.476	0.604	0.017	42	32.083	< .001***
	SHSO 16	-4.170	-4.234	-4.106	0.017	42	-247.749	< .001***
	THSO 16	-28.700	-28.764	-28.636	0.017	42	-1705.130	< .001***
THSO 9	AHSO 16	6.900	6.836	6.964	0.017	42	409.944	< .001***
	SHSO 16	2.190	2.126	2.254	0.017	42	130.113	< .001***
	THSO 16	-22.340	-22.404	-22.276	0.017	42	-1327.268	< .001***
AHSO 16	SHSO 16	-4.710	-4.774	-4.646	0.017	42	-279.831	< .001***
	THSO 16	-29.240	-29.304	-29.176	0.017	42	-1737.212	< .001***
SHSO 16	THSO 16	-24.530	-24.594	-24.466	0.017	42	-1457.381	< .001***

*** $p < .001$

Note. P-value and confidence intervals adjusted for comparing a family of 21 estimates (confidence intervals corrected using the tukey method).

Letter-Based Grouping - sample ✻ concentration

sample	concentration	Letter
AHSO	0.3	b
SHSO		c
THSO		a
AHSO	0.6	g
SHSO		e
THSO		d
AHSO	1.3	i
SHSO		f
THSO		g
AHSO	2.5	k
SHSO		j
THSO		h
AHSO	5	l
SHSO		n

Letter-Based Grouping - sample ✻ concentration

sample	concentration	Letter
THSO		r
AHSO	9	m
SHSO		p
THSO		s
AHSO	16	o
SHSO		q
THSO		t

Note. If two or more means share the same grouping symbol, then we cannot show them to be different, but we also did not show them to be the same.

ANOVA

ANOVA - Lactobacillus acidophilus ATCC 4356

Cases	Sum of Squares	df	Mean Square	F	p
sample	2946.707	2	1473.354	$4.178\times10^{+6}$	< .001
concentration	5309.650	6	884.942	$2.510\times10^{+6}$	< .001
sample ✻ concentration	2850.463	12	237.539	673640.785	< .001
Residuals	0.015	42	3.526×10^{-4}		

Note. Type III Sum of Squares

Descriptives

Descriptives - Lactobacillus acidophilus ATCC 4356

sample	concentration	N	Mean	SD	SE	Coefficient of variation
AHSO	0.3	3	-10.950	0.024	0.014	-0.002
	0.6	3	-5.840	0.025	0.014	-0.004
	1.3	3	-5.110	0.013	0.007	-0.002
	2.5	3	-3.650	0.015	0.008	-0.004
	5	3	0.730	0.014	0.008	0.019
	9	3	1.460	0.015	0.009	0.010
	16	3	2.920	0.019	0.011	0.007
SHSO	0.3	3	-11.680	0.027	0.016	-0.002
	0.6	3	-0.730	0.011	0.006	-0.015
	1.3	3	2.190	0.012	0.007	0.005
	2.5	3	2.190	0.014	0.008	0.006
	5	3	2.920	0.029	0.017	0.010
	9	3	9.490	0.023	0.013	0.002
	16	3	10.950	0.020	0.012	0.002
THSO	0.3	3	-4.380	0.017	0.010	-0.004

Descriptives - Lactobacillus acidophilus ATCC 4356

sample	concentration	N	Mean	SD	SE	Coefficient of variation
	0.6	3	1.460	0.021	0.012	0.014
	1.3	3	6.570	0.018	0.010	0.003
	2.5	3	8.030	0.016	0.009	0.002
	5	3	13.870	0.013	0.008	9.373×10^{-4}
	9	3	13.870	0.022	0.013	0.002
	16	3	54.740	0.012	0.007	2.101×10^{-4}

Post Hoc Tests

Standard (HSD)

Post Hoc Comparisons - sample ✻ concentration

			95% CI for Mean Difference					
		Mean Difference	Lower	Upper	SE	df	t	p_{tukey}
AHSO 0.3	SHSO 0.3	0.730	0.672	0.788	0.015	42	47.612	< .001***
	THSO 0.3	-6.570	-6.628	-6.512	0.015	42	-428.507	< .001***
	AHSO 0.6	-5.110	-5.168	-5.052	0.015	42	-333.283	< .001***
	SHSO 0.6	-10.220	-10.278	-10.162	0.015	42	-666.567	< .001***
	THSO 0.6	-12.410	-12.468	-12.352	0.015	42	-809.402	< .001***
	AHSO 1.3	-5.840	-5.898	-5.781	0.015	42	-380.874	< .001***
	SHSO 1.3	-13.140	-13.198	-13.082	0.015	42	-857.014	< .001***
	THSO 1.3	-17.520	-17.578	-17.462	0.015	42	-1142.686	< .001***
	AHSO 2.5	-7.300	-7.359	-7.242	0.015	42	-476.141	< .001***
	SHSO 2.5	-13.140	-13.198	-13.082	0.015	42	-857.014	< .001***
	THSO 2.5	-18.980	-19.038	-18.922	0.015	42	-1237.910	< .001***
	AHSO 5	-11.680	-11.738	-11.622	0.015	42	-761.791	< .001***
	SHSO 5	-13.870	-13.928	-13.812	0.015	42	-904.626	< .001***
	THSO 5	-24.820	-24.878	-24.762	0.015	42	-1618.805	< .001***
	AHSO 9	-12.410	-12.468	-12.352	0.015	42	-809.402	< .001***
	SHSO 9	-20.440	-20.498	-20.382	0.015	42	-1333.133	< .001***
	THSO 9	-24.820	-24.878	-24.762	0.015	42	-1618.805	< .001***

Post Hoc Comparisons - sample ✻ concentration

		Mean Difference	95% CI for Mean Difference		SE	df	t	p_{tukey}
			Lower	Upper				
	AHSO 16	-13.870	-13.928	-13.812	0.015	42	-904.626	< .001***
	SHSO 16	-21.900	-21.958	-21.842	0.015	42	-1428.357	< .001***
	THSO 16	-65.690	-65.749	-65.632	0.015	42	-4284.441	< .001***
SHSO 0.3	THSO 0.3	-7.300	-7.358	-7.242	0.015	42	-476.119	< .001***
	AHSO 0.6	-5.840	-5.898	-5.782	0.015	42	-380.895	< .001***
	SHSO 0.6	-10.950	-11.008	-10.892	0.015	42	-714.179	< .001***
	THSO 0.6	-13.140	-13.198	-13.082	0.015	42	-857.014	< .001***
	AHSO 1.3	-6.570	-6.628	-6.511	0.015	42	-428.485	< .001***
	SHSO 1.3	-13.870	-13.928	-13.812	0.015	42	-904.626	< .001***
	THSO 1.3	-18.250	-18.308	-18.192	0.015	42	-1190.298	< .001***
	AHSO 2.5	-8.030	-8.089	-7.972	0.015	42	-523.753	< .001***
	SHSO 2.5	-13.870	-13.928	-13.812	0.015	42	-904.626	< .001***
	THSO 2.5	-19.710	-19.768	-19.652	0.015	42	-1285.522	< .001***
	AHSO 5	-12.410	-12.468	-12.352	0.015	42	-809.402	< .001***
	SHSO 5	-14.600	-14.658	-14.542	0.015	42	-952.238	< .001***
	THSO 5	-25.550	-25.608	-25.492	0.015	42	-1666.417	< .001***
	AHSO 9	-13.140	-13.198	-13.082	0.015	42	-857.014	< .001***
	SHSO 9	-21.170	-21.228	-21.112	0.015	42	-1380.745	< .001***
	THSO 9	-25.550	-25.608	-25.492	0.015	42	-1666.417	< .001***
	AHSO 16	-14.600	-14.658	-14.542	0.015	42	-952.238	< .001***
	SHSO 16	-22.630	-22.688	-22.572	0.015	42	-1475.969	< .001***
	THSO 16	-66.420	-66.479	-66.362	0.015	42	-4332.053	< .001***
THSO 0.3	AHSO 0.6	1.460	1.402	1.518	0.015	42	95.224	< .001***
	SHSO 0.6	-3.650	-3.708	-3.592	0.015	42	-238.060	< .001***

Post Hoc Comparisons - sample ✲ concentration

		Mean Difference	95% CI for Mean Difference		SE	df	t	p_{tukey}
			Lower	Upper				
	THSO 0.6	-5.840	-5.898	-5.782	0.015	42	-380.895	< .001***
	AHSO 1.3	0.730	0.672	0.789	0.015	42	47.634	< .001***
	SHSO 1.3	-6.570	-6.628	-6.512	0.015	42	-428.507	< .001***
	THSO 1.3	-10.950	-11.008	-10.892	0.015	42	-714.179	< .001***
	AHSO 2.5	-0.730	-0.789	-0.672	0.015	42	-47.634	< .001***
	SHSO 2.5	-6.570	-6.628	-6.512	0.015	42	-428.507	< .001***
	THSO 2.5	-12.410	-12.468	-12.352	0.015	42	-809.402	< .001***
	AHSO 5	-5.110	-5.168	-5.052	0.015	42	-333.283	< .001***
	SHSO 5	-7.300	-7.358	-7.242	0.015	42	-476.119	< .001***
	THSO 5	-18.250	-18.308	-18.192	0.015	42	-1190.298	< .001***
	AHSO 9	-5.840	-5.898	-5.782	0.015	42	-380.895	< .001***
	SHSO 9	-13.870	-13.928	-13.812	0.015	42	-904.626	< .001***
	THSO 9	-18.250	-18.308	-18.192	0.015	42	-1190.298	< .001***
	AHSO 16	-7.300	-7.358	-7.242	0.015	42	-476.119	< .001***
	SHSO 16	-15.330	-15.388	-15.272	0.015	42	-999.850	< .001***
	THSO 16	-59.120	-59.179	-59.062	0.015	42	-3855.934	< .001***
AHSO 0.6	SHSO 0.6	-5.110	-5.168	-5.052	0.015	42	-333.283	< .001***
	THSO 0.6	-7.300	-7.358	-7.242	0.015	42	-476.119	< .001***
	AHSO 1.3	-0.730	-0.788	-0.671	0.015	42	-47.590	< .001***
	SHSO 1.3	-8.030	-8.088	-7.972	0.015	42	-523.731	< .001***
	THSO 1.3	-12.410	-12.468	-12.352	0.015	42	-809.402	< .001***
	AHSO 2.5	-2.190	-2.249	-2.132	0.015	42	-142.857	< .001***
	SHSO 2.5	-8.030	-8.088	-7.972	0.015	42	-523.731	< .001***
	THSO 2.5	-13.870	-13.928	-13.812	0.015	42	-904.626	< .001***
	AHSO 5	-6.570	-6.628	-6.512	0.015	42	-428.507	< .001***

Post Hoc Comparisons - sample ✻ concentration

		Mean Difference	95% CI for Mean Difference		SE	df	t	p_{tukey}
			Lower	Upper				
	SHSO 5	-8.760	-8.818	-8.702	0.015	42	-571.343	< .001***
	THSO 5	-19.710	-19.768	-19.652	0.015	42	-1285.522	< .001***
	AHSO 9	-7.300	-7.358	-7.242	0.015	42	-476.119	< .001***
	SHSO 9	-15.330	-15.388	-15.272	0.015	42	-999.850	< .001***
	THSO 9	-19.710	-19.768	-19.652	0.015	42	-1285.522	< .001***
	AHSO 16	-8.760	-8.818	-8.702	0.015	42	-571.343	< .001***
	SHSO 16	-16.790	-16.848	-16.732	0.015	42	-1095.074	< .001***
	THSO 16	-60.580	-60.639	-60.522	0.015	42	-3951.158	< .001***
SHSO 0.6	THSO 0.6	-2.190	-2.248	-2.132	0.015	42	-142.836	< .001***
	AHSO 1.3	4.380	4.322	4.439	0.015	42	285.693	< .001***
	SHSO 1.3	-2.920	-2.978	-2.862	0.015	42	-190.448	< .001***
	THSO 1.3	-7.300	-7.358	-7.242	0.015	42	-476.119	< .001***
	AHSO 2.5	2.920	2.861	2.978	0.015	42	190.426	< .001***
	SHSO 2.5	-2.920	-2.978	-2.862	0.015	42	-190.448	< .001***
	THSO 2.5	-8.760	-8.818	-8.702	0.015	42	-571.343	< .001***
	AHSO 5	-1.460	-1.518	-1.402	0.015	42	-95.224	< .001***
	SHSO 5	-3.650	-3.708	-3.592	0.015	42	-238.060	< .001***
	THSO 5	-14.600	-14.658	-14.542	0.015	42	-952.238	< .001***
	AHSO 9	-2.190	-2.248	-2.132	0.015	42	-142.836	< .001***
	SHSO 9	-10.220	-10.278	-10.162	0.015	42	-666.567	< .001***
	THSO 9	-14.600	-14.658	-14.542	0.015	42	-952.238	< .001***
	AHSO 16	-3.650	-3.708	-3.592	0.015	42	-238.060	< .001***
	SHSO 16	-11.680	-11.738	-11.622	0.015	42	-761.791	< .001***
	THSO 16	-55.470	-55.529	-55.412	0.015	42	-3617.875	< .001***
THSO 0.6	AHSO 1.3	6.570	6.512	6.629	0.015	42	428.529	< .001***
	SHSO 1.3	-0.730	-0.788	-0.672	0.015	42	-47.612	< .001***
	THSO 1.3	-5.110	-5.168	-5.052	0.015	42	-333.283	< .001***

Post Hoc Comparisons - sample ✻ concentration

		Mean Difference	95% CI for Mean Difference Lower	95% CI for Mean Difference Upper	SE	df	t	p_{tukey}
	AHSO 2.5	5.110	5.051	5.168	0.015	42	333.262	< .001***
	SHSO 2.5	-0.730	-0.788	-0.672	0.015	42	-47.612	< .001***
	THSO 2.5	-6.570	-6.628	-6.512	0.015	42	-428.507	< .001***
	AHSO 5	0.730	0.672	0.788	0.015	42	47.612	< .001***
	SHSO 5	-1.460	-1.518	-1.402	0.015	42	-95.224	< .001***
	THSO 5	-12.410	-12.468	-12.352	0.015	42	-809.402	< .001***
	AHSO 9	-7.494×10^{-15}	-0.058	0.058	0.015	42	-4.888×10^{-13}	1.000
	SHSO 9	-8.030	-8.088	-7.972	0.015	42	-523.731	< .001***
	THSO 9	-12.410	-12.468	-12.352	0.015	42	-809.402	< .001***
	AHSO 16	-1.460	-1.518	-1.402	0.015	42	-95.224	< .001***
	SHSO 16	-9.490	-9.548	-9.432	0.015	42	-618.955	< .001***
	THSO 16	-53.280	-53.339	-53.222	0.015	42	-3475.039	< .001***
AHSO 1.3	SHSO 1.3	-7.300	-7.359	-7.242	0.015	42	-476.141	< .001***
	THSO 1.3	-11.680	-11.739	-11.622	0.015	42	-761.812	< .001***
	AHSO 2.5	-1.461	-1.519	-1.402	0.015	42	-95.267	< .001***
	SHSO 2.5	-7.300	-7.359	-7.242	0.015	42	-476.141	< .001***
	THSO 2.5	-13.140	-13.199	-13.082	0.015	42	-857.036	< .001***
	AHSO 5	-5.840	-5.899	-5.782	0.015	42	-380.917	< .001***
	SHSO 5	-8.030	-8.089	-7.972	0.015	42	-523.753	< .001***
	THSO 5	-18.980	-19.039	-18.922	0.015	42	-1237.931	< .001***
	AHSO 9	-6.570	-6.629	-6.512	0.015	42	-428.529	< .001***
	SHSO 9	-14.600	-14.659	-14.542	0.015	42	-952.260	< .001***
	THSO 9	-18.980	-19.039	-18.922	0.015	42	-1237.931	< .001***
	AHSO 16	-8.030	-8.089	-7.972	0.015	42	-523.753	< .001***
	SHSO 16	-16.060	-16.119	-16.002	0.015	42	-1047.484	< .001***
	THSO 16	-59.851	-59.909	-59.792	0.015	42	-3903.568	< .001***
SHSO 1.3	THSO 1.3	-4.380	-4.438	-4.322	0.015	42	-285.671	< .001***

Post Hoc Comparisons - sample ✻ concentration

		Mean Difference	95% CI for Mean Difference Lower	95% CI for Mean Difference Upper	SE	df	t	p_{tukey}
	AHSO 2.5	5.840	5.781	5.898	0.015	42	380.874	< .001***
	SHSO 2.5	-7.550×10^{-15}	-0.058	0.058	0.015	42	-4.924×10^{-13}	1.000
	THSO 2.5	-5.840	-5.898	-5.782	0.015	42	-380.895	< .001***
	AHSO 5	1.460	1.402	1.518	0.015	42	95.224	< .001***
	SHSO 5	-0.730	-0.788	-0.672	0.015	42	-47.612	< .001***
	THSO 5	-11.680	-11.738	-11.622	0.015	42	-761.791	< .001***
	AHSO 9	0.730	0.672	0.788	0.015	42	47.612	< .001***
	SHSO 9	-7.300	-7.358	-7.242	0.015	42	-476.119	< .001***
	THSO 9	-11.680	-11.738	-11.622	0.015	42	-761.791	< .001***
	AHSO 16	-0.730	-0.788	-0.672	0.015	42	-47.612	< .001***
	SHSO 16	-8.760	-8.818	-8.702	0.015	42	-571.343	< .001***
	THSO 16	-52.550	-52.609	-52.492	0.015	42	-3427.427	< .001***
THSO 1.3	AHSO 2.5	10.220	10.161	10.278	0.015	42	666.545	< .001***
	SHSO 2.5	4.380	4.322	4.438	0.015	42	285.671	< .001***
	THSO 2.5	-1.460	-1.518	-1.402	0.015	42	-95.224	< .001***
	AHSO 5	5.840	5.782	5.898	0.015	42	380.895	< .001***
	SHSO 5	3.650	3.592	3.708	0.015	42	238.060	< .001***
	THSO 5	-7.300	-7.358	-7.242	0.015	42	-476.119	< .001***
	AHSO 9	5.110	5.052	5.168	0.015	42	333.283	< .001***
	SHSO 9	-2.920	-2.978	-2.862	0.015	42	-190.448	< .001***
	THSO 9	-7.300	-7.358	-7.242	0.015	42	-476.119	< .001***
	AHSO 16	3.650	3.592	3.708	0.015	42	238.060	< .001***
	SHSO 16	-4.380	-4.438	-4.322	0.015	42	-285.671	< .001***
	THSO 16	-48.170	-48.229	-48.112	0.015	42	-3141.756	< .001***
AHSO 2.5	SHSO 2.5	-5.840	-5.898	-5.781	0.015	42	-380.874	< .001***
	THSO 2.5	-11.680	-11.738	-11.621	0.015	42	-761.769	< .001***
	AHSO 5	-4.380	-4.438	-4.321	0.015	42	-285.650	< .001***
	SHSO 5	-6.570	-6.628	-6.511	0.015	42	-428.485	< .001***
	THSO 5	-17.520	-17.578	-17.461	0.015	42	-1142.664	< .001***

Post Hoc Comparisons - sample ✻ concentration

		Mean Difference	95% CI for Mean Difference		SE	df	t	p_{tukey}
			Lower	Upper				
	AHSO 9	-5.110	-5.168	-5.051	0.015	42	-333.262	< .001***
	SHSO 9	-13.140	-13.198	-13.081	0.015	42	-856.993	< .001***
	THSO 9	-17.520	-17.578	-17.461	0.015	42	-1142.664	< .001***
	AHSO 16	-6.570	-6.628	-6.511	0.015	42	-428.485	< .001***
	SHSO 16	-14.600	-14.658	-14.541	0.015	42	-952.216	< .001***
	THSO 16	-58.390	-58.448	-58.332	0.015	42	-3808.301	< .001***
SHSO 2.5	THSO 2.5	-5.840	-5.898	-5.782	0.015	42	-380.895	< .001***
	AHSO 5	1.460	1.402	1.518	0.015	42	95.224	< .001***
	SHSO 5	-0.730	-0.788	-0.672	0.015	42	-47.612	< .001***
	THSO 5	-11.680	-11.738	-11.622	0.015	42	-761.791	< .001***
	AHSO 9	0.730	0.672	0.788	0.015	42	47.612	< .001***
	SHSO 9	-7.300	-7.358	-7.242	0.015	42	-476.119	< .001***
	THSO 9	-11.680	-11.738	-11.622	0.015	42	-761.791	< .001***
	AHSO 16	-0.730	-0.788	-0.672	0.015	42	-47.612	< .001***
	SHSO 16	-8.760	-8.818	-8.702	0.015	42	-571.343	< .001***
	THSO 16	-52.550	-52.609	-52.492	0.015	42	-3427.427	< .001***
THSO 2.5	AHSO 5	7.300	7.242	7.358	0.015	42	476.119	< .001***
	SHSO 5	5.110	5.052	5.168	0.015	42	333.283	< .001***
	THSO 5	-5.840	-5.898	-5.782	0.015	42	-380.895	< .001***
	AHSO 9	6.570	6.512	6.628	0.015	42	428.507	< .001***
	SHSO 9	-1.460	-1.518	-1.402	0.015	42	-95.224	< .001***
	THSO 9	-5.840	-5.898	-5.782	0.015	42	-380.895	< .001***
	AHSO 16	5.110	5.052	5.168	0.015	42	333.283	< .001***
	SHSO 16	-2.920	-2.978	-2.862	0.015	42	-190.448	< .001***
	THSO 16	-46.710	-46.769	-46.652	0.015	42	-3046.532	< .001***
AHSO 5	SHSO 5	-2.190	-2.248	-2.132	0.015	42	-142.836	< .001***
	THSO 5	-13.140	-13.198	-13.082	0.015	42	-857.014	< .001***
	AHSO 9	-0.730	-0.788	-0.672	0.015	42	-47.612	< .001***
	SHSO 9	-8.760	-8.818	-8.702	0.015	42	-571.343	< .001***
	THSO 9	-13.140	-13.198	-13.082	0.015	42	-857.014	< .001***
	AHSO 16	-2.190	-2.248	-2.132	0.015	42	-142.836	< .001***

Post Hoc Comparisons - sample ✻ concentration

		Mean Difference	95% CI for Mean Difference		SE	df	t	p_{tukey}
			Lower	Upper				
	SHSO 16	-10.220	-10.278	-10.162	0.015	42	-666.567	< .001***
	THSO 16	-54.010	-54.069	-53.952	0.015	42	-3522.651	< .001***
SHSO 5	THSO 5	-10.950	-11.008	-10.892	0.015	42	-714.179	< .001***
	AHSO 9	1.460	1.402	1.518	0.015	42	95.224	< .001***
	SHSO 9	-6.570	-6.628	-6.512	0.015	42	-428.507	< .001***
	THSO 9	-10.950	-11.008	-10.892	0.015	42	-714.179	< .001***
	AHSO 16	3.941×10^{-15}	-0.058	0.058	0.015	42	2.571×10^{-13}	1.000
	SHSO 16	-8.030	-8.088	-7.972	0.015	42	-523.731	< .001***
	THSO 16	-51.820	-51.879	-51.762	0.015	42	-3379.815	< .001***
THSO 5	AHSO 9	12.410	12.352	12.468	0.015	42	809.402	< .001***
	SHSO 9	4.380	4.322	4.438	0.015	42	285.671	< .001***
	THSO 9	-1.610×10^{-15}	-0.058	0.058	0.015	42	-1.050×10^{-13}	1.000
	AHSO 16	10.950	10.892	11.008	0.015	42	714.179	< .001***
	SHSO 16	2.920	2.862	2.978	0.015	42	190.448	< .001***
	THSO 16	-40.870	-40.929	-40.812	0.015	42	-2665.636	< .001***
AHSO 9	SHSO 9	-8.030	-8.088	-7.972	0.015	42	-523.731	< .001***
	THSO 9	-12.410	-12.468	-12.352	0.015	42	-809.402	< .001***
	AHSO 16	-1.460	-1.518	-1.402	0.015	42	-95.224	< .001***
	SHSO 16	-9.490	-9.548	-9.432	0.015	42	-618.955	< .001***
	THSO 16	-53.280	-53.339	-53.222	0.015	42	-3475.039	< .001***
SHSO 9	THSO 9	-4.380	-4.438	-4.322	0.015	42	-285.671	< .001***
	AHSO 16	6.570	6.512	6.628	0.015	42	428.507	< .001***
	SHSO 16	-1.460	-1.518	-1.402	0.015	42	-95.224	< .001***
	THSO 16	-45.250	-45.309	-45.192	0.015	42	-2951.308	< .001***
THSO 9	AHSO 16	10.950	10.892	11.008	0.015	42	714.179	< .001***
	SHSO 16	2.920	2.862	2.978	0.015	42	190.448	< .001***

Post Hoc Comparisons - sample ✻ concentration

		Mean Difference	95% CI for Mean Difference Lower	95% CI for Mean Difference Upper	SE	df	t	p_{tukey}
AHSO 16	THSO 16	-40.870	-40.929	-40.812	0.015	42	-2665.636	< .001***
	SHSO 16	-8.030	-8.088	-7.972	0.015	42	-523.731	< .001***
	THSO 16	-51.820	-51.879	-51.762	0.015	42	-3379.815	< .001***
SHSO 16	THSO 16	-43.790	-43.849	-43.732	0.015	42	-2856.084	< .001***

*** p < .001

Note. P-value and confidence intervals adjusted for comparing a family of 21 estimates (confidence intervals corrected using the tukey method).

Letter-Based Grouping - sample ✻ concentration

sample	concentration	Letter
AHSO	0.3	b
SHSO		a
THSO		e
AHSO	0.6	c
SHSO		g
THSO		i
AHSO	1.3	d
SHSO		j
THSO		l
AHSO	2.5	f
SHSO		j
THSO		m
AHSO	5	h
SHSO		k
THSO		p
AHSO	9	i
SHSO		n
THSO		p
AHSO	16	k
SHSO		o
THSO		q

Note. If two or more means share the same grouping symbol, then we cannot show them to be different, but we also did not show them to be the same.

ANOVA

ANOVA - Lactocaseibacillus rhamnosus (GG; HN001)

Cases	Sum of Squares	df	Mean Square	F	p
sample	0.002	2	9.571×10^{-4}	2.264	0.116
concentration	1.006	6	0.168	396.531	< .001
sample ✻ concentration	0.211	12	0.018	41.525	< .001
Residuals	0.018	42	4.228×10^{-4}		

Note. Type III Sum of Squares

Descriptives

Descriptives - Lactocaseibacillus rhamnosus (GG; HN001)

sample	concentration	N	Mean	SD	SE	Coefficient of variation
AHSO	0.3	3	0.740	0.028	0.016	0.038
	0.6	3	0.850	0.027	0.016	0.032
	1.3	3	0.860	0.012	0.007	0.014
	2.5	3	0.880	0.017	0.010	0.019
	5	3	0.880	0.025	0.014	0.028
	9	3	0.890	0.026	0.015	0.029
	16	3	0.930	0.015	0.009	0.016
SHSO	0.3	3	0.490	0.015	0.009	0.031
	0.6	3	0.830	0.013	0.008	0.016
	1.3	3	0.860	0.024	0.014	0.028
	2.5	3	0.870	0.014	0.008	0.016
	5	3	0.930	0.016	0.009	0.017
	9	3	0.950	0.023	0.013	0.024
	16	3	1.170	0.022	0.013	0.019
THSO	0.3	3	0.560	0.022	0.013	0.039
	0.6	3	0.860	0.018	0.010	0.021
	1.3	3	0.870	0.019	0.011	0.022
	2.5	3	0.890	0.029	0.017	0.033
	5	3	0.920	0.021	0.012	0.023
	9	3	1.000	0.011	0.006	0.011
	16	3	1.020	0.020	0.012	0.020

Post Hoc Tests

Standard (HSD)

Post Hoc Comparisons - sample ✲ concentration

		Mean Difference	95% CI for Mean Difference		SE	df	t	p_{tukey}
			Lower	Upper				
AHSO 0.3	SHSO 0.3	0.250	0.186	0.314	0.017	42	14.891	< .001***
	THSO 0.3	0.180	0.116	0.244	0.017	42	10.721	< .001***
	AHSO 0.6	-0.110	-0.174	-0.046	0.017	42	-6.552	< .001***
	SHSO 0.6	-0.090	-0.154	-0.026	0.017	42	-5.361	< .001***
	THSO 0.6	-0.120	-0.184	-0.056	0.017	42	-7.148	< .001***
	AHSO 1.3	-0.120	-0.184	-0.056	0.017	42	-7.148	< .001***
	SHSO 1.3	-0.120	-0.184	-0.056	0.017	42	-7.148	< .001***
	THSO 1.3	-0.130	-0.194	-0.066	0.017	42	-7.743	< .001***
	AHSO 2.5	-0.140	-0.204	-0.076	0.017	42	-8.339	< .001***
	SHSO 2.5	-0.130	-0.194	-0.066	0.017	42	-7.743	< .001***
	THSO 2.5	-0.150	-0.214	-0.086	0.017	42	-8.934	< .001***
	AHSO 5	-0.140	-0.204	-0.076	0.017	42	-8.339	< .001***
	SHSO 5	-0.190	-0.254	-0.126	0.017	42	-11.317	< .001***
	THSO 5	-0.180	-0.244	-0.116	0.017	42	-10.721	< .001***
	AHSO 9	-0.150	-0.214	-0.086	0.017	42	-8.934	< .001***
	SHSO 9	-0.210	-0.274	-0.146	0.017	42	-12.508	< .001***
	THSO 9	-0.260	-0.324	-0.196	0.017	42	-15.486	< .001***
	AHSO 16	-0.190	-0.254	-0.126	0.017	42	-11.317	< .001***
	SHSO 16	-0.430	-0.494	-0.366	0.017	42	-25.612	< .001***
	THSO 16	-0.280	-0.344	-0.216	0.017	42	-16.678	< .001***
SHSO 0.3	THSO 0.3	-0.070	-0.134	-0.006	0.017	42	-4.169	0.019*
	AHSO 0.6	-0.360	-0.424	-0.296	0.017	42	-21.443	< .001***
	SHSO 0.6	-0.340	-0.404	-0.276	0.017	42	-20.251	< .001***
	THSO 0.6	-0.370	-0.434	-0.306	0.017	42	-22.038	< .001***

Post Hoc Comparisons - sample ✲ concentration

		Mean Difference	95% CI for Mean Difference		SE	df	t	p_{tukey}
			Lower	Upper				
	AHSO 1.3	-0.370	-0.434	-0.306	0.017	42	-22.038	< .001***
	SHSO 1.3	-0.370	-0.434	-0.306	0.017	42	-22.038	< .001***
	THSO 1.3	-0.380	-0.444	-0.316	0.017	42	-22.634	< .001***
	AHSO 2.5	-0.390	-0.454	-0.326	0.017	42	-23.229	< .001***
	SHSO 2.5	-0.380	-0.444	-0.316	0.017	42	-22.634	< .001***
	THSO 2.5	-0.400	-0.464	-0.336	0.017	42	-23.825	< .001***
	AHSO 5	-0.390	-0.454	-0.326	0.017	42	-23.229	< .001***
	SHSO 5	-0.440	-0.504	-0.376	0.017	42	-26.208	< .001***
	THSO 5	-0.430	-0.494	-0.366	0.017	42	-25.612	< .001***
	AHSO 9	-0.400	-0.464	-0.336	0.017	42	-23.825	< .001***
	SHSO 9	-0.460	-0.524	-0.396	0.017	42	-27.399	< .001***
	THSO 9	-0.510	-0.574	-0.446	0.017	42	-30.377	< .001***
	AHSO 16	-0.440	-0.504	-0.376	0.017	42	-26.208	< .001***
	SHSO 16	-0.680	-0.744	-0.616	0.017	42	-40.503	< .001***
	THSO 16	-0.530	-0.594	-0.466	0.017	42	-31.568	< .001***
THSO 0.3	AHSO 0.6	-0.290	-0.354	-0.226	0.017	42	-17.273	< .001***
	SHSO 0.6	-0.270	-0.334	-0.206	0.017	42	-16.082	< .001***
	THSO 0.6	-0.300	-0.364	-0.236	0.017	42	-17.869	< .001***
	AHSO 1.3	-0.300	-0.364	-0.236	0.017	42	-17.869	< .001***
	SHSO 1.3	-0.300	-0.364	-0.236	0.017	42	-17.869	< .001***
	THSO 1.3	-0.310	-0.374	-0.246	0.017	42	-18.464	< .001***
	AHSO 2.5	-0.320	-0.384	-0.256	0.017	42	-19.060	< .001***
	SHSO 2.5	-0.310	-0.374	-0.246	0.017	42	-18.464	< .001***
	THSO 2.5	-0.330	-0.394	-0.266	0.017	42	-19.656	< .001***
	AHSO 5	-0.320	-0.384	-0.256	0.017	42	-19.060	< .001***

Post Hoc Comparisons - sample ✻ concentration

		Mean Difference	95% CI for Mean Difference		SE	df	t	p_{tukey}
			Lower	Upper				
	SHSO 5	-0.370	-0.434	-0.306	0.017	42	-22.038	< .001***
	THSO 5	-0.360	-0.424	-0.296	0.017	42	-21.443	< .001***
	AHSO 9	-0.330	-0.394	-0.266	0.017	42	-19.656	< .001***
	SHSO 9	-0.390	-0.454	-0.326	0.017	42	-23.229	< .001***
	THSO 9	-0.440	-0.504	-0.376	0.017	42	-26.208	< .001***
	AHSO 16	-0.370	-0.434	-0.306	0.017	42	-22.038	< .001***
	SHSO 16	-0.610	-0.674	-0.546	0.017	42	-36.333	< .001***
	THSO 16	-0.460	-0.524	-0.396	0.017	42	-27.399	< .001***
AHSO 0.6	SHSO 0.6	0.020	-0.044	0.084	0.017	42	1.191	1.000
	THSO 0.6	-0.010	-0.074	0.054	0.017	42	-0.596	1.000
	AHSO 1.3	-0.010	-0.074	0.054	0.017	42	-0.596	1.000
	SHSO 1.3	-0.010	-0.074	0.054	0.017	42	-0.596	1.000
	THSO 1.3	-0.020	-0.084	0.044	0.017	42	-1.191	1.000
	AHSO 2.5	-0.030	-0.094	0.034	0.017	42	-1.787	0.960
	SHSO 2.5	-0.020	-0.084	0.044	0.017	42	-1.191	1.000
	THSO 2.5	-0.040	-0.104	0.024	0.017	42	-2.383	0.691
	AHSO 5	-0.030	-0.094	0.034	0.017	42	-1.787	0.960
	SHSO 5	-0.080	-0.144	-0.016	0.017	42	-4.765	0.003**
	THSO 5	-0.070	-0.134	-0.006	0.017	42	-4.169	0.019*
	AHSO 9	-0.040	-0.104	0.024	0.017	42	-2.383	0.691
	SHSO 9	-0.100	-0.164	-0.036	0.017	42	-5.956	< .001***
	THSO 9	-0.150	-0.214	-0.086	0.017	42	-8.934	< .001***
	AHSO 16	-0.080	-0.144	-0.016	0.017	42	-4.765	0.003**
	SHSO 16	-0.320	-0.384	-0.256	0.017	42	-19.060	< .001***
	THSO 16	-0.170	-0.234	-0.106	0.017	42	-10.126	< .001***
SHSO 0.6	THSO 0.6	-0.030	-0.094	0.034	0.017	42	-1.787	0.960
	AHSO 1.3	-0.030	-0.094	0.034	0.017	42	-1.787	0.960

Post Hoc Comparisons - sample ✻ concentration

			95% CI for Mean Difference					
		Mean Difference	Lower	Upper	SE	df	t	p_{tukey}
	SHSO 1.3	-0.030	-0.094	0.034	0.017	42	-1.787	0.960
	THSO 1.3	-0.040	-0.104	0.024	0.017	42	-2.383	0.691
	AHSO 2.5	-0.050	-0.114	0.014	0.017	42	-2.978	0.305
	SHSO 2.5	-0.040	-0.104	0.024	0.017	42	-2.383	0.691
	THSO 2.5	-0.060	-0.124	0.004	0.017	42	-3.574	0.088
	AHSO 5	-0.050	-0.114	0.014	0.017	42	-2.978	0.305
	SHSO 5	-0.100	-0.164	-0.036	0.017	42	-5.956	< .001***
	THSO 5	-0.090	-0.154	-0.026	0.017	42	-5.361	< .001***
	AHSO 9	-0.060	-0.124	0.004	0.017	42	-3.574	0.088
	SHSO 9	-0.120	-0.184	-0.056	0.017	42	-7.148	< .001***
	THSO 9	-0.170	-0.234	-0.106	0.017	42	-10.126	< .001***
	AHSO 16	-0.100	-0.164	-0.036	0.017	42	-5.956	< .001***
	SHSO 16	-0.340	-0.404	-0.276	0.017	42	-20.251	< .001***
	THSO 16	-0.190	-0.254	-0.126	0.017	42	-11.317	< .001***
THSO 0.6	AHSO 1.3	7.936×10^{-17}	-0.064	0.064	0.017	42	4.727×10^{-15}	1.000
	SHSO 1.3	-9.714×10^{-17}	-0.064	0.064	0.017	42	-5.786×10^{-15}	1.000
	THSO 1.3	-0.010	-0.074	0.054	0.017	42	-0.596	1.000
	AHSO 2.5	-0.020	-0.084	0.044	0.017	42	-1.191	1.000
	SHSO 2.5	-0.010	-0.074	0.054	0.017	42	-0.596	1.000
	THSO 2.5	-0.030	-0.094	0.034	0.017	42	-1.787	0.960
	AHSO 5	-0.020	-0.084	0.044	0.017	42	-1.191	1.000
	SHSO 5	-0.070	-0.134	-0.006	0.017	42	-4.169	0.019*
	THSO 5	-0.060	-0.124	0.004	0.017	42	-3.574	0.088
	AHSO 9	-0.030	-0.094	0.034	0.017	42	-1.787	0.960
	SHSO 9	-0.090	-0.154	-0.026	0.017	42	-5.361	< .001***
	THSO 9	-0.140	-0.204	-0.076	0.017	42	-8.339	< .001***
	AHSO 16	-0.070	-0.134	-0.006	0.017	42	-4.169	0.019*

Post Hoc Comparisons - sample ✻ concentration

		Mean Difference	95% CI for Mean Difference		SE	df	t	p_{tukey}
			Lower	Upper				
	SHSO 16	-0.310	-0.374	-0.246	0.017	42	-18.464	< .001***
	THSO 16	-0.160	-0.224	-0.096	0.017	42	-9.530	< .001***
AHSO 1.3	SHSO 1.3	-1.765×10^{-16}	-0.064	0.064	0.017	42	-1.051×10^{-14}	1.000
	THSO 1.3	-0.010	-0.074	0.054	0.017	42	-0.596	1.000
	AHSO 2.5	-0.020	-0.084	0.044	0.017	42	-1.191	1.000
	SHSO 2.5	-0.010	-0.074	0.054	0.017	42	-0.596	1.000
	THSO 2.5	-0.030	-0.094	0.034	0.017	42	-1.787	0.960
	AHSO 5	-0.020	-0.084	0.044	0.017	42	-1.191	1.000
	SHSO 5	-0.070	-0.134	-0.006	0.017	42	-4.169	0.019*
	THSO 5	-0.060	-0.124	0.004	0.017	42	-3.574	0.088
	AHSO 9	-0.030	-0.094	0.034	0.017	42	-1.787	0.960
	SHSO 9	-0.090	-0.154	-0.026	0.017	42	-5.361	< .001***
	THSO 9	-0.140	-0.204	-0.076	0.017	42	-8.339	< .001***
	AHSO 16	-0.070	-0.134	-0.006	0.017	42	-4.169	0.019*
	SHSO 16	-0.310	-0.374	-0.246	0.017	42	-18.464	< .001***
	THSO 16	-0.160	-0.224	-0.096	0.017	42	-9.530	< .001***
SHSO 1.3	THSO 1.3	-0.010	-0.074	0.054	0.017	42	-0.596	1.000
	AHSO 2.5	-0.020	-0.084	0.044	0.017	42	-1.191	1.000
	SHSO 2.5	-0.010	-0.074	0.054	0.017	42	-0.596	1.000
	THSO 2.5	-0.030	-0.094	0.034	0.017	42	-1.787	0.960
	AHSO 5	-0.020	-0.084	0.044	0.017	42	-1.191	1.000
	SHSO 5	-0.070	-0.134	-0.006	0.017	42	-4.169	0.019*
	THSO 5	-0.060	-0.124	0.004	0.017	42	-3.574	0.088
	AHSO 9	-0.030	-0.094	0.034	0.017	42	-1.787	0.960
	SHSO 9	-0.090	-0.154	-0.026	0.017	42	-5.361	< .001***
	THSO 9	-0.140	-0.204	-0.076	0.017	42	-8.339	< .001***
	AHSO 16	-0.070	-0.134	-0.006	0.017	42	-4.169	0.019*

Post Hoc Comparisons - sample ✲ concentration

		Mean Difference	95% CI for Mean Difference		SE	df	t	p_{tukey}
			Lower	Upper				
	SHSO 16	-0.310	-0.374	-0.246	0.017	42	-18.464	< .001***
	THSO 16	-0.160	-0.224	-0.096	0.017	42	-9.530	< .001***
THSO 1.3	AHSO 2.5	-0.010	-0.074	0.054	0.017	42	-0.596	1.000
	SHSO 2.5	-2.533×10^{-16}	-0.064	0.064	0.017	42	-1.509×10^{-14}	1.000
	THSO 2.5	-0.020	-0.084	0.044	0.017	42	-1.191	1.000
	AHSO 5	-0.010	-0.074	0.054	0.017	42	-0.596	1.000
	SHSO 5	-0.060	-0.124	0.004	0.017	42	-3.574	0.088
	THSO 5	-0.050	-0.114	0.014	0.017	42	-2.978	0.305
	AHSO 9	-0.020	-0.084	0.044	0.017	42	-1.191	1.000
	SHSO 9	-0.080	-0.144	-0.016	0.017	42	-4.765	0.003**
	THSO 9	-0.130	-0.194	-0.066	0.017	42	-7.743	< .001***
	AHSO 16	-0.060	-0.124	0.004	0.017	42	-3.574	0.088
	SHSO 16	-0.300	-0.364	-0.236	0.017	42	-17.869	< .001***
	THSO 16	-0.150	-0.214	-0.086	0.017	42	-8.934	< .001***
AHSO 2.5	SHSO 2.5	0.010	-0.054	0.074	0.017	42	0.596	1.000
	THSO 2.5	-0.010	-0.074	0.054	0.017	42	-0.596	1.000
	AHSO 5	3.643×10^{-17}	-0.064	0.064	0.017	42	2.170×10^{-15}	1.000
	SHSO 5	-0.050	-0.114	0.014	0.017	42	-2.978	0.305
	THSO 5	-0.040	-0.104	0.024	0.017	42	-2.383	0.691
	AHSO 9	-0.010	-0.074	0.054	0.017	42	-0.596	1.000
	SHSO 9	-0.070	-0.134	-0.006	0.017	42	-4.169	0.019*
	THSO 9	-0.120	-0.184	-0.056	0.017	42	-7.148	< .001***
	AHSO 16	-0.050	-0.114	0.014	0.017	42	-2.978	0.305
	SHSO 16	-0.290	-0.354	-0.226	0.017	42	-17.273	< .001***
	THSO 16	-0.140	-0.204	-0.076	0.017	42	-8.339	< .001***
SHSO 2.5	THSO 2.5	-0.020	-0.084	0.044	0.017	42	-1.191	1.000
	AHSO 5	-0.010	-0.074	0.054	0.017	42	-0.596	1.000
	SHSO 5	-0.060	-0.124	0.004	0.017	42	-3.574	0.088
	THSO 5	-0.050	-0.114	0.014	0.017	42	-2.978	0.305

Post Hoc Comparisons - sample ✲ concentration

		Mean Difference	95% CI for Mean Difference Lower	95% CI for Mean Difference Upper	SE	df	t	p_{tukey}
	AHSO 9	-0.020	-0.084	0.044	0.017	42	-1.191	1.000
	SHSO 9	-0.080	-0.144	-0.016	0.017	42	-4.765	0.003**
	THSO 9	-0.130	-0.194	-0.066	0.017	42	-7.743	< .001***
	AHSO 16	-0.060	-0.124	0.004	0.017	42	-3.574	0.088
	SHSO 16	-0.300	-0.364	-0.236	0.017	42	-17.869	< .001***
	THSO 16	-0.150	-0.214	-0.086	0.017	42	-8.934	< .001***
THSO 2.5	AHSO 5	0.010	-0.054	0.074	0.017	42	0.596	1.000
	SHSO 5	-0.040	-0.104	0.024	0.017	42	-2.383	0.691
	THSO 5	-0.030	-0.094	0.034	0.017	42	-1.787	0.960
	AHSO 9	-6.375×10^{-17}	-0.064	0.064	0.017	42	-3.797×10^{-15}	1.000
	SHSO 9	-0.060	-0.124	0.004	0.017	42	-3.574	0.088
	THSO 9	-0.110	-0.174	-0.046	0.017	42	-6.552	< .001***
	AHSO 16	-0.040	-0.104	0.024	0.017	42	-2.383	0.691
	SHSO 16	-0.280	-0.344	-0.216	0.017	42	-16.678	< .001***
	THSO 16	-0.130	-0.194	-0.066	0.017	42	-7.743	< .001***
AHSO 5	SHSO 5	-0.050	-0.114	0.014	0.017	42	-2.978	0.305
	THSO 5	-0.040	-0.104	0.024	0.017	42	-2.383	0.691
	AHSO 9	-0.010	-0.074	0.054	0.017	42	-0.596	1.000
	SHSO 9	-0.070	-0.134	-0.006	0.017	42	-4.169	0.019*
	THSO 9	-0.120	-0.184	-0.056	0.017	42	-7.148	< .001***
	AHSO 16	-0.050	-0.114	0.014	0.017	42	-2.978	0.305
	SHSO 16	-0.290	-0.354	-0.226	0.017	42	-17.273	< .001***
	THSO 16	-0.140	-0.204	-0.076	0.017	42	-8.339	< .001***
SHSO 5	THSO 5	0.010	-0.054	0.074	0.017	42	0.596	1.000
	AHSO 9	0.040	-0.024	0.104	0.017	42	2.383	0.691
	SHSO 9	-0.020	-0.084	0.044	0.017	42	-1.191	1.000
	THSO 9	-0.070	-0.134	-0.006	0.017	42	-4.169	0.019*
	AHSO 16	1.904×10^{-16}	-0.064	0.064	0.017	42	1.134×10^{-14}	1.000
	SHSO 16	-0.240	-0.304	-0.176	0.017	42	-14.295	< .001***

Post Hoc Comparisons - sample ✲ concentration

		Mean Difference	95% CI for Mean Difference		SE	df	t	p_{tukey}
			Lower	Upper				
	THSO 16	-0.090	-0.154	-0.026	0.017	42	-5.361	< .001***
THSO 5	AHSO 9	0.030	-0.034	0.094	0.017	42	1.787	0.960
	SHSO 9	-0.030	-0.094	0.034	0.017	42	-1.787	0.960
	THSO 9	-0.080	-0.144	-0.016	0.017	42	-4.765	0.003**
	AHSO 16	-0.010	-0.074	0.054	0.017	42	-0.596	1.000
	SHSO 16	-0.250	-0.314	-0.186	0.017	42	-14.891	< .001***
	THSO 16	-0.100	-0.164	-0.036	0.017	42	-5.956	< .001***
AHSO 9	SHSO 9	-0.060	-0.124	0.004	0.017	42	-3.574	0.088
	THSO 9	-0.110	-0.174	-0.046	0.017	42	-6.552	< .001***
	AHSO 16	-0.040	-0.104	0.024	0.017	42	-2.383	0.691
	SHSO 16	-0.280	-0.344	-0.216	0.017	42	-16.678	< .001***
	THSO 16	-0.130	-0.194	-0.066	0.017	42	-7.743	< .001***
SHSO 9	THSO 9	-0.050	-0.114	0.014	0.017	42	-2.978	0.305
	AHSO 16	0.020	-0.044	0.084	0.017	42	1.191	1.000
	SHSO 16	-0.220	-0.284	-0.156	0.017	42	-13.104	< .001***
	THSO 16	-0.070	-0.134	-0.006	0.017	42	-4.169	0.019*
THSO 9	AHSO 16	0.070	0.006	0.134	0.017	42	4.169	0.019*
	SHSO 16	-0.170	-0.234	-0.106	0.017	42	-10.126	< .001***
	THSO 16	-0.020	-0.084	0.044	0.017	42	-1.191	1.000
AHSO 16	SHSO 16	-0.240	-0.304	-0.176	0.017	42	-14.295	< .001***
	THSO 16	-0.090	-0.154	-0.026	0.017	42	-5.361	< .001***
SHSO 16	THSO 16	0.150	0.086	0.214	0.017	42	8.934	< .001***

* p < .05, ** p < .01, *** p < .001

Note. P-value and confidence intervals adjusted for comparing a family of 21 estimates (confidence intervals corrected using the tukey method).

Letter-Based Grouping - sample ✻ concentration

sample	concentration	Letter
AHSO	0.3	c
SHSO		a
THSO		b
AHSO	0.6	d
SHSO		d
THSO		de
AHSO	1.3	de
SHSO		de
THSO		def
AHSO	2.5	def
SHSO		def
THSO		defg
AHSO	5	def
SHSO		fg
THSO		efg
AHSO	9	defg
SHSO		gh
THSO		hi
AHSO	16	fg
SHSO		j
THSO		i

Note. If two or more means share the same grouping symbol, then we cannot show them to be different, but we also did not show them to be the same.

ANOVA

ANOVA - Lactobacillus paracasei subsp.paracasei ATCC BAA-52

Cases	Sum of Squares	df	Mean Square	F	p
sample	0.008	2	0.004	8.964	< .001
concentration	0.736	6	0.123	283.969	< .001
sample ✻ concentration	0.269	12	0.022	51.990	< .001
Residuals	0.018	42	4.319×10^{-4}		

Note. Type III Sum of Squares

Descriptives

Descriptives - Lactobacillus paracasei subsp.paracasei ATCC BAA-52

sample	concentration	N	Mean	SD	SE	Coefficient of variation
AHSO	0.3	3	0.690	0.012	0.007	0.017
	0.6	3	0.890	0.014	0.008	0.016
	1.3	3	0.900	0.017	0.010	0.019
	2.5	3	0.910	0.024	0.014	0.026
	5	3	0.910	0.015	0.009	0.016
	9	3	0.910	0.029	0.017	0.032
	16	3	0.920	0.024	0.014	0.026
SHSO	0.3	3	0.760	0.023	0.013	0.030
	0.6	3	0.880	0.019	0.011	0.022
	1.3	3	0.890	0.021	0.012	0.024
	2.5	3	0.910	0.013	0.008	0.014
	5	3	0.910	0.022	0.013	0.024
	9	3	0.920	0.020	0.012	0.022
	16	3	0.960	0.018	0.010	0.019
THSO	0.3	3	0.500	0.016	0.009	0.032
	0.6	3	0.860	0.028	0.016	0.033
	1.3	3	0.890	0.025	0.014	0.028
	2.5	3	0.900	0.027	0.016	0.030
	5	3	0.970	0.018	0.010	0.019
	9	3	0.990	0.026	0.015	0.026
	16	3	1.210	0.011	0.006	0.009

Post Hoc Tests

Standard (HSD)

Post Hoc Comparisons - sample ✻ concentration

			95% CI for Mean Difference					
		Mean Difference	Lower	Upper	SE	df	t	p_{tukey}
AHSO 0.3	SHSO 0.3	-0.070	-0.135	-0.005	0.017	42	-4.125	0.022*
	THSO 0.3	0.190	0.125	0.255	0.017	42	11.197	< .001***
	AHSO 0.6	-0.200	-0.265	-0.135	0.017	42	-11.786	< .001***
	SHSO 0.6	-0.190	-0.255	-0.125	0.017	42	-11.197	< .001***
	THSO 0.6	-0.170	-0.235	-0.105	0.017	42	-10.018	< .001***
	AHSO 1.3	-0.210	-0.275	-0.145	0.017	42	-12.376	< .001***

Post Hoc Comparisons - sample ✲ concentration

		Mean Difference	95% CI for Mean Difference		SE	df	t	p_{tukey}
			Lower	Upper				
	SHSO 1.3	-0.200	-0.265	-0.135	0.017	42	-11.786	< .001***
	THSO 1.3	-0.200	-0.265	-0.135	0.017	42	-11.786	< .001***
	AHSO 2.5	-0.220	-0.285	-0.155	0.017	42	-12.965	< .001***
	SHSO 2.5	-0.220	-0.285	-0.155	0.017	42	-12.965	< .001***
	THSO 2.5	-0.210	-0.275	-0.145	0.017	42	-12.376	< .001***
	AHSO 5	-0.220	-0.285	-0.155	0.017	42	-12.965	< .001***
	SHSO 5	-0.220	-0.285	-0.155	0.017	42	-12.965	< .001***
	THSO 5	-0.280	-0.345	-0.215	0.017	42	-16.501	< .001***
	AHSO 9	-0.220	-0.285	-0.155	0.017	42	-12.965	< .001***
	SHSO 9	-0.230	-0.295	-0.165	0.017	42	-13.554	< .001***
	THSO 9	-0.300	-0.365	-0.235	0.017	42	-17.680	< .001***
	AHSO 16	-0.230	-0.295	-0.165	0.017	42	-13.554	< .001***
	SHSO 16	-0.270	-0.335	-0.205	0.017	42	-15.912	< .001***
	THSO 16	-0.520	-0.585	-0.455	0.017	42	-30.645	< .001***
SHSO 0.3	THSO 0.3	0.260	0.195	0.325	0.017	42	15.322	< .001***
	AHSO 0.6	-0.130	-0.195	-0.065	0.017	42	-7.661	< .001***
	SHSO 0.6	-0.120	-0.185	-0.055	0.017	42	-7.072	< .001***
	THSO 0.6	-0.100	-0.165	-0.035	0.017	42	-5.893	< .001***
	AHSO 1.3	-0.140	-0.205	-0.075	0.017	42	-8.250	< .001***
	SHSO 1.3	-0.130	-0.195	-0.065	0.017	42	-7.661	< .001***
	THSO 1.3	-0.130	-0.195	-0.065	0.017	42	-7.661	< .001***
	AHSO 2.5	-0.150	-0.215	-0.085	0.017	42	-8.840	< .001***
	SHSO 2.5	-0.150	-0.215	-0.085	0.017	42	-8.840	< .001***
	THSO 2.5	-0.140	-0.205	-0.075	0.017	42	-8.250	< .001***
	AHSO 5	-0.150	-0.215	-0.085	0.017	42	-8.840	< .001***

Post Hoc Comparisons - sample ✻ concentration

		Mean Difference	95% CI for Mean Difference		SE	df	t	p_{tukey}
			Lower	Upper				
	SHSO 5	-0.150	-0.215	-0.085	0.017	42	-8.840	< .001***
	THSO 5	-0.210	-0.275	-0.145	0.017	42	-12.376	< .001***
	AHSO 9	-0.150	-0.215	-0.085	0.017	42	-8.840	< .001***
	SHSO 9	-0.160	-0.225	-0.095	0.017	42	-9.429	< .001***
	THSO 9	-0.230	-0.295	-0.165	0.017	42	-13.554	< .001***
	AHSO 16	-0.160	-0.225	-0.095	0.017	42	-9.429	< .001***
	SHSO 16	-0.200	-0.265	-0.135	0.017	42	-11.786	< .001***
	THSO 16	-0.450	-0.515	-0.385	0.017	42	-26.519	< .001***
THSO 0.3	AHSO 0.6	-0.390	-0.455	-0.325	0.017	42	-22.984	< .001***
	SHSO 0.6	-0.380	-0.445	-0.315	0.017	42	-22.394	< .001***
	THSO 0.6	-0.360	-0.425	-0.295	0.017	42	-21.216	< .001***
	AHSO 1.3	-0.400	-0.465	-0.335	0.017	42	-23.573	< .001***
	SHSO 1.3	-0.390	-0.455	-0.325	0.017	42	-22.984	< .001***
	THSO 1.3	-0.390	-0.455	-0.325	0.017	42	-22.984	< .001***
	AHSO 2.5	-0.410	-0.475	-0.345	0.017	42	-24.162	< .001***
	SHSO 2.5	-0.410	-0.475	-0.345	0.017	42	-24.162	< .001***
	THSO 2.5	-0.400	-0.465	-0.335	0.017	42	-23.573	< .001***
	AHSO 5	-0.410	-0.475	-0.345	0.017	42	-24.162	< .001***
	SHSO 5	-0.410	-0.475	-0.345	0.017	42	-24.162	< .001***
	THSO 5	-0.470	-0.535	-0.405	0.017	42	-27.698	< .001***
	AHSO 9	-0.410	-0.475	-0.345	0.017	42	-24.162	< .001***
	SHSO 9	-0.420	-0.485	-0.355	0.017	42	-24.751	< .001***
	THSO 9	-0.490	-0.555	-0.425	0.017	42	-28.877	< .001***
	AHSO 16	-0.420	-0.485	-0.355	0.017	42	-24.751	< .001***
	SHSO 16	-0.460	-0.525	-0.395	0.017	42	-27.109	< .001***
	THSO 16	-0.710	-0.775	-0.645	0.017	42	-41.842	< .001***
AHSO 0.6	SHSO 0.6	0.010	-0.055	0.075	0.017	42	0.589	1.000

Post Hoc Comparisons - sample ✻ concentration

		Mean Difference	95% CI for Mean Difference		SE	df	t	p_{tukey}
			Lower	Upper				
	THSO 0.6	0.030	-0.035	0.095	0.017	42	1.768	0.964
	AHSO 1.3	-0.010	-0.075	0.055	0.017	42	-0.589	1.000
	SHSO 1.3	-1.689×10^{-16}	-0.065	0.065	0.017	42	-9.955×10^{-15}	1.000
	THSO 1.3	3.014×10^{-17}	-0.065	0.065	0.017	42	1.776×10^{-15}	1.000
	AHSO 2.5	-0.020	-0.085	0.045	0.017	42	-1.179	1.000
	SHSO 2.5	-0.020	-0.085	0.045	0.017	42	-1.179	1.000
	THSO 2.5	-0.010	-0.075	0.055	0.017	42	-0.589	1.000
	AHSO 5	-0.020	-0.085	0.045	0.017	42	-1.179	1.000
	SHSO 5	-0.020	-0.085	0.045	0.017	42	-1.179	1.000
	THSO 5	-0.080	-0.145	-0.015	0.017	42	-4.715	0.004**
	AHSO 9	-0.020	-0.085	0.045	0.017	42	-1.179	1.000
	SHSO 9	-0.030	-0.095	0.035	0.017	42	-1.768	0.964
	THSO 9	-0.100	-0.165	-0.035	0.017	42	-5.893	< .001***
	AHSO 16	-0.030	-0.095	0.035	0.017	42	-1.768	0.964
	SHSO 16	-0.070	-0.135	-0.005	0.017	42	-4.125	0.022*
	THSO 16	-0.320	-0.385	-0.255	0.017	42	-18.858	< .001***
SHSO 0.6	THSO 0.6	0.020	-0.045	0.085	0.017	42	1.179	1.000
	AHSO 1.3	-0.020	-0.085	0.045	0.017	42	-1.179	1.000
	SHSO 1.3	-0.010	-0.075	0.055	0.017	42	-0.589	1.000
	THSO 1.3	-0.010	-0.075	0.055	0.017	42	-0.589	1.000
	AHSO 2.5	-0.030	-0.095	0.035	0.017	42	-1.768	0.964
	SHSO 2.5	-0.030	-0.095	0.035	0.017	42	-1.768	0.964
	THSO 2.5	-0.020	-0.085	0.045	0.017	42	-1.179	1.000
	AHSO 5	-0.030	-0.095	0.035	0.017	42	-1.768	0.964
	SHSO 5	-0.030	-0.095	0.035	0.017	42	-1.768	0.964
	THSO 5	-0.090	-0.155	-0.025	0.017	42	-5.304	< .001***

Post Hoc Comparisons - sample ✻ concentration

			95% CI for Mean Difference					
		Mean Difference	Lower	Upper	SE	df	t	p_{tukey}
	AHSO 9	-0.030	-0.095	0.035	0.017	42	-1.768	0.964
	SHSO 9	-0.040	-0.105	0.025	0.017	42	-2.357	0.708
	THSO 9	-0.110	-0.175	-0.045	0.017	42	-6.483	< .001***
	AHSO 16	-0.040	-0.105	0.025	0.017	42	-2.357	0.708
	SHSO 16	-0.080	-0.145	-0.015	0.017	42	-4.715	0.004**
	THSO 16	-0.330	-0.395	-0.265	0.017	42	-19.448	< .001***
THSO 0.6	AHSO 1.3	-0.040	-0.105	0.025	0.017	42	-2.357	0.708
	SHSO 1.3	-0.030	-0.095	0.035	0.017	42	-1.768	0.964
	THSO 1.3	-0.030	-0.095	0.035	0.017	42	-1.768	0.964
	AHSO 2.5	-0.050	-0.115	0.015	0.017	42	-2.947	0.323
	SHSO 2.5	-0.050	-0.115	0.015	0.017	42	-2.947	0.323
	THSO 2.5	-0.040	-0.105	0.025	0.017	42	-2.357	0.708
	AHSO 5	-0.050	-0.115	0.015	0.017	42	-2.947	0.323
	SHSO 5	-0.050	-0.115	0.015	0.017	42	-2.947	0.323
	THSO 5	-0.110	-0.175	-0.045	0.017	42	-6.483	< .001***
	AHSO 9	-0.050	-0.115	0.015	0.017	42	-2.947	0.323
	SHSO 9	-0.060	-0.125	0.005	0.017	42	-3.536	0.097
	THSO 9	-0.130	-0.195	-0.065	0.017	42	-7.661	< .001***
	AHSO 16	-0.060	-0.125	0.005	0.017	42	-3.536	0.097
	SHSO 16	-0.100	-0.165	-0.035	0.017	42	-5.893	< .001***
	THSO 16	-0.350	-0.415	-0.285	0.017	42	-20.626	< .001***
AHSO 1.3	SHSO 1.3	0.010	-0.055	0.075	0.017	42	0.589	1.000
	THSO 1.3	0.010	-0.055	0.075	0.017	42	0.589	1.000
	AHSO 2.5	-0.010	-0.075	0.055	0.017	42	-0.589	1.000
	SHSO 2.5	-0.010	-0.075	0.055	0.017	42	-0.589	1.000
	THSO 2.5	9.216×10^{-17}	-0.065	0.065	0.017	42	5.431×10^{-15}	1.000

Post Hoc Comparisons - sample ✻ concentration

		Mean Difference	95% CI for Mean Difference		SE	df	t	p_{tukey}
			Lower	Upper				
	AHSO 5	-0.010	-0.075	0.055	0.017	42	-0.589	1.000
	SHSO 5	-0.010	-0.075	0.055	0.017	42	-0.589	1.000
	THSO 5	-0.070	-0.135	-0.005	0.017	42	-4.125	0.022*
	AHSO 9	-0.010	-0.075	0.055	0.017	42	-0.589	1.000
	SHSO 9	-0.020	-0.085	0.045	0.017	42	-1.179	1.000
	THSO 9	-0.090	-0.155	-0.025	0.017	42	-5.304	< .001***
	AHSO 16	-0.020	-0.085	0.045	0.017	42	-1.179	1.000
	SHSO 16	-0.060	-0.125	0.005	0.017	42	-3.536	0.097
	THSO 16	-0.310	-0.375	-0.245	0.017	42	-18.269	< .001***
SHSO 1.3	THSO 1.3	1.991×10^{-16}	-0.065	0.065	0.017	42	1.173×10^{-14}	1.000
	AHSO 2.5	-0.020	-0.085	0.045	0.017	42	-1.179	1.000
	SHSO 2.5	-0.020	-0.085	0.045	0.017	42	-1.179	1.000
	THSO 2.5	-0.010	-0.075	0.055	0.017	42	-0.589	1.000
	AHSO 5	-0.020	-0.085	0.045	0.017	42	-1.179	1.000
	SHSO 5	-0.020	-0.085	0.045	0.017	42	-1.179	1.000
	THSO 5	-0.080	-0.145	-0.015	0.017	42	-4.715	0.004**
	AHSO 9	-0.020	-0.085	0.045	0.017	42	-1.179	1.000
	SHSO 9	-0.030	-0.095	0.035	0.017	42	-1.768	0.964
	THSO 9	-0.100	-0.165	-0.035	0.017	42	-5.893	< .001***
	AHSO 16	-0.030	-0.095	0.035	0.017	42	-1.768	0.964
	SHSO 16	-0.070	-0.135	-0.005	0.017	42	-4.125	0.022*
	THSO 16	-0.320	-0.385	-0.255	0.017	42	-18.858	< .001***
THSO 1.3	AHSO 2.5	-0.020	-0.085	0.045	0.017	42	-1.179	1.000
	SHSO 2.5	-0.020	-0.085	0.045	0.017	42	-1.179	1.000
	THSO 2.5	-0.010	-0.075	0.055	0.017	42	-0.589	1.000
	AHSO 5	-0.020	-0.085	0.045	0.017	42	-1.179	1.000
	SHSO 5	-0.020	-0.085	0.045	0.017	42	-1.179	1.000
	THSO 5	-0.080	-0.145	-0.015	0.017	42	-4.715	0.004**
	AHSO 9	-0.020	-0.085	0.045	0.017	42	-1.179	1.000
	SHSO 9	-0.030	-0.095	0.035	0.017	42	-1.768	0.964

Post Hoc Comparisons - sample ✻ concentration

		Mean Difference	95% CI for Mean Difference		SE	df	t	p_{tukey}
			Lower	Upper				
	THSO 9	-0.100	-0.165	-0.035	0.017	42	-5.893	< .001***
	AHSO 16	-0.030	-0.095	0.035	0.017	42	-1.768	0.964
	SHSO 16	-0.070	-0.135	-0.005	0.017	42	-4.125	0.022*
	THSO 16	-0.320	-0.385	-0.255	0.017	42	-18.858	< .001***
AHSO 2.5	SHSO 2.5	-1.685×10^{-16}	-0.065	0.065	0.017	42	-9.929×10^{-15}	1.000
	THSO 2.5	0.010	-0.055	0.075	0.017	42	0.589	1.000
	AHSO 5	-3.643×10^{-17}	-0.065	0.065	0.017	42	-2.147×10^{-15}	1.000
	SHSO 5	-2.032×10^{-16}	-0.065	0.065	0.017	42	-1.197×10^{-14}	1.000
	THSO 5	-0.060	-0.125	0.005	0.017	42	-3.536	0.097
	AHSO 9	-1.735×10^{-17}	-0.065	0.065	0.017	42	-1.022×10^{-15}	1.000
	SHSO 9	-0.010	-0.075	0.055	0.017	42	-0.589	1.000
	THSO 9	-0.080	-0.145	-0.015	0.017	42	-4.715	0.004**
	AHSO 16	-0.010	-0.075	0.055	0.017	42	-0.589	1.000
	SHSO 16	-0.050	-0.115	0.015	0.017	42	-2.947	0.323
	THSO 16	-0.300	-0.365	-0.235	0.017	42	-17.680	< .001***
SHSO 2.5	THSO 2.5	0.010	-0.055	0.075	0.017	42	0.589	1.000
	AHSO 5	1.321×10^{-16}	-0.065	0.065	0.017	42	7.782×10^{-15}	1.000
	SHSO 5	-3.469×10^{-17}	-0.065	0.065	0.017	42	-2.045×10^{-15}	1.000
	THSO 5	-0.060	-0.125	0.005	0.017	42	-3.536	0.097
	AHSO 9	1.511×10^{-16}	-0.065	0.065	0.017	42	8.907×10^{-15}	1.000
	SHSO 9	-0.010	-0.075	0.055	0.017	42	-0.589	1.000
	THSO 9	-0.080	-0.145	-0.015	0.017	42	-4.715	0.004**
	AHSO 16	-0.010	-0.075	0.055	0.017	42	-0.589	1.000
	SHSO 16	-0.050	-0.115	0.015	0.017	42	-2.947	0.323
	THSO 16	-0.300	-0.365	-0.235	0.017	42	-17.680	< .001***
THSO 2.5	AHSO 5	-0.010	-0.075	0.055	0.017	42	-0.589	1.000

Post Hoc Comparisons - sample ✻ concentration

		Mean Difference	95% CI for Mean Difference		SE	df	t	p_{tukey}
			Lower	Upper				
	SHSO 5	-0.010	-0.075	0.055	0.017	42	-0.589	1.000
	THSO 5	-0.070	-0.135	-0.005	0.017	42	-4.125	0.022*
	AHSO 9	-0.010	-0.075	0.055	0.017	42	-0.589	1.000
	SHSO 9	-0.020	-0.085	0.045	0.017	42	-1.179	1.000
	THSO 9	-0.090	-0.155	-0.025	0.017	42	-5.304	< .001***
	AHSO 16	-0.020	-0.085	0.045	0.017	42	-1.179	1.000
	SHSO 16	-0.060	-0.125	0.005	0.017	42	-3.536	0.097
	THSO 16	-0.310	-0.375	-0.245	0.017	42	-18.269	< .001***
AHSO 5	SHSO 5	-1.668×10^{-16}	-0.065	0.065	0.017	42	-9.827×10^{-15}	1.000
	THSO 5	-0.060	-0.125	0.005	0.017	42	-3.536	0.097
	AHSO 9	1.908×10^{-17}	-0.065	0.065	0.017	42	1.125×10^{-15}	1.000
	SHSO 9	-0.010	-0.075	0.055	0.017	42	-0.589	1.000
	THSO 9	-0.080	-0.145	-0.015	0.017	42	-4.715	0.004**
	AHSO 16	-0.010	-0.075	0.055	0.017	42	-0.589	1.000
	SHSO 16	-0.050	-0.115	0.015	0.017	42	-2.947	0.323
	THSO 16	-0.300	-0.365	-0.235	0.017	42	-17.680	< .001***
SHSO 5	THSO 5	-0.060	-0.125	0.005	0.017	42	-3.536	0.097
	AHSO 9	1.858×10^{-16}	-0.065	0.065	0.017	42	1.095×10^{-14}	1.000
	SHSO 9	-0.010	-0.075	0.055	0.017	42	-0.589	1.000
	THSO 9	-0.080	-0.145	-0.015	0.017	42	-4.715	0.004**
	AHSO 16	-0.010	-0.075	0.055	0.017	42	-0.589	1.000
	SHSO 16	-0.050	-0.115	0.015	0.017	42	-2.947	0.323
	THSO 16	-0.300	-0.365	-0.235	0.017	42	-17.680	< .001***
THSO 5	AHSO 9	0.060	-0.005	0.125	0.017	42	3.536	0.097
	SHSO 9	0.050	-0.015	0.115	0.017	42	2.947	0.323
	THSO 9	-0.020	-0.085	0.045	0.017	42	-1.179	1.000
	AHSO 16	0.050	-0.015	0.115	0.017	42	2.947	0.323
	SHSO 16	0.010	-0.055	0.075	0.017	42	0.589	1.000
	THSO 16	-0.240	-0.305	-0.175	0.017	42	-14.144	< .001***
AHSO 9	SHSO 9	-0.010	-0.075	0.055	0.017	42	-0.589	1.000

Post Hoc Comparisons - sample ✻ concentration

		Mean Difference	95% CI for Mean Difference		SE	df	t	p_{tukey}
			Lower	Upper				
	THSO 9	-0.080	-0.145	-0.015	0.017	42	-4.715	0.004**
	AHSO 16	-0.010	-0.075	0.055	0.017	42	-0.589	1.000
	SHSO 16	-0.050	-0.115	0.015	0.017	42	-2.947	0.323
	THSO 16	-0.300	-0.365	-0.235	0.017	42	-17.680	< .001***
SHSO 9	THSO 9	-0.070	-0.135	-0.005	0.017	42	-4.125	0.022*
	AHSO 16	5.052×10^{-17}	-0.065	0.065	0.017	42	2.977×10^{-15}	1.000
	SHSO 16	-0.040	-0.105	0.025	0.017	42	-2.357	0.708
	THSO 16	-0.290	-0.355	-0.225	0.017	42	-17.090	< .001***
THSO 9	AHSO 16	0.070	0.005	0.135	0.017	42	4.125	0.022*
	SHSO 16	0.030	-0.035	0.095	0.017	42	1.768	0.964
	THSO 16	-0.220	-0.285	-0.155	0.017	42	-12.965	< .001***
AHSO 16	SHSO 16	-0.040	-0.105	0.025	0.017	42	-2.357	0.708
	THSO 16	-0.290	-0.355	-0.225	0.017	42	-17.090	< .001***
SHSO 16	THSO 16	-0.250	-0.315	-0.185	0.017	42	-14.733	< .001***

* p < .05, ** p < .01, *** p < .001

Note. P-value and confidence intervals adjusted for comparing a family of 21 estimates (confidence intervals corrected using the tukey method).

Letter-Based Grouping - sample ✻ concentration

sample	concentration	Letter
AHSO	0.3	b
SHSO		c
THSO		a
AHSO	0.6	d
SHSO		d
THSO		d
AHSO	1.3	de
SHSO		d
THSO		d

Letter-Based Grouping - sample ✻ concentration

sample	concentration	Letter
AHSO	2.5	def
SHSO		def
THSO		de
AHSO	5	def
SHSO		def
THSO		fg
AHSO	9	def
SHSO		def
THSO		g
AHSO	16	def
SHSO		efg
THSO		h

Note. If two or more means share the same grouping symbol, then we cannot show them to be different, but we also did not show them to be the same.

ANOVA

ANOVA - Lactobacillus acidophilus ATCC 4356

Cases	Sum of Squares	df	Mean Square	F	p
sample	0.303	2	0.152	349.222	< .001
concentration	0.537	6	0.090	206.061	< .001
sample ✻ concentration	0.285	12	0.024	54.614	< .001
Residuals	0.018	42	4.345×10^{-4}		

Note. Type III Sum of Squares

Descriptives

Descriptives - Lactobacillus acidophilus ATCC 4356

sample	concentration	N	Mean	SD	SE	Coefficient of variation
AHSO	0.3	3	0.890	0.027	0.016	0.030
	0.6	3	0.940	0.015	0.009	0.016
	1.3	3	0.950	0.023	0.013	0.024
	2.5	3	0.960	0.016	0.009	0.017
	5	3	1.010	0.028	0.016	0.028
	9	3	1.010	0.026	0.015	0.026
	16	3	1.030	0.015	0.009	0.015
SHSO	0.3	3	0.880	0.013	0.008	0.015
	0.6	3	0.990	0.012	0.007	0.012
	1.3	3	1.020	0.018	0.010	0.018

Descriptives - Lactobacillus acidophilus ATCC 4356

sample	concentration	N	Mean	SD	SE	Coefficient of variation
	2.5	3	1.020	0.029	0.017	0.028
	5	3	1.030	0.017	0.010	0.017
	9	3	1.090	0.025	0.014	0.023
	16	3	1.110	0.027	0.016	0.024
THSO	0.3	3	0.960	0.019	0.011	0.020
	0.6	3	1.010	0.021	0.012	0.021
	1.3	3	1.070	0.024	0.014	0.022
	2.5	3	1.080	0.014	0.008	0.013
	5	3	1.140	0.022	0.013	0.019
	9	3	1.140	0.011	0.006	0.010
	16	3	1.550	0.020	0.012	0.013

Post Hoc Tests

Standard (HSD)

Post Hoc Comparisons - sample ✻ concentration

			95% CI for Mean Difference					
		Mean Difference	Lower	Upper	SE	df	t	p_{tukey}
AHSO 0.3	SHSO 0.3	0.010	-0.055	0.075	0.017	42	0.588	1.000
	THSO 0.3	-0.070	-0.135	-0.005	0.017	42	-4.113	0.022*
	AHSO 0.6	-0.050	-0.115	0.015	0.017	42	-2.938	0.328
	SHSO 0.6	-0.100	-0.165	-0.035	0.017	42	-5.876	< .001***
	THSO 0.6	-0.120	-0.185	-0.055	0.017	42	-7.051	< .001***
	AHSO 1.3	-0.060	-0.125	0.005	0.017	42	-3.525	0.099
	SHSO 1.3	-0.130	-0.195	-0.065	0.017	42	-7.638	< .001***
	THSO 1.3	-0.180	-0.245	-0.115	0.017	42	-10.576	< .001***
	AHSO 2.5	-0.070	-0.135	-0.005	0.017	42	-4.113	0.022*
	SHSO 2.5	-0.130	-0.195	-0.065	0.017	42	-7.638	< .001***
	THSO 2.5	-0.190	-0.255	-0.125	0.017	42	-11.164	< .001***
	AHSO 5	-0.120	-0.185	-0.055	0.017	42	-7.051	< .001***

Post Hoc Comparisons - sample ✲ concentration

		Mean Difference	95% CI for Mean Difference		SE	df	t	p_{tukey}
			Lower	Upper				
	SHSO 5	-0.140	-0.205	-0.075	0.017	42	-8.226	< .001***
	THSO 5	-0.250	-0.315	-0.185	0.017	42	-14.689	< .001***
	AHSO 9	-0.120	-0.185	-0.055	0.017	42	-7.051	< .001***
	SHSO 9	-0.200	-0.265	-0.135	0.017	42	-11.751	< .001***
	THSO 9	-0.250	-0.315	-0.185	0.017	42	-14.689	< .001***
	AHSO 16	-0.140	-0.205	-0.075	0.017	42	-8.226	< .001***
	SHSO 16	-0.220	-0.285	-0.155	0.017	42	-12.927	< .001***
	THSO 16	-0.660	-0.725	-0.595	0.017	42	-38.780	< .001***
SHSO 0.3	THSO 0.3	-0.080	-0.145	-0.015	0.017	42	-4.701	0.004**
	AHSO 0.6	-0.060	-0.125	0.005	0.017	42	-3.525	0.099
	SHSO 0.6	-0.110	-0.175	-0.045	0.017	42	-6.463	< .001***
	THSO 0.6	-0.130	-0.195	-0.065	0.017	42	-7.638	< .001***
	AHSO 1.3	-0.070	-0.135	-0.005	0.017	42	-4.113	0.022*
	SHSO 1.3	-0.140	-0.205	-0.075	0.017	42	-8.226	< .001***
	THSO 1.3	-0.190	-0.255	-0.125	0.017	42	-11.164	< .001***
	AHSO 2.5	-0.080	-0.145	-0.015	0.017	42	-4.701	0.004**
	SHSO 2.5	-0.140	-0.205	-0.075	0.017	42	-8.226	< .001***
	THSO 2.5	-0.200	-0.265	-0.135	0.017	42	-11.751	< .001***
	AHSO 5	-0.130	-0.195	-0.065	0.017	42	-7.638	< .001***
	SHSO 5	-0.150	-0.215	-0.085	0.017	42	-8.814	< .001***
	THSO 5	-0.260	-0.325	-0.195	0.017	42	-15.277	< .001***
	AHSO 9	-0.130	-0.195	-0.065	0.017	42	-7.638	< .001***
	SHSO 9	-0.210	-0.275	-0.145	0.017	42	-12.339	< .001***
	THSO 9	-0.260	-0.325	-0.195	0.017	42	-15.277	< .001***
	AHSO 16	-0.150	-0.215	-0.085	0.017	42	-8.814	< .001***
	SHSO 16	-0.230	-0.295	-0.165	0.017	42	-13.514	< .001***
	THSO 16	-0.670	-0.735	-0.605	0.017	42	-39.367	< .001***

Post Hoc Comparisons - sample ✻ concentration

		Mean Difference	95% CI for Mean Difference		SE	df	t	p_{tukey}
			Lower	Upper				
THSO 0.3	AHSO 0.6	0.020	-0.045	0.085	0.017	42	1.175	1.000
	SHSO 0.6	-0.030	-0.095	0.035	0.017	42	-1.763	0.965
	THSO 0.6	-0.050	-0.115	0.015	0.017	42	-2.938	0.328
	AHSO 1.3	0.010	-0.055	0.075	0.017	42	0.588	1.000
	SHSO 1.3	-0.060	-0.125	0.005	0.017	42	-3.525	0.099
	THSO 1.3	-0.110	-0.175	-0.045	0.017	42	-6.463	< .001***
	AHSO 2.5	3.313×10^{-16}	-0.065	0.065	0.017	42	1.947×10^{-14}	1.000
	SHSO 2.5	-0.060	-0.125	0.005	0.017	42	-3.525	0.099
	THSO 2.5	-0.120	-0.185	-0.055	0.017	42	-7.051	< .001***
	AHSO 5	-0.050	-0.115	0.015	0.017	42	-2.938	0.328
	SHSO 5	-0.070	-0.135	-0.005	0.017	42	-4.113	0.022*
	THSO 5	-0.180	-0.245	-0.115	0.017	42	-10.576	< .001***
	AHSO 9	-0.050	-0.115	0.015	0.017	42	-2.938	0.328
	SHSO 9	-0.130	-0.195	-0.065	0.017	42	-7.638	< .001***
	THSO 9	-0.180	-0.245	-0.115	0.017	42	-10.576	< .001***
	AHSO 16	-0.070	-0.135	-0.005	0.017	42	-4.113	0.022*
	SHSO 16	-0.150	-0.215	-0.085	0.017	42	-8.814	< .001***
	THSO 16	-0.590	-0.655	-0.525	0.017	42	-34.667	< .001***
AHSO 0.6	SHSO 0.6	-0.050	-0.115	0.015	0.017	42	-2.938	0.328
	THSO 0.6	-0.070	-0.135	-0.005	0.017	42	-4.113	0.022*
	AHSO 1.3	-0.010	-0.075	0.055	0.017	42	-0.588	1.000
	SHSO 1.3	-0.080	-0.145	-0.015	0.017	42	-4.701	0.004**
	THSO 1.3	-0.130	-0.195	-0.065	0.017	42	-7.638	< .001***
	AHSO 2.5	-0.020	-0.085	0.045	0.017	42	-1.175	1.000

Post Hoc Comparisons - sample ✻ concentration

		Mean Difference	95% CI for Mean Difference		SE	df	t	p_{tukey}
			Lower	Upper				
	SHSO 2.5	-0.080	-0.145	-0.015	0.017	42	-4.701	0.004**
	THSO 2.5	-0.140	-0.205	-0.075	0.017	42	-8.226	< .001***
	AHSO 5	-0.070	-0.135	-0.005	0.017	42	-4.113	0.022*
	SHSO 5	-0.090	-0.155	-0.025	0.017	42	-5.288	< .001***
	THSO 5	-0.200	-0.265	-0.135	0.017	42	-11.751	< .001***
	AHSO 9	-0.070	-0.135	-0.005	0.017	42	-4.113	0.022*
	SHSO 9	-0.150	-0.215	-0.085	0.017	42	-8.814	< .001***
	THSO 9	-0.200	-0.265	-0.135	0.017	42	-11.751	< .001***
	AHSO 16	-0.090	-0.155	-0.025	0.017	42	-5.288	< .001***
	SHSO 16	-0.170	-0.235	-0.105	0.017	42	-9.989	< .001***
	THSO 16	-0.610	-0.675	-0.545	0.017	42	-35.842	< .001***
SHSO 0.6	THSO 0.6	-0.020	-0.085	0.045	0.017	42	-1.175	1.000
	AHSO 1.3	0.040	-0.025	0.105	0.017	42	2.350	0.712
	SHSO 1.3	-0.030	-0.095	0.035	0.017	42	-1.763	0.965
	THSO 1.3	-0.080	-0.145	-0.015	0.017	42	-4.701	0.004**
	AHSO 2.5	0.030	-0.035	0.095	0.017	42	1.763	0.965
	SHSO 2.5	-0.030	-0.095	0.035	0.017	42	-1.763	0.965
	THSO 2.5	-0.090	-0.155	-0.025	0.017	42	-5.288	< .001***
	AHSO 5	-0.020	-0.085	0.045	0.017	42	-1.175	1.000
	SHSO 5	-0.040	-0.105	0.025	0.017	42	-2.350	0.712
	THSO 5	-0.150	-0.215	-0.085	0.017	42	-8.814	< .001***
	AHSO 9	-0.020	-0.085	0.045	0.017	42	-1.175	1.000
	SHSO 9	-0.100	-0.165	-0.035	0.017	42	-5.876	< .001***
	THSO 9	-0.150	-0.215	-0.085	0.017	42	-8.814	< .001***
	AHSO 16	-0.040	-0.105	0.025	0.017	42	-2.350	0.712
	SHSO 16	-0.120	-0.185	-0.055	0.017	42	-7.051	< .001***
	THSO 16	-0.560	-0.625	-0.495	0.017	42	-32.904	< .001***

Post Hoc Comparisons - sample ✻ concentration

		Mean Difference	95% CI for Mean Difference		SE	df	t	p_{tukey}
			Lower	Upper				
THSO 0.6	AHSO 1.3	0.060	-0.005	0.125	0.017	42	3.525	0.099
	SHSO 1.3	-0.010	-0.075	0.055	0.017	42	-0.588	1.000
	THSO 1.3	-0.060	-0.125	0.005	0.017	42	-3.525	0.099
	AHSO 2.5	0.050	-0.015	0.115	0.017	42	2.938	0.328
	SHSO 2.5	-0.010	-0.075	0.055	0.017	42	-0.588	1.000
	THSO 2.5	-0.070	-0.135	-0.005	0.017	42	-4.113	0.022*
	AHSO 5	-1.527×10^{-16}	-0.065	0.065	0.017	42	-8.970×10^{-15}	1.000
	SHSO 5	-0.020	-0.085	0.045	0.017	42	-1.175	1.000
	THSO 5	-0.130	-0.195	-0.065	0.017	42	-7.638	< .001***
	AHSO 9	-1.652×10^{-16}	-0.065	0.065	0.017	42	-9.709×10^{-15}	1.000
	SHSO 9	-0.080	-0.145	-0.015	0.017	42	-4.701	0.004**
	THSO 9	-0.130	-0.195	-0.065	0.017	42	-7.638	< .001***
	AHSO 16	-0.020	-0.085	0.045	0.017	42	-1.175	1.000
	SHSO 16	-0.100	-0.165	-0.035	0.017	42	-5.876	< .001***
	THSO 16	-0.540	-0.605	-0.475	0.017	42	-31.729	< .001***
AHSO 1.3	SHSO 1.3	-0.070	-0.135	-0.005	0.017	42	-4.113	0.022*
	THSO 1.3	-0.120	-0.185	-0.055	0.017	42	-7.051	< .001***
	AHSO 2.5	-0.010	-0.075	0.055	0.017	42	-0.588	1.000
	SHSO 2.5	-0.070	-0.135	-0.005	0.017	42	-4.113	0.022*
	THSO 2.5	-0.130	-0.195	-0.065	0.017	42	-7.638	< .001***
	AHSO 5	-0.060	-0.125	0.005	0.017	42	-3.525	0.099
	SHSO 5	-0.080	-0.145	-0.015	0.017	42	-4.701	0.004**
	THSO 5	-0.190	-0.255	-0.125	0.017	42	-11.164	< .001***
	AHSO 9	-0.060	-0.125	0.005	0.017	42	-3.525	0.099
	SHSO 9	-0.140	-0.205	-0.075	0.017	42	-8.226	< .001***
	THSO 9	-0.190	-0.255	-0.125	0.017	42	-11.164	< .001***

Post Hoc Comparisons - sample ✻ concentration

		Mean Difference	95% CI for Mean Difference		SE	df	t	p_{tukey}
			Lower	Upper				
	AHSO 16	-0.080	-0.145	-0.015	0.017	42	-4.701	0.004**
	SHSO 16	-0.160	-0.225	-0.095	0.017	42	-9.401	< .001***
	THSO 16	-0.600	-0.665	-0.535	0.017	42	-35.254	< .001***
SHSO 1.3	THSO 1.3	-0.050	-0.115	0.015	0.017	42	-2.938	0.328
	AHSO 2.5	0.060	-0.005	0.125	0.017	42	3.525	0.099
	SHSO 2.5	-6.245×10^{-17}	-0.065	0.065	0.017	42	-3.669×10^{-15}	1.000
	THSO 2.5	-0.060	-0.125	0.005	0.017	42	-3.525	0.099
	AHSO 5	0.010	-0.055	0.075	0.017	42	0.588	1.000
	SHSO 5	-0.010	-0.075	0.055	0.017	42	-0.588	1.000
	THSO 5	-0.120	-0.185	-0.055	0.017	42	-7.051	< .001***
	AHSO 9	0.010	-0.055	0.075	0.017	42	0.588	1.000
	SHSO 9	-0.070	-0.135	-0.005	0.017	42	-4.113	0.022*
	THSO 9	-0.120	-0.185	-0.055	0.017	42	-7.051	< .001***
	AHSO 16	-0.010	-0.075	0.055	0.017	42	-0.588	1.000
	SHSO 16	-0.090	-0.155	-0.025	0.017	42	-5.288	< .001***
	THSO 16	-0.530	-0.595	-0.465	0.017	42	-31.141	< .001***
THSO 1.3	AHSO 2.5	0.110	0.045	0.175	0.017	42	6.463	< .001***
	SHSO 2.5	0.050	-0.015	0.115	0.017	42	2.938	0.328
	THSO 2.5	-0.010	-0.075	0.055	0.017	42	-0.588	1.000
	AHSO 5	0.060	-0.005	0.125	0.017	42	3.525	0.099
	SHSO 5	0.040	-0.025	0.105	0.017	42	2.350	0.712
	THSO 5	-0.070	-0.135	-0.005	0.017	42	-4.113	0.022*
	AHSO 9	0.060	-0.005	0.125	0.017	42	3.525	0.099
	SHSO 9	-0.020	-0.085	0.045	0.017	42	-1.175	1.000
	THSO 9	-0.070	-0.135	-0.005	0.017	42	-4.113	0.022*
	AHSO 16	0.040	-0.025	0.105	0.017	42	2.350	0.712
	SHSO 16	-0.040	-0.105	0.025	0.017	42	-2.350	0.712

Post Hoc Comparisons - sample ✻ concentration

		Mean Difference	95% CI for Mean Difference		SE	df	t	p_{tukey}
			Lower	Upper				
	THSO 16	-0.480	-0.545	-0.415	0.017	42	-28.204	< .001***
AHSO 2.5	SHSO 2.5	-0.060	-0.125	0.005	0.017	42	-3.525	0.099
	THSO 2.5	-0.120	-0.185	-0.055	0.017	42	-7.051	< .001***
	AHSO 5	-0.050	-0.115	0.015	0.017	42	-2.938	0.328
	SHSO 5	-0.070	-0.135	-0.005	0.017	42	-4.113	0.022*
	THSO 5	-0.180	-0.245	-0.115	0.017	42	-10.576	< .001***
	AHSO 9	-0.050	-0.115	0.015	0.017	42	-2.938	0.328
	SHSO 9	-0.130	-0.195	-0.065	0.017	42	-7.638	< .001***
	THSO 9	-0.180	-0.245	-0.115	0.017	42	-10.576	< .001***
	AHSO 16	-0.070	-0.135	-0.005	0.017	42	-4.113	0.022*
	SHSO 16	-0.150	-0.215	-0.085	0.017	42	-8.814	< .001***
	THSO 16	-0.590	-0.655	-0.525	0.017	42	-34.667	< .001***
SHSO 2.5	THSO 2.5	-0.060	-0.125	0.005	0.017	42	-3.525	0.099
	AHSO 5	0.010	-0.055	0.075	0.017	42	0.588	1.000
	SHSO 5	-0.010	-0.075	0.055	0.017	42	-0.588	1.000
	THSO 5	-0.120	-0.185	-0.055	0.017	42	-7.051	< .001***
	AHSO 9	0.010	-0.055	0.075	0.017	42	0.588	1.000
	SHSO 9	-0.070	-0.135	-0.005	0.017	42	-4.113	0.022*
	THSO 9	-0.120	-0.185	-0.055	0.017	42	-7.051	< .001***
	AHSO 16	-0.010	-0.075	0.055	0.017	42	-0.588	1.000
	SHSO 16	-0.090	-0.155	-0.025	0.017	42	-5.288	< .001***
	THSO 16	-0.530	-0.595	-0.465	0.017	42	-31.141	< .001***
THSO 2.5	AHSO 5	0.070	0.005	0.135	0.017	42	4.113	0.022*
	SHSO 5	0.050	-0.015	0.115	0.017	42	2.938	0.328
	THSO 5	-0.060	-0.125	0.005	0.017	42	-3.525	0.099
	AHSO 9	0.070	0.005	0.135	0.017	42	4.113	0.022*
	SHSO 9	-0.010	-0.075	0.055	0.017	42	-0.588	1.000
	THSO 9	-0.060	-0.125	0.005	0.017	42	-3.525	0.099
	AHSO 16	0.050	-0.015	0.115	0.017	42	2.938	0.328
	SHSO 16	-0.030	-0.095	0.035	0.017	42	-1.763	0.965

Post Hoc Comparisons - sample ✻ concentration

		Mean Difference	95% CI for Mean Difference		SE	df	t	p_{tukey}
			Lower	Upper				
	THSO 16	-0.470	-0.535	-0.405	0.017	42	-27.616	< .001***
AHSO 5	SHSO 5	-0.020	-0.085	0.045	0.017	42	-1.175	1.000
	THSO 5	-0.130	-0.195	-0.065	0.017	42	-7.638	< .001***
	AHSO 9	-1.258×10^{-17}	-0.065	0.065	0.017	42	-7.390×10^{-16}	1.000
	SHSO 9	-0.080	-0.145	-0.015	0.017	42	-4.701	0.004**
	THSO 9	-0.130	-0.195	-0.065	0.017	42	-7.638	< .001***
	AHSO 16	-0.020	-0.085	0.045	0.017	42	-1.175	1.000
	SHSO 16	-0.100	-0.165	-0.035	0.017	42	-5.876	< .001***
	THSO 16	-0.540	-0.605	-0.475	0.017	42	-31.729	< .001***
SHSO 5	THSO 5	-0.110	-0.175	-0.045	0.017	42	-6.463	< .001***
	AHSO 9	0.020	-0.045	0.085	0.017	42	1.175	1.000
	SHSO 9	-0.060	-0.125	0.005	0.017	42	-3.525	0.099
	THSO 9	-0.110	-0.175	-0.045	0.017	42	-6.463	< .001***
	AHSO 16	1.431×10^{-17}	-0.065	0.065	0.017	42	8.409×10^{-16}	1.000
	SHSO 16	-0.080	-0.145	-0.015	0.017	42	-4.701	0.004**
	THSO 16	-0.520	-0.585	-0.455	0.017	42	-30.554	< .001***
THSO 5	AHSO 9	0.130	0.065	0.195	0.017	42	7.638	< .001***
	SHSO 9	0.050	-0.015	0.115	0.017	42	2.938	0.328
	THSO 9	1.431×10^{-17}	-0.065	0.065	0.017	42	8.409×10^{-16}	1.000
	AHSO 16	0.110	0.045	0.175	0.017	42	6.463	< .001***
	SHSO 16	0.030	-0.035	0.095	0.017	42	1.763	0.965
	THSO 16	-0.410	-0.475	-0.345	0.017	42	-24.091	< .001***
AHSO 9	SHSO 9	-0.080	-0.145	-0.015	0.017	42	-4.701	0.004**
	THSO 9	-0.130	-0.195	-0.065	0.017	42	-7.638	< .001***
	AHSO 16	-0.020	-0.085	0.045	0.017	42	-1.175	1.000
	SHSO 16	-0.100	-0.165	-0.035	0.017	42	-5.876	< .001***
	THSO 16	-0.540	-0.605	-0.475	0.017	42	-31.729	< .001***
SHSO 9	THSO 9	-0.050	-0.115	0.015	0.017	42	-2.938	0.328

Post Hoc Comparisons - sample ✲ concentration

		Mean Difference	95% CI for Mean Difference		SE	df	t	p_{tukey}
			Lower	Upper				
	AHSO 16	0.060	-0.005	0.125	0.017	42	3.525	0.099
	SHSO 16	-0.020	-0.085	0.045	0.017	42	-1.175	1.000
	THSO 16	-0.460	-0.525	-0.395	0.017	42	-27.028	< .001***
THSO 9	AHSO 16	0.110	0.045	0.175	0.017	42	6.463	< .001***
	SHSO 16	0.030	-0.035	0.095	0.017	42	1.763	0.965
	THSO 16	-0.410	-0.475	-0.345	0.017	42	-24.091	< .001***
AHSO 16	SHSO 16	-0.080	-0.145	-0.015	0.017	42	-4.701	0.004**
	THSO 16	-0.520	-0.585	-0.455	0.017	42	-30.554	< .001***
SHSO 16	THSO 16	-0.440	-0.505	-0.375	0.017	42	-25.853	< .001***

* p < .05, ** p < .01, *** p < .001

Note. P-value and confidence intervals adjusted for comparing a family of 21 estimates (confidence intervals corrected using the tukey method).

Letter-Based Grouping - sample ✲ concentration

sample	concentration	Letter
AHSO	0.3	ab
SHSO		a
THSO		cde
AHSO	0.6	abc
SHSO		cdef
THSO		defg
AHSO	1.3	bcd
SHSO		efgh
THSO		ghij
AHSO	2.5	cde
SHSO		efgh
THSO		hijk
AHSO	5	defg
SHSO		fghi
THSO		k
AHSO	9	defg

Letter-Based Grouping - sample ✲ concentration

sample	concentration	Letter
SHSO		ijk
THSO		k
AHSO	16	fghi
SHSO		jk
THSO		l

Note. If two or more means share the same grouping symbol, then we cannot show them to be different, but we also did not show them to be the same.
