# Supplementary material for: Chemical Profile, Bioactive Constituents and In Vitro Growth Stimulation Properties of Cold-Pressed Hemp Seed Oils from Romanian Varieties: In Vitro and In Silico Evaluation
Source: Plants (Basel). 2025 Nov 13;14(22):3465. doi: 10.3390/plants14223465 (PMC12655998; doi:10.3390/plants14223465)
Supplement: Supplementary file 1 [file plants-14-03465-s001.zip › Supplementary file S3_FAME area tables, TPC DPPH replicate values, and raw per-well OD.pdf]

Supplementary file S3

FAME raw data

Sample	Replicate	C16:0	C16:1	C17:0	C18:0	C18:1ω9	C18:2	C18:3 ϒ	C18:3 α	C18:1ω7	C 20:1	C20:0
THSO	R1	7.008	0.063	0.024	3.194	22.96	51.8	2.463	9.852	1.045	0.277	0.795
THSO	R2	6.97	0.07	0.018	3.107	22.82	51.4	2.55	9.944	1.1	0.345	0.75
THSO	R3	7.175	0.055	0.03	3.28	22.56	51.335	2.377	9.759	1.079	0.255	0.81
SHSO	R1	6.446	0.07	0.022	3.405	16.45	51.809	3.591	14.368	1.06	0.321	0.835
SHSO	R2	6.548	0.09	0.019	3.45	16.36	51.98	3.486	14.238	1.008	0.292	0.88
SHSO	R3	6.6	0.06	0.049	3.55	16.22	51.786	3.695	14.488	0.975	0.35	0.816
AHSO	R1	7.91	0.089	0.025	3.577	18.769	49.445	3.15	12.6	1.4	0.485	1.024
AHSO	R2	8.049	0.098	0.015	3.487	18.77	49.525	2.97	12.519	1.357	0.412	1.012
AHSO	R3	7.725	0.079	0.035	3.66	18.552	49.303	3.33	12.682	1.22	0.33	1.035

Sample	Replicate	C24:0	C22:0	C20:4	unassigned C20 PUFA	SFA	MUFA	PUFA	PUFA/SFA	ω3	ω6	ω-6 / ω-3
THSO	R1	0.05	0.22	0.502	0.14	11.291	24.345	64.757	5.735	9.852	54.765	5.559
THSO	R2	0.035	0.209	0.5	0.126	11.089	24.335	64.52	5.818	9.944	54.45	5.476
THSO	R3	0.064	0.19	0.53	0.155	11.549	23.949	64.156	5.555	9.759	54.242	5.558
SHSO	R1	0.08	0.2	0.93	0.355	10.988	17.901	71.053	6.466	14.368	56.33	3.921
SHSO	R2	0.034	0.31	0.913	0.393	11.241	17.75	71.01	6.317	14.238	56.379	3.96
SHSO	R3	0.025	0.105	0.97	0.367	11.145	17.605	71.306	6.398	14.488	56.451	3.896
AHSO	R1	0.055	0.278	1.092	0.338	12.869	20.743	66.633	5.178	12.608	53.687	4.258
AHSO	R2	0.112	0.3	1.12	0.36	12.975	20.637	66.478	5.124	12.503	53.615	4.288
AHSO	R3	0.07	0.27	1.034	0.325	12.795	20.181	66.682	5.212	12.69	53.667	4.229

Table of triplicate values recorded for TPC and DPPH

Sample	Replicate	TPC	DPPH
THSO	R1	159.99	43.31
THSO	R2	159.99	43.37
THSO	R3	159.76	43.43
SHSO	R1	147.8	38.15
SHSO	R2	148.49	38.25
SHSO	R3	148.49	38.35
AHSO	R1	119.06	35.78
AHSO	R2	119.06	35.92
AHSO	R3	118.01	36.06

Table Raw data per-well OD

	Lacticaseibacillus rhamnosus (GG; HN001)												
		THSO				SHSO				AHSO			
	Replicate 1	Replicate 2	Replicate 3	Mean	Replicate 1	Replicate 2	Replicate 3	Mean	Replicate 1	Replicate 2	Replicate 3	Mean	
0.3	0.515	0.548	0.53	0.531	0.447	0.472	0.461	0.46	0.684	0.706	0.701	0.697	
0.6	0.788	0.841	0.81	0.813	0.758	0.799	0.786	0.781	0.788	0.817	0.804	0.803	
1.3	0.846	0.801	0.828	0.825	0.793	0.835	0.82	0.816	0.793	0.836	0.814	0.814	
2.5	0.819	0.864	0.843	0.842	0.844	0.804	0.821	0.823	0.851	0.816	0.814	0.827	
5	0.848	0.893	0.869	0.87	0.904	0.867	0.875	0.882	0.81	0.846	0.837	0.831	
9	0.921	0.978	0.948	0.949	0.874	0.927	0.899	0.9	0.821	0.857	0.849	0.842	
16	0.939	0.992	0.973	0.968	1.07	1.139	1.104	1.104	0.906	0.854	0.88	0.88	
	Lactobacillus paracasei subsp.paracasei ATCC BAA-52												
		THSO				SHSO				AHSO			
	Replicate 1	Replicate 2	Replicate 3	Mean	Replicate 1	Replicate 2	Replicate 3	Mean	Replicate 1	Replicate 2	Replicate 3	Mean	
0.3	0.447	0.474	0.459	0.46	0.715	0.683	0.693	0.697	0.646	0.62	0.627	0.631	
0.6	0.757	0.799	0.787	0.781	0.82	0.788	0.802	0.803	0.794	0.829	0.825	0.816	
1.3	0.793	0.839	0.816	0.816	0.794	0.828	0.82	0.814	0.842	0.808	0.825	0.825	
2.5	0.806	0.844	0.819	0.823	0.845	0.809	0.827	0.827	0.803	0.846	0.835	0.828	

5	0.865	0.905	0.876	0.882	0.81	0.857	0.826	0.831	0.853	0.814	0.821	0.829	
9	0.876	0.926	0.898	0.9	0.864	0.825	0.837	0.842	0.81	0.852	0.828	0.83	
16	1.071	1.135	1.107	1.104	0.853	0.897	0.89	0.88	0.855	0.819	0.836	0.837	
	Lactobacillus acidophilus ATCC 4356												
		THSO				SHSO				AHSO			
	Replicate 1	Replicate 2	Replicate 3	Mean	Replicate 1	Replicate 2	Replicate 3	Mean	Replicate 1	Replicate 2	Replicate 3	Mean	
0.3	0.127	0.134	0.132	0.131	0.118	0.124	0.121	0.121	0.125	0.12	0.121	0.122	
0.6	0.143	0.135	0.139	0.139	0.14	0.132	0.136	0.136	0.127	0.133	0.127	0.129	
1.3	0.142	0.15	0.146	0.146	0.136	0.143	0.141	0.14	0.128	0.133	0.129	0.13	
2.5	0.144	0.151	0.149	0.148	0.142	0.137	0.141	0.14	0.136	0.129	0.132	0.132	
5	0.16	0.152	0.156	0.156	0.138	0.143	0.142	0.141	0.141	0.135	0.138	0.138	
9	0.153	0.159	0.156	0.156	0.154	0.147	0.149	0.15	0.142	0.136	0.139	0.139	
16	0.219	0.208	0.209	0.212	0.156	0.148	0.152	0.152	0.138	0.145	0.14	0.141	

Mean Blank OD_{600} (±SD)

0.045 ± 0.002

0.050 ± 0.003

0.052 ± 0.003

0.055 ± 0.003

0.058 ± 0.004

0.061 ± 0.004

0.065 ± 0.004
